# Routes to Cyclobutadiene and Cycloallyl Pt(II) Complexes: Dicationic Bis(Alkyne) Species as Intermediates

**DOI:** 10.1002/chem.71204

**Published:** 2026-06-04

**Authors:** Ouchan He, Froze Jameel, Y. Einar H. Stampehl, Liza M. Richter, Matteo Tosi, Martin Kaupp, Thomas Braun

**Affiliations:** ^1^ Department of Chemistry Humboldt‐Universität Zu Berlin Berlin Germany; ^2^ Department of Chemistry Technische Universität Berlin Berlin Germany; ^3^ Institute for Molecular Systems Engineering and Advanced Materials, Heidelberg University Heidelberg Germany

**Keywords:** cycloallyl complexes, cyclobutadiene, dicationic complexes, platinum

## Abstract

Transition metal alkyne complexes can be crucial intermediates for a variety of transformations. Cyclobutadiene can be generated by metal‐mediated C–C coupling reactions, but this often requires an elaborate synthetic strategy. As a result, only a few examples of Pt(II) cyclobutadiene complexes have been described so far. Herein, we report on the development of a synthetic route to access cationic Pt(II) *η* ^3^‐cycloallyl complexes [Pt(*η* ^3^‐(C_2_H_5_ *C*)_3_C═C_2_H_4_)(L)][BF_4_] bearing various chelating diphosphine ligands (L). Their generation and its dependence on the choice of L were studied using a combined experimental and quantum chemical approach. Dicationic bis(alkyne) BF_4_ complexes are essential intermediates for the generation of *η* ^3^‐cycloallyl compounds. An investigation on the reactivity of Pt(II) *η* ^3^‐cycloallyl complexes toward the Br*ø*nsted acids HCl and HBF_4_·Et_2_O led to the formation of cationic and dicationic Pt(II) *η* ^4^‐cyclobutadiene complexes.

## Graphical abstract

This work demonstrates the synthesis of cationic Pt(II) *η* ^3^‐cycloallyl complexes, and experimental and computational studies show a ligand bite‐angle dependence of their formation rates. The reactivity of these complexes with Brønsted acids was investigated, leading to the formation of cationic and dicationic Pt(II) *η* ^4^‐cyclobutadiene complexes.

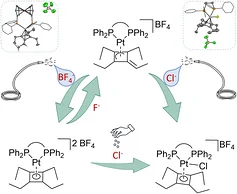

## 1 Introduction

Transition metal alkyne complexes often play a crucial role as key intermediates in a wide range of transformations enabled by the activation of the alkyne upon coordination to the metal centre. These processes include the functionalization of alkynes by the addition of nucleophiles [1, 2, 3, 4, 5, 6] as well as cyclization or annulation steps at a transition metal‐centre resulting in alkyne‐alkyne C−C bond formation [7, 8, 9, 10, 11, 12, 13, 14]. Recently, we reported on a cyclometalation of two alkynes at a Pt(II) centre, which was followed by an exocyclic deprotonation to furnish cycloallyl complexes. The products were identified as off‐cycle products of a Pt(II) catalysed hydrofluorination of alkynes [15]. To the best of our knowledge, cyclometalation followed by a subsequent ligand deprotonation without explicit addition of a base has only been demonstrated by Mashima and co‐workers at a Pd(II) centre in 2003 and recently by our group at a Pt(II) centre [15, 16].

Mechanistic studies of alkyne‐alkyne couplings by Pt complexes leading to the formation of *η* ^4^‐cyclobutadiene complexes are scarce [17, 18, 19, 20, 21, 22, 23, 24]. Investigations on the C–H bond activation of cyclic ligands, often named as ‘ring methyl activation' have been reported in the literature, but the conversions often require an external base or dioxygen. Noteworthy examples include the activation of a Cp* (pentamethylcyclopentadienyl) ligand coordinated at Ti [25, 26], Ta [27], and at Ru centres [28] or a *η* ^6^‐C_6_Me_6_ (hexamethylbenzene) ligand activation at Mn [29], Fe [30, 31, 32, 33, 34], and Ta [35]. In 2002, Mashima and coworkers reported on the first example of ring methyl activation of a *η* ^4^‐cyclobutadiene ligand at a Pd centre [36]. However, investigations into the steric and electronic effects to give cyclobutadiene and cycloallyl complexes by a subsequent deprotonation remain rather unexplored.

In this work, we describe the synthesis of Pt(II) *η* ^3^‐cycloallyl complexes of the type [Pt(*η* ^3^‐(C_2_H_5_ *C*)_3_C═C_2_H_4_)(L)][BF_4_] (**2**) and investigate experimentally and computationally the influence of the bidentate phosphine ligands on their formation. Quantum‐chemical calculations were performed to confirm experimental observations on the phosphine ligand dependence. The free energy barriers for the reaction pathways with dicationic bis(hexyne) complexes as intermediates to yield Pt(II) *η* ^3^‐cycloallyl complexes were determined. Its formation occurs by deprotonation with the BF_4_^−^ anion. The conversion of **2** into cationic and unique dicationic cyclobutadiene Pt(II) complexes was achieved by treatment with Br*ø*nsted acids.

## 2 Results and Discussion

**FIGURE 1:**
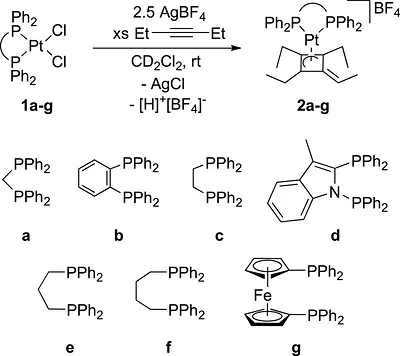
Formation of the complexes [Pt(*η* ^3^‐(C_2_H_5_ *C*)_3_C═C_2_H_4_)(L)][BF_4_] (**2a‐g**) bearing different bisphosphine ligands from 3‐hexyne.

Treatment of the Pt(II) dichlorido complexes [Pt(Cl)_2_(L)] (**1**) with AgBF_4_ and an excess of 3‐hexyne led to the formation of the cationic cycloallyl complexes [Pt(*η* ^3^‐(C_2_H_5_ *C*)_3_C═C_2_H_4_)(L)][BF_4_] (**2**) bearing an *exo*‐cyclic C═C double bond (Figure 1). L represents a variety of bisphosphine ligands that differ in their bite angle. The complexes [Pt(*η* ^3^‐(C_2_H_5_ *C*)_3_C═C_2_H_4_)(L)][BF_4_] (**2a**‐**g**) were synthesised and characterised according to a previously reported method [15] using the ligands (L) 1,1‐bis(diphenylphosphino)methane (dppm: **a**), 1,2‐bis(diphenylphosphino)benzene (dppbz: **b**), 1,2‐(bis(diphenylphosphino)ethane (dppe: **c**), 1,2‐bis(diphenylphosphino)‐3‐methyl‐1H‐indole (PCNP: **d**), 1,3‐bis(diphenylphosphino)propane (dppp: **e**), 1,4‐bis(diphenylphosphino)butane (dppb: **f**) and 1,1‐bis(diphenylphosphino)ferrocene (dppf: **g**) (Figure 1). It can be assumed that chlorido‐bridged binuclear species [Pt{(μ‐Cl)(L)}][BF_4_]_2_, as well as dicationic Pt(II) bis(alkyne) complexes, are formed as intermediates [15]. The structure in the solid state of [PtCl(dppm)]_2_[BF_4_]_2_·CHCl_3_ is given in the Supporting Information (see Figure S1). The *exo*‐cyclic double bond of the cycloallyl complex **2a**‐**g** results from in situ HBF_4_ formation, which converts further with excess of alkyne in the reaction mixture to give g*em*‐difluoroalkanes [15]. The latter can be easily removed under reduced pressure.

The ^31^P{^1^H} NMR spectrum of [Pt(*η* ^3^‐(C_2_H_5_ *C*)_3_C═C_2_H_4_)(dppm)][BF_4_] (**2a**) shows two doublets (δ = ‐20.3 ppm and ‐20.9 ppm, ^2^ *J*_P,P_ = 17 Hz) with ^195^Pt satellites and ^1^ *J*_P,Pt_ coupling constants of 3543 and 3582 Hz, respectively. The ^1^H NMR spectrum indicates the presence of diastereotopic protons for the bridging CH_2_ moiety between the phosphorous atoms. Their signals at δ = 5.14 ppm and 4.81 ppm appear as doublet of doublet of doublets with ^195^Pt satellites due to their mutual coupling (^2^ *J*_H,H_ = 16 Hz), the coupling to both phosphorous atoms (^2^ *J*_H,P_ = 10 Hz and ^2^ *J*_H,P_ = 11 Hz) and to the platinum nuclei (^3^ *J*_H,Pt_ = 113 Hz and ^3^ *J*_H,Pt_ = 23 Hz). The quartet at δ = 5.25 ppm with overlapping ^195^Pt satellites can be assigned to the *exo*‐cyclic CH proton with a coupling to the metal centre and the neighbouring methyl group. ^1^H,^1^H COSY NMR experiments of [Pt(*η* ^3^‐(C_2_H_5_ *C*)_3_C═C_2_H_4_)(dppm)][BF_4_] (**2a**) showed a cross peak to a signal at δ = 1.70 ppm, which can be assigned to the neighbouring methyl group. The multiplicity of the latter resonance as a doublet with ^195^Pt satellites (*J*_H,Pt_ = 10 Hz) suggests through‐space interaction to the platinum centre. Moreover, three additional triplets can be assigned to the other three methyl groups. The NMR data for the complexes **2c**‐**g** are comparable, and the data can be found in the Supporting Information.

**FIGURE 2:**
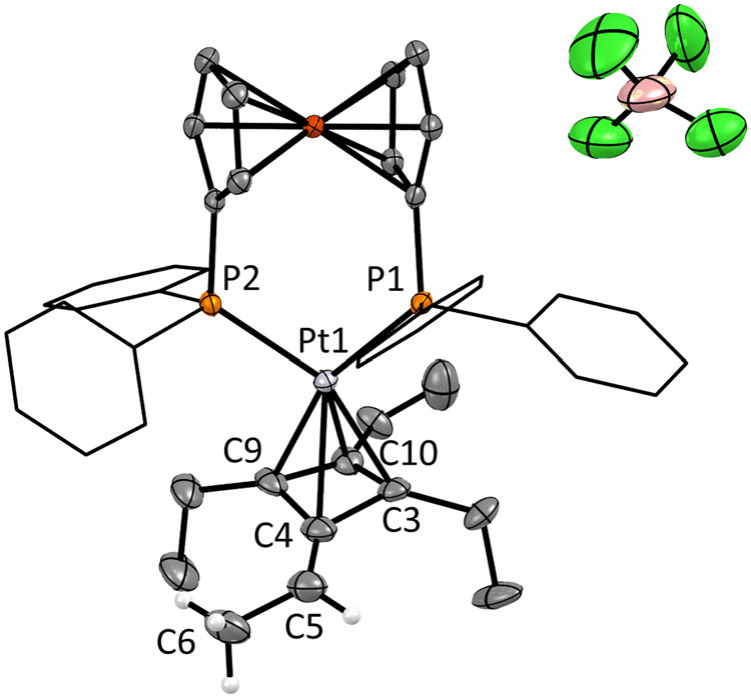
Structure of [Pt(η^3^‐(C_2_H_5_ *C*)_3_C=C_2_H_4_)(dppf)][BF_4_]·CH_2_Cl_2_ (**2g**·CH_2_Cl_2_) in the solid state. Phenyl substituents are depicted as wireframes. The solvent molecule is omitted for clarity. Thermal ellipsoids of the molecular structures are drawn at 50% probability level. Selected bond lengths [Å] and angle [°]: Pt1–C3 2.203(5), Pt1–C4 2.436(6), Pt1–C9 2.194(5), Pt1–C10 2.249(5), C3–C4 1.495(8), C3–C10 1.452(8), C4–C9 1.505(9), C9–C10 1.448(8), C4–C5 1.355(9); P1–Pt1–P2 101.89(4).

Suitable single crystals for x‐ray diffraction of [Pt(*η* ^3^‐(C_2_H_5_ *C*)_3_C═C_2_H_4_)(dppp)][BF_4_] (**2e**), [Pt(*η* ^3^‐(C_2_H_5_ *C*)_3_C═C_2_H_4_)(dppb)][BF_4_] (**2f**) and [Pt(*η* ^3^‐(C_2_H_5_ *C*)_3_C═C_2_H_4_)(dppf)][BF_4_] (**2g**) were obtained and the structures are depicted in Figure S2 for **2e**·CH_2_Cl_2_, in Figure S3 for **2f**·CH_2_Cl_2_ (see Supporting Information) and in Figure 2 for **2g**·CH_2_Cl_2_. Complex **2g**·CH_2_Cl_2_ crystallises in the monoclinic space group *P*2_1_/*n*. The structure reveals a square‐planar coordination at the platinum centre. The P1–Pt1–P2 bite angle of 101.89(4)° is comparable to the one in the Pt(II) dichlorido complex  **1g** (99.3(1)°) [37]. The bond lengths within the cycloallyl ligand of C3–C4 (1.495(8) Å), C3–C10 (1.452(8) Å), C4–C9 (1.505(9) Å), C9–C10 (1.448(8) Å) and C4–C5 (1.355(9) Å) are in good agreement with those in the previously reported Pt(II) cycloallyl complex [Pt(*η* ^3^‐(C_2_H_5_ *C*)_3_C═C_2_H_4_)(dppe)][BF_4_] [15]. Electron density found in the difference Fourier map is assigned to the CH proton of the ethenyl moiety.

**SCHEME 1:**
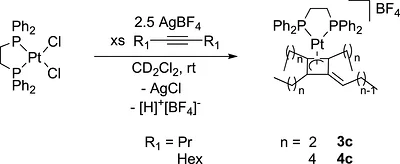
Formation of the complexes **3c** and **4c** with 4‐octyne and 6‐dodecyne as substrates.

When using longer chain internal alkynes such as 4‐octyne and 6‐dodecyne, the complexes [Pt(*η* ^3^‐(C_3_H_7_ *C*)_3_C═C_3_H_6_)(dppe)][BF_4_] (**3c**) and [Pt(*η* ^3^‐(C_5_H_11_ *C*)_3_C═C_5_H_10_)(dppe)][BF_4_] (**4c**) are formed (Scheme 1). For both complexes, the olefinic proton appears as a triplet with satellites at δ = 5.25 ppm in the ^1^H NMR spectra (^3^ *J*_H,Pt_ = 11 Hz, ^2^ *J*_H,H_ = 8 Hz) due to coupling to the adjacent CH_2_ group. ESI‐MS measurements further corroborated the identity of complexes **2a**‐**g**, **3c,** and **4c**.

**FIGURE 3:**
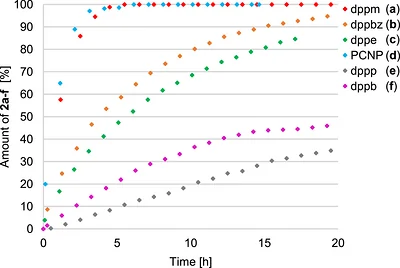
Reaction monitoring of the formation of [Pt(*η* ^3^‐(C_2_H_5_ *C*)_3_C═C_2_H_4_)(L)][BF_4_] (**2a‐f**) (L = dppm (**a**): red, dppbz (**b**): orange, dppe (**c**): green, PCNP (**d**): blue, dppp (**e**): gray, dppb (**f**): pink) by ^31^P{^1^H} NMR spectroscopy.

The formation of complexes **2a**‐**f** was also monitored by ^31^P{^1^H} NMR spectroscopy, starting from the dichlorido precursors [Pt(Cl)_2_(L)] (**1a**‐**f**) (Figure 3). The bidentate phosphine ligands are characterised by increasing P–Pt–P bite angles in **1** from dppm (**a**) to dppb (**f**) (**a**‐**f**: 74.27(7)° – 99.3(1)°) [15]. In the case for dppf (**g**) the formation of the cycloallyl complex **2** **g** was too slow to be considered for the NMR monitoring reaction. The sum of integration of the signals of the chlorido bridged dimers and the complexes **2a**‐**f** in the ^31^P{^1^H} NMR spectra was taken to remain constant in situ. Overall, the amount for product formation over time of the complexes **2a**‐**g** exhibit the following ligand‐dependency: **a** ∼ **d** > **b** > **c** > **f** > **e** > **g** (Figure 3). Roughly, a slower formation of complexes **2a**‐**g** is observed with increasing bite angle from dppm (**a**) to dppf (**g**) in complex **1**. For **1a** and **1d** a similar rate of formation to give the corresponding cycloallyl complexes **2a** and **2d** was found. Interestingly, the formation of **2b** proceeds more slowly but still quicker than **2c** in spite of comparable bite angles as for PCNP (**d**) (**1c**: 86.3(2)°, **1d**: 88.83(4)°). These observations suggest that besides the bite angle the rigidity in the backbone, in addition to the electronic properties, can have an impact on the formation of the complexes **2a**‐**g**. The rate was further diminished when using dppp (**e**) and dppb (**f**) as ligand systems. Opposed to the order of the bite angles in **1e** and **1f** (**1e**: 95.37(5)°, **1f**: 99.3(1)°) the formation of **2f** proceeds faster than the generation of **2e**. The initial rates have been determined (see Table S1, Supporting Information) and correlate with the reaction times. The ligand‐dependence was further confirmed by quantum‐chemical calculations.

**FIGURE 4:**
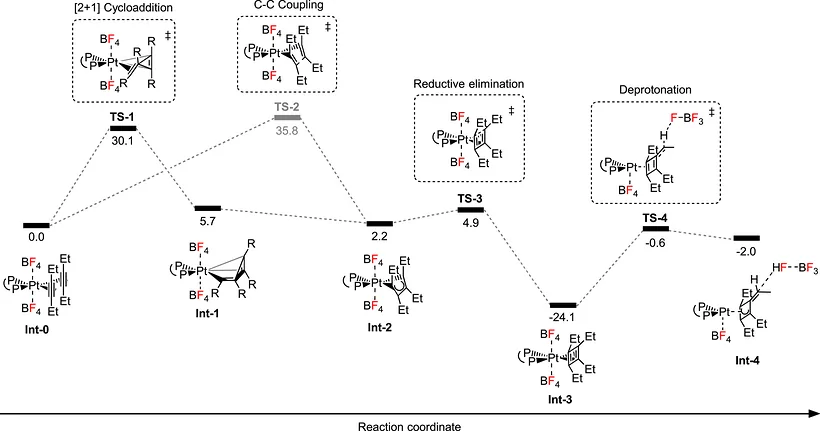
DFT calculated free energy profile in kcal·mol^−^^1^ at 298 K for the formation of cycloallyl (right) complexes from [Pt(C,C‐*η* ^2^‐C_2_H_5_ *C*≡*C*C_2_H_5_)_2_(dppp)][BF_4_]_2_ (**Int‐0**). The energies were calculated at ωB97M‐V/def2‐TZVP//BP86‐D3(BJ)/def2‐TZVP level of theory with internal thermal and entropic contributions at BP86‐D3(BJ)/def2‐TZVP level and solvation contributions at BP86‐D3(BJ)/def2‐TZVPD‐COSMO‐RS level (with 1,2‐dichloroethane), relative to **Int‐0**.

DFT calculations have been performed to investigate the effect of steric and electronic properties of the bidentate phosphine ligands coordinated to the platinum centre on the formation of the corresponding cycloallyl complex, starting from the dicationic bis(alkyne) complex intermediates (Scheme S1, Supporting Information), at ωB97M‐V/def2‐TZVP//BP86‐D3(BJ)/def2‐TZVP+COSMO‐RS (1,2‐dichloroethane) level. Figure 4 shows the free energy profile including all transition states leading to the Pt(II) cycloallyl complex exemplarily for dppp (**e**) as ligand. Computational details and the free energy profiles for the other investigated ligands (**a**, **b**, **c**, **d, f**) (Figure S61) are given in the Supporting Information.

The transformation of [Pt(C,C‐*η* ^2^‐C_2_H_5_C≡CC_2_H_5_)_2_(L)][BF_4_]_2_ (**Int‐0**) into a cycloallyl complex·HBF_4_ (**Int‐4**) is overall slightly exergonic in solution ($\Delta G_{0\;\to 4}$ = ‐2 kcal·mol^−^^1^). The reaction starts with the formation of a metallacycle complex (**Int‐2**), either *via* a direct C‐C coupling of the two alkyne ligands ($\Delta G_{C-C\;coup.}^{‡}$ = 35.8 kcal·mol^−^^1^) in **Int‐0** or *via* a [2+1] cycloaddition ($\Delta G_{[2+1]cyc.\;add.}^{‡}$= 30.1 kcal·mol^−^^1^) where a cyclopropene complex (**Int‐1**) is initially generated ($\Delta G_{0\;\to 1}$ = 5.7 kcal·mol^−^^1^) that later is converted to the metallacycle (**Int‐2**) complex ($\Delta G_{1\;\to 2}$ = ‐3.6 kcal·mol^−^^1^). Note that dppp gives the largest barriers, while other ligands provide lower ones between 27 and 30 kcal/mol (Figure S61, Supporting Information; see discussion below). One usually assumes that free‐energy barriers around 20 kcal/mol are consistent with reactions that take place within hours at room temperature. The computations thus apparently overestimate the barriers somewhat. It seems reasonable to assume that solvation or counter‐ion effects that go beyond the computational level employed may be responsible for lower barriers in such a complex reaction involving ion pairs. Below, we will therefore concentrate on trends with different ligands rather than absolute barriers. The direct formation of the metallacycle (**Int‐0**) complex as a result of C–C coupling is mildly endergonic ($\Delta G_{0\;\to 2}$ = 2.2 kcal·mol^−^^1^) in solution. [2+1] cycloaddition requires the rotation of one of the two alkyne ligands coordinated to the metal (**TS‐1**), whereas for direct C–C coupling, the rotation of both alkynes is a prerequisite (**TS‐2**). In the transition states, the Pt‐alkyne π‐coordination changes to a Pt–C σ‐bond upon the elongation of the Pt–C distances of the coupling atoms.

To generate a cyclobutadiene complex (**Int‐3**), another C–C bond must form to build a C_4_ ring. The formation of a Pt(II) *η* ^4^‐cyclobutadiene complex (**Int‐3**) is thermodynamically driven ($\Delta G_{2\;\to 3}$ = −26.2 kcal·mol^−^^1^) and requires a small activation energy (**TS‐3**, $\Delta G_{red.\;eli.}^{‡}$ = 2.7 kcal·mol^−^^1^). Finally, the cycloallyl complex is formed by abstraction of a proton (**TS‐4**) from the carbon adjacent to the cyclobutadiene ring. At the given computational level, this deprotonation of **Int‐3** by BF_4_^−^ is computed to be endergonic by about 20 kcal/mol, suggesting that the deprotonation equilibrium is shifted toward the cyclobutadiene complex. However, the formed HBF_4_, which is better described as HF bound loosely to BF_3_, is known to be consumed in the exergonic hydrofluorination of the alkynes. This will shift the deprotonation equilibrium inevitably toward the cycloallyl complex. The first hydrofluorination step of 3‐hexyne by HF to give the fluoroalkene is computed to be exergonic by −21.8 kcal/mol, and a second hydrofluorination step to give the *gem*‐difluoroalkane is exergonic by −17.5 kcal/mol. Indeed, it was observed that with extended time, hydrofluorination products are generated. Structural and electronic details of all Pt(II) intermediates and transition states bearing the ligands from dppm (**a**) to dppb (**f**) are given in Table S2 in the Supporting Information.

The bite angles of the ligands (from top to bottom in Table 1) show that the activation free energy for metallacycle formation, proceeding either *via* a [2+1] cycloaddition or a C–C coupling transition state, is sensitive to the structural and electronic properties of the coordinating bidentate phosphine ligands. For the series of diphosphine ligands, the activation free energy of metallacycle formation increases overall with increasing bite angle, from dppm (**a**) to dppp (**e**), which is in accordance with the experimental observations.

**TABLE 1:** Computed activation free energies (in kcal·mol^−^^1^ at 298 K, at (ωB97M‐V/def2‐TZVP//BP86‐D3(BJ)/def2‐TZVP+COSMO‐RS (1,2‐dichloroethane) level) for metallacycle formation with different bidentate phosphine ligands.

	Ligand (L)	Bite angle [^0^]	$\Delta G_{[2+1]\mathrm{cyc}.\;\mathrm{add}.}^{‡}$	$\Delta G_{C-C\;\mathrm{coupl}.}^{‡}$
1	dppm (**a**)	74.7	27.4	28.3
2	dppbz (**b**)	84.5	27.8	29.9
3	dppe (**c**)	87.6	28.2	29.8
4	PCNP (**d**)	85.2	27.0	27.8
5	dppp (**e**)	91.6	30.1	35.8
6	dppb (**f**)	102.6	29.5	33.9

The activation barrier depends to the largest part on ligand bite angle, attributable to steric strain, particularly for ligands with larger bite angles such as dppp (**e**) and dppb (**f**). This strain arises from substrate rotation at the Pt centre prior to reaching the transition state. For example, in the case of the dppp ligand (**e**), the P–Pt–P bite angle is reduced by 2.5° and 4.0° relative to the bis(alkyne) intermediate (**Int‐0**) during the C–C coupling, and [2+1] cycloaddition transition states **TS‐1**, respectively. In contrast, for ligands with smaller bite angles, such as dppm (**a**), the decrease in the P–Pt–P angle is less than 1°.

Despite having a bite angle similar to that of dppe (**c**), the PCNP (**d**) ligand exhibits a lower activation free energy for metallacycle formation via the [2+1] cycloaddition pathway. This observation suggests that, in addition to steric effects, the electronic properties of the coordinating ligand also play an important role in controlling metallacycle formation. Consistent with this interpretation, NPA analysis of the [2+1] cycloaddition transition state coordinated by PCNP reveals an increase in electron density at the Pt centre (NPA charge on Pt = 0.37) relative to the bis(alkyne) intermediate **Int‐0** (NPA charge on Pt = 0.40).

Furthermore, the decrease in the activation free energy of metallacycle formation from dppp (**e**) to dppb (**f**) indicates that the relationship between ligand bite angle and reactivity is not strictly linear. The differences of 1.2 and 2 kcal·mol^−^^1^ in the activation free energy barriers for the [2+1] cycloaddition and C–C coupling transition states, respectively, arise from slightly larger charge separation at the bonding carbon atoms in the transition states involving the dppb (**f**) ligand compared to dppp (**e**). Detailed NPA charges for all optimised intermediates and transition states are provided in Table S2.

Based on the relative activation free energies of the competing transition states, metallacycle complexes are preferentially formed via the [2+1] cycloaddition pathway rather than via C–C coupling. This kinetic preference becomes more pronounced for ligands with larger bite angles, such as dppp (**e**) and dppb (**f**), and can be attributed to increased steric strain imposed on the coordinated ligand framework during substrate rotation, which becomes more substantial when rotating *both* alkyne ligands simultaneously. In spite of the likely overestimated barriers (see above), the DFT calculations overall reproduce the experimentally observed trend for cycloallyl complex formation successfully: dppm ∼ PCNP > dppbz > dppe > dppb > dppp [38].

**SCHEME 2:**
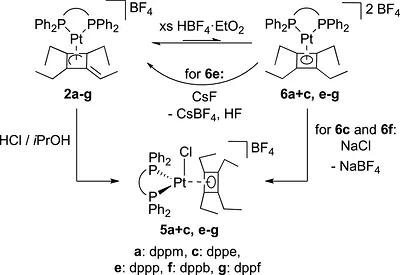
Formation of the cationic and dicationic Pt(II) cyclobutadiene complexes [Pt(Cl)(*η* ^4^‐(C_2_H_5_ *C*)_4_)(L)][BF_4_] (**5a+c**, **5e‐g**) and [Pt(*η* ^4^‐(C_2_H_5_ *C*)_4_)(L)][BF_4_]_2_ (**6a+c**, **6e‐g**).

Next, the complexes **2a**+**c** and **2e**‐**g** were treated with a solution of HCl in *i*PrOH to yield cationic *η* ^4^‐cyclobutadiene Pt(II) complexes [Pt(Cl)(*η* ^4^‐(C_2_H_5_ *C*)_4_)(L)][BF_4_] (**5a**+**c**, **5e**‐**g**), which bear an additional chlorido ligand (Scheme 2). The ^31^P{^1^H} NMR spectra of **5a**+**c**, **5e**‐**g** show a singlet with ^195^Pt satellites and ^1^ *J*_P,Pt_ coupling constants within the range of 2801–3402 Hz, which is in accordance with literature‐reported values for these Pt(II) cyclobutadiene complexes. Due to the limited stability of complexes **5a**+**c** and **5e**‐**g**, the formation of dichlorido (**1a**+**c**, **1e**‐**g**) and cycloallyl analogues (**2a**+**c** and **2e**‐**g**) was observed over time, accompanied by a diminishing signal intensity for **5**. For **2d** with PCNP as ligand, an immediate formation of the dichlorido complex **1d** was observed and the system is therefore not considered in the following discussion. The ^1^H NMR spectra revealed for each complex a quartet of triplets with ^195^Pt satellites (δ ≈ 1.80 ppm) for the CH_2_ unit of the cyclobutadiene ligand, which collapses into a quartet with ^195^Pt satellites in the corresponding ^1^H{^31^P} NMR spectrum. ^1^H,^1^H COSY NMR experiments showed a cross peak to a triplet signal at δ ≈ 1.00 ppm, which can be assigned to the neighbouring methyl group. For [Pt(Cl)(*η* ^4^‐(C_2_H_5_ *C*)_4_)(dppe)][BF_4_] (**5c**) the ^13^C{^1^H} NMR resonance for the quaternary cyclobutadiene carbon was observed at δ = 104.4 ppm as a triplet signal with ^195^Pt satellites due to coupling to the metal centre and the phosphorous atoms (^1^ *J*_C,Pt_ = 96 Hz, ^2^ *J*_C,P_ = 4 Hz). The spectrum further revealed a triplet with ^195^Pt satellites for the CH_2_ moiety at δ = 18.1 ppm (^2^ *J*_C,Pt_ = 50 Hz, ^2^ *J*_C,P_ = 2 Hz). Complex **5c** was further characterised by ESI‐MS, revealing a molecular ion peak for [M]^+^ at *m*/*z* 820.2 (calculated: *m*/*z* = 820.26). During the reaction, traces of the trimerization product hexaethylbenzene were identified by NMR spectroscopy and GC‐MS methods.

**FIGURE 5:**
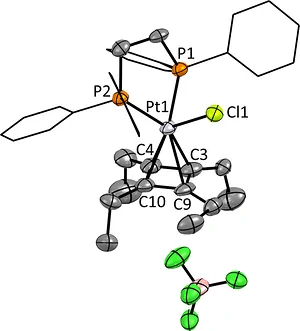
Structure of [Pt(Cl)(*η* ^4^‐(C_2_H_5_ *C*)_4_)(dppe)][BF_4_]·CH_2_Cl_2_ (**5c**·CH_2_Cl_2_) in the solid state. Phenyl substituents are depicted as wireframes, and thermal ellipsoids are depicted at 50% probability. Solvent molecule (CH_2_Cl_2_), hydrogen atoms, and the second molecule in the asymmetric unit are omitted for clarity. Selected bond lengths [Å] and angles [°]: Pt1–Cl1 2.465(2), Pt1–C3 2.234(9), Pt1–C4 2.105(9), Pt1–C9 2.279(9), Pt1–C10 2.162(9), C3–C4 1.462(13), C3–C9 1.418(13), C4–C10 1.477(13), C9–C10 1.492(14); P1–Pt1–P2 86.20(8), Cl1–Pt1–P1 88.49(8), Cl1–Pt1–P2 88.30(8).

The structure of **5c**·CH_2_Cl_2_ was additionally verified by x‐ray crystallography (Figure 5). The compound crystallizes in the monoclinic space group *P*2_1_/*n*. The asymmetric unit contains two molecules, of which one revealed a disorder of the two ethyl groups of the cyclobutadiene ligand, as well as for the BF_4_^−^ counter anion. The coordination arrangement of **5c**·CH_2_Cl_2_ is best described as slightly distorted square pyramidal around the platinum centre with the chloride ligand occupying the apex, as indicated by a geometry index of τ_4_ = 0.15.^[40]^ The structure of **5c**·CH_2_Cl_2_ shows C–C bond lengths between 1.418(13) Å and 1.492(14) Å for the ring carbon atoms of the cyclobutadiene ligand. The P1–Pt1–P2 bond angle exhibits a bite angle of 86.20(8) °.

Remarkably, when treating the cycloallyl complexes **2a**+**c, 2e**‐**g** with an excess of HBF_4_·Et_2_O the corresponding dicationic Pt(II) complexes [Pt(*η* ^4^‐(C_2_H_5_ *C*)_4_)(L)][BF_4_]_2_ (**6a**+**c, 6e**‐**g**) were formed (Scheme 2). The presence of the Pt(II) cycloallyl complex **2** was also observed by NMR spectroscopy. The increase of its signal intensity over time at room temperature suggests an equilibrium between complexes **2a**+**c, 2e**‐**g** and complexes **6a**+**c, 6e**‐**g** in the presence of HBF_4_·Et_2_O. When forming **6a** and **6c**, their corresponding aqua complexes [Pt(H_2_O)_2_(L)][BF_4_]_2_ (L = dppm, dppe) are additionally formed in various amounts, resulting from traces of water in HBF_4_·Et_2_O.

Although the syntheses of neutral and cationic mono‐ and dinuclear Pt(II) cyclobutadiene complexes have been reported in literature [17, 20, 24, 39], the formation of a dicationic Pt(II) *η* ^4^‐cyclobutadiene complex bearing phosphine ligands has only been proposed as an off‐cycle intermediate yielding a Pt(II) *η* ^3^‐cycloallyl complex [15]. Recent investigations by Shvydkiy and coworkers have shown that a Pt(II) *η* ^4^‐cyclobutadiene complex can be applied in a catalytic hydrosilylation reaction of alkynes and alkenes [23].

**FIGURE 6:**
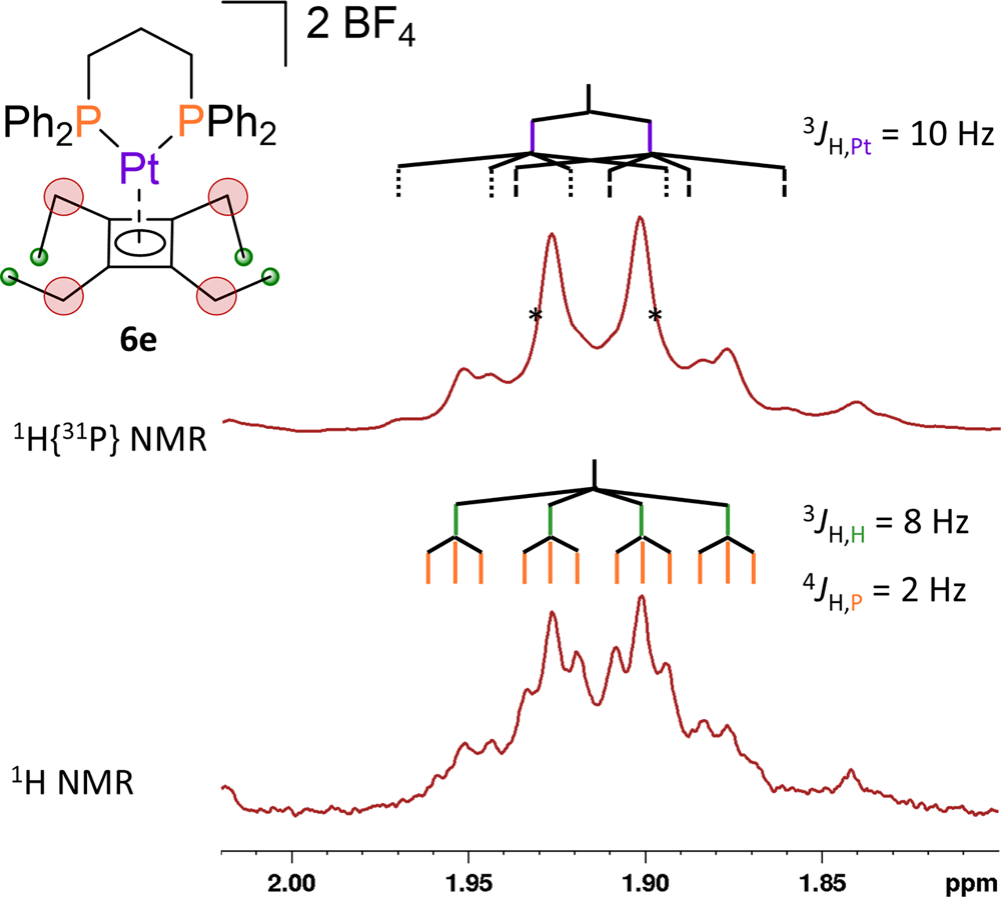
Excerpt of the ^1^H{^31^P} (top) and ^1^H (bottom) NMR spectrum of [Pt(*η* ^4^‐(C_2_H_5_ *C*)_4_)(dppp)][BF_4_]_2_ (**6e**) showing the signals of the CH_2_ moiety (red circle) from the cyclobutadiene ligand. * depict ^195^Pt satellites.

The ^31^P{^1^H} NMR spectra of **6a**+**c** and **6e**‐**g** revealed each a singlet with ^195^Pt satellites and ^1^ *J*_P,Pt_ coupling constants within the range of 3677–4141 Hz. The cyclobutadiene ligand was characterized by ^1^H NMR spectra showing signals for the CH_2_ moiety at around δ ≈ 2 ppm. The signal exhibits a quartet of a triplet with satellites due to coupling to the adjacent methyl group as well as both phosphorous nuclei (^3^ *J*_H,H_ = 8 Hz, ^4^ *J*_H,P_ = 2 Hz). ^195^Pt satellites were determined showing a coupling constant of ^3^ *J*_H,Pt_ = 10 Hz obscured by the main signal for **6e** (Figure 6). ^1^H,^1^H COSY NMR experiments revealed a cross peak of the quartet of triplet signal to a triplet signal at around δ ≈ 1 ppm which can be assigned to the methyl groups of the cyclobutadiene ligand. A summary of selected NMR data of the complexes **5a**+**c, 5e**‐**g** and **6a**+**c, 6e**‐**g** is given in Table 2.

**TABLE 2:** Selected ^31^P{^1^H} and ^1^H NMR data of the cyclobutadiene ligand (δ in ppm, *J* in Hz) for complexes **5a+c**, **5e‐g** and **6a+c**, **6e‐g**.

		^31^P{^1^H} NMR		^1^H NMR	
Complex	L	δ (P)	^1^ *J*_P,Pt_	δ (CH_2_)	δ (CH_3_)
**5a**	dppm	−23.9	2801	2.23	1.17
**6a**		3.0	3270	2.31	1.05
**5c**	dppe	28.3	3261	2.03	1.08
**6c**		57.7	3706	2.17	0.98
**5e**	dppp	−11.0	3225	1.86	1.01
**6e**		12.6	3677	1.96	1.08
**5f**	dppb	−1.0	3402	1.62	1.01
**6f**		24.7	3958	1.73	0.99
**5g**	dppf	−11.0	3224	1.86	1.02
**6g**		33.3	4141	1.70	1.05

The complexes [Pt(*η* ^4^‐(C_2_H_5_ *C*)_4_)(dppe)][BF_4_]_2_ (**6c**) and [Pt(*η* ^4^‐(C_2_H_5_ *C*)_4_)(dppp)][BF_4_]_2_ (**6e**) were chosen exemplarily for testing their reactivity toward an excess of NaCl. Upon removal of NaBF_4_ by extraction, the formation of the cationic chlorido cyclobutadiene complexes [Pt(Cl)(*η* ^4^‐(C_2_H_5_ *C*)_4_)(dppe)][BF_4_] (**5c**) and [Pt(Cl)(*η* ^4^‐(C_2_H_5_ *C*)_4_)(dppp)][BF_4_] (**5e**) was observed (Scheme 2). A mixture of the two dicationic cyclobutadiene complexes derived from cycloallyl complexes **3c** and **2e** in HBF_4_·Et_2_O was then treated with NaCl. The reaction led solely to the cationic cyclobutadiene chlorido complexes **5f** and [Pt(Cl)(*η* ^4^‐(C_3_H_7_ *C*)_4_)(dppe)][BF_4_]. The latter is the analogue of **5f** but with a propyl substituent at the cyclobutadiene moiety. This suggests that no intermolecular exchange of cyclobutadiene ligands occurs. In contrast, treatment of **6e** with CsF leads to the generation of Pt(II) *η* ^3^‐cycloallyl complex **2e**, since the fluoride acts in this case as a base and not as a nucleophile (Scheme 2).

## 3 Conclusions

This work provides routes to access Pt(II) *η* ^3^‐cycloallyl and cyclobutadiene complexes with various diphosphine ligands. Experimental investigations of their formation revealed a pronounced influence of the ligand bite angle on the formation rates. DFT calculations confirmed the experimental observations and provided mechanistic insights into pathways originating from a bis(alkyne) intermediate. In the cyclization pathway, the BF_4_^−^ anion may act as a base, giving Pt(II) *η* ^3^‐cycloallyl complexes **2a‐g**. Treatment of complexes **2a‐g** with different Br*ø*nsted acids enables the formation of cationic and dicationic Pt(II) *η* ^4^‐cyclobutadiene half‐sandwich complexes [Pt(Cl)(*η* ^4^‐(C_2_H_5_ *C*)_4_)(L)][BF_4_] (**5a+c, 5e‐g**) and [Pt(*η* ^4^‐(C_2_H_5_ *C*)_4_)(L)][BF_4_]_2_ (**6a+c, 6e‐g**). Notably, the identity of the Br*ø*nsted acid anion (BF_4_^−^, Cl^−^, F^−^) controls the interconversion between the Pt(II) *η* ^3^‐cycloallyl complexes **2a‐g** and the *η* ^4^‐cyclobutadiene complexes **5a+c**, **5e‐g** and **6a+c**, **6e‐g**.

## Author Contributions

OH and YEHS carried out the experiments and collected analytical data. FJ and MT performed DFT calculations. OH and FJ wrote the original draft. LMR performed NMR monitoring measurements. OH did the single crystal X‐ray diffraction analyses. TB and OH conceived the project, and TB provided guidance throughout the study. MK and TB supervised the work. All authors contributed to the manuscript and edited it.

## Conflicts of Interest

The authors declare no conflicts of interest.

## Supporting information



The authors have cited additional references within the Supporting Information [12, 41, 42, 43, 44, 45, 46, 47, 48, 49, 50, 51, 52, 53, 54, 55, 56, 57, 58, 59, 60, 61, 62, 63, 64, 65, 66].
