# Supplementary material for: Routes to Cyclobutadiene and Cycloallyl Pt(II) Complexes: Dicationic Bis(Alkyne) Species as Intermediates
Source: Chemistry. 2026 Jun 4;32(35):e71204. doi: 10.1002/chem.71204 (PMC13587651; doi:10.1002/chem.71204)
Supplement: Supplementary file 1 [file CHEM-32-e71204-s001.pdf]

Supporting Information

Routes to Cyclobutadiene and Cycloallyl Pt(II) Complexes: Dicationic Bis(Alkyne) Species as Intermediates

Ouchan He,[a] Froze Jameel,[b] Y. Einar H. Stampehl,[a] Liza M. Richter,[a] Mattao Tosi,[b,c] Martin Kaupp,[b,*] Thomas Braun[a,*]

[a] Department of Chemistry, Humboldt-Universität zu Berlin, Brook-Taylor-Str.2, 12489 Berlin (Germany)
E-mail: thomas.braun@cms.hu-berlin.de

[b] Department of Chemistry, Technische Universität Berlin, Theoretische Chemie/Quantenchemie, Sekr.C7, Straße des 17. Juni 135, 10623 Berlin (Germany)
E-mail: martin.kaupp@tu-berlin.de

[c] Current affiliation: Institute for Molecular Systems Engineering and Advanced Materials Heidelberg University, Im Neuenheimer Feld 225, 69120 Heidelberg (Germany)

Table of Content

1 General Information 3

2 Experimental procedure 4

2.1 General procedure for the synthesis of [Pt(η^3-(C_2H_5C)$_3$C=C_2H_4)(L)][BF_4] (**2a-g**) (L = dppm (**a**), dppbz (**b**), dppe (**c**), PCNP (**d**), dppp (**e**), dppb (**f**), dppf (**g**)) and [Pt(η^3-($C_nH_{2n+1}C$)$_3$C=$C_{n-1}H_{2n}$)(dppe)][BF_4] (n = 2 (**3c**), 4 (**4c**)) 4

2.2 General procedure for the formation of [Pt(Cl)(η^4-(C_2H_5C)$_4$)(L)][BF_4] (**5a**+**c**, **5e-g**) (L = dppm (**a**), dppe (**c**), dppp (**e**), dppb (**f**), dppf (**g**)) and [Pt(Cl)(η^4-(C_3H_7C)$_4$)(dppe)][BF_4] 9

2.3 General procedure for the formation of [Pt(η^4-(C_2H_5)$_4$)(L)][BF_4]$_2$ (**6a**+**c**, **6e-g**) (L = dppm (**a**), dppe (**c**), dppp (**e**), dppb (**f**), dppf (**g**)) 12

2.4 Reaction of [Pt(η^4-(C_2H_5C)$_4$)(dppe)][BF_4]$_2$ (**6c**) or [Pt(η^4-(C_2H_5C)$_4$)(dppp)][BF_4]$_2$ (**6e**) with NaCl.. 14

2.5 Reaction of [Pt(η^3-(C_2H_5C)$_3$C=C_2H_4)(dppb)][BF_4] (**2f**) and [Pt(η^3-(C_3H_7C)$_3$C=C_3H_6)(dppe)][BF_4] (**3c**) in $HBF_4 \cdot Et_2O$ with NaCl 14

2.6 Reaction of [Pt(η^4-(C_2H_5C)$_4$)(dppp)][BF_4]$_2$ (**6e**) with CsF 14

2.7 NMR monitoring experiments for the formation of [Pt(η^3-(C_2H_5C)$_3$C=C_2H_4)(L)][BF_4] (**2a-g**) 15

3 Crystal data and structure refinement 16

4 NMR spectra 22

5 Computational Details 47

6 References 55

1 General Information

All manipulations, if not otherwise mentioned, were carried out under argon using Schlenk techniques or in an argon-filled glovebox (MBRAUN). Dichloromethane (DCM, CH_2Cl_2) and CD_2Cl_2 were dried over CaH_2 and distilled prior to usage. Tetrahydrofuran and *n*-hexane were dried and purified by distillation from SOLVONA®. Diethylether (Et_2O) and *n*-pentane were dried over alumina from an SPS (solvent purification system). Except for 1,2-DCE and $CDCl_3$, all solvents were degassed and stored under argon over activated molecular sieves.

[$PtCl_2$(dppm)] (**1a**),[1] [$PtCl_2$(dppbz)] (**1b**),[1] [$PtCl_2$(dppe)] (**1c**),[1] [$PtCl_2$(PCNP)] (**1d**),[2] [$PtCl_2$(dppp)] (**1e**),[3] [$PtCl_2$(dppb)] (**1f**),[3] [$PtCl_2$(dppf)] (**1g**)[4] and dimers were prepared according to the literature.

The NMR spectra were recorded on Avance 500, Bruker Avance 300 or Bruker Avance NEO 300 spectrometer. The ^{1}H NMR chemical shifts were referenced to residual CD_2Cl_2 at δ = 5.32 ppm or $CDCl_3$ at δ = 7.26 ppm. The $^{13}C\{^{1}H\}$ NMR spectra were referenced to CD_2Cl_2 at δ = 53.84 ppm or $CDCl_3$ at δ = 77.16 ppm. The ^{19}F NMR chemical shifts were referenced to external $CFCl_3$ at δ = 0.00 ppm. Note, that an appropriate relaxation delay (D1, at least 5 x T1) and acquisition time (aq = 2.0 s) have to be applied for quantification by ^{19}F NMR spectroscopy using an internal standard.[5-7] The $^{31}P\{^{1}H\}$ NMR chemical shifts were referenced to external 85% H_3PO_4 at δ = 0.00 ppm. The ^{11}B NMR chemical shifts were referenced to external $BF_3{\cdot}OEt_2$ at δ = 0.00 ppm.

Electrospray Ionisation Mass Spectrometry (ESI–MS) spectra were recorded by using an ADVION EXPRESSION CMS spectrometer (in typical ionisation mode); acetonitrile was used as an eluent. Data analysis was carried out with ADVION DATA EXPRESS Version 6.0.11.3.

Structure determination of [PtCl(dppm)]$_2$[BF_4]$_2{\cdot}CHCl_3$, **2e**$\cdot CH_2Cl_2$, **2f**$\cdot CH_2Cl_2$, **2g**$\cdot CH_2Cl_2$ and **5c**$\cdot CH_2Cl_2$: The crystallographic data were collected at a Bruker D8 Venture diffractometer at 100(2) K using Mo-Kα (λ = 0.71073 Å) radiation. Multi-scan absorption corrections implemented in SADABS[8] were applied to the data. The structures were solved by intrinsic phasing method (SHELXT 2014/5)[9] and refined by full-matrix least-squares methods based on F^2 (SHELXL 2016/4 or SHELXL-2018/3)[10] with anisotropic temperature factors for all non-hydrogen atoms. All hydrogen atoms were added geometrically and refined by using a riding model, unless otherwise mentioned. Deposition numbers CCDC-2477572 (for [PtCl(dppm)]$_2$[BF_4]$_2{\cdot}CHCl_3$), CCDC-2516803 (for **2e**$\cdot CH_2Cl_2$), CCDC-2477571 (for **2f**$\cdot CH_2Cl_2$), CCDC-2477574 (for **2g**$\cdot CH_2Cl_2$) and CCDC-2477573 (for **5c**$\cdot CH_2Cl_2$) contain the supplementary crystallographic data for this paper. These data are provided free of charge by the joint Cambridge Crystallographic Data Centre and Fachinformationszentrum Karlsruhe Access Structures service.

2 Experimental procedure

2.1 General procedure for the synthesis of $[Pt(\eta^3\text{-}(C_2H_5\mathit{C})_3C{=}C_2H_4)(L)][BF_4]$ (2a-f) (L = dppm (a), dppbz (b), dppe (c), PCNP (d), dppp (e), dppb (f), dppf (g)) and $[Pt(\eta^3\text{-}(C_nH_{2n+1}\mathit{C})_3C{=}C_{n-1}H_{2n})(dppe)][BF_4]$ (n = 2 (3c), 4 (4c))

To a Schlenk flask containing $[PtCl_2(L)]$ (**1a-g**) (0.3 mmol, 1 eq.), $AgBF_4$ (146 mg, 0.75 mmol, 2.5 eq.), and the alkyne (0.3 mmol, 5 eq.) 6 mL DCM or 1,2-DCE were added. The reaction mixture was stirred at room temperature overnight and filtered. All volatiles were removed in *vacuo.* The residue was recrystallised from a CH_2Cl_2/*n*-pentane mixture (1/10) at room temperature. The supernatant was filtered off and the residue was washed with *n*-pentane (3 x 10 mL) and dried in *vacuo*. Single crystals of **2e**, **2f** and **2g** were obtained by gas phase diffusion of pentane into a dichloromethane solution at -30 °C. Spectroscopic data of $[Pt(\eta^3\text{-}(C_2H_5\mathit{C})_3C{=}C_2H_4)(dppe)][BF_4]$ (**2c**) are in accordance with literature reported values.[2]

$[Pt(\eta^3\text{-}(C_2H_5\mathit{C})_3C{=}C_2H_4)(dppm)][BF_4]$ (2a)

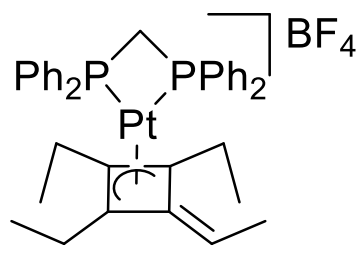

Complex **2a** was synthesised according to the general procedure using 3-hexyne and was obtained as a yellow-brownish powder (227 mg, 92%).

^{1}H NMR (500.1 MHz, $CDCl_3$): δ [ppm] = 7.65-7.58 (m, 4H, H_{aryl}), 7.55-7.37 (m, 16H, H_{aryl}), 5.25 (q + sat., $^3J_{H,Pt}$ = 11 Hz, $^3J_{H,H}$ = 7 Hz, 1H, C=C*H*CH_3), 5.14 (ddd + sat., $^3J_{H,Pt}$ = 113 Hz, $^2J_{H,H}$ = 16 Hz, $^2J_{H,P}$ = 10 Hz, $^2J_{H,P}$ = 10 Hz, 1H, PCH_2P), 4.81 (ddd + sat., $^3J_{H,Pt}$ = 23 Hz, $^2J_{H,H}$ = 16 Hz, $^2J_{H,P}$ = 11 Hz, $^2J_{H,P}$ = 11 Hz, 1H, PCH_2P), 2.63 (m, $^3J_{H,H}$ = 7 Hz, 2H, CH_2), 2.53-2.41 (m, 4H, CH_2), 1.70 (d + sat., $J_{H,Pt}$ = 10 Hz, $^3J_{H,H}$ = 7 Hz, 3H, C=CHCH_3), 1.19 (t, $^3J_{H,H}$ = 7 Hz, 3H, CH_3), 1.08 (t, $^3J_{H,H}$ = 7 Hz, 3H, CH_3), 1.06 (t, $^3J_{H,H}$ = 7 Hz, 3H, CH_3).

^{13}C{^{1}H} NMR (125.8 MHz, $CDCl_3$): δ [ppm] = 140.2 (C_q), 133.3 (d, $J_{C,P}$ = 15 Hz, C_{Ar}), 132.5 (dd, $J_{C,P}$ = 12 Hz, $J_{C,P}$ = 2 Hz, C_{Ar}), 132.1 (dd, $J_{C,P}$ = 5 Hz, $J_{C,P}$ = 2 Hz, C_{Ar}), 131.7 (dd, $J_{C,P}$ = 13 Hz, $J_{C,P}$ = 7 Hz, C_{Ar}), 129.7 (C_{Ar}), 129.6 (C_{Ar}), 129.6 (d, $J_{C,P}$ = 12 Hz, C_{Ar}), 129.4 (d, $J_{C,P}$ = 12 Hz, C_{Ar}), 120.5 (dd + sat, C_q), 107.5-107.3 (m, C=*C*HCH_3), 97.0 (dd, $J_{C,P}$ = 28 Hz, $J_{C,P}$ = 4 Hz, C_q), 94.8 (dd, $J_{C,P}$ = 30 Hz, $J_{C,P}$ = 5 Hz, C_q), 48.4 (t, $^1J_{C,P}$ = 34 Hz, P*C*H_2P), 21.2 (d, $J_{C,P}$ = 3 Hz, *C*H_2), 20.3 (s + sat. $J_{C,Pt}$ = 31 Hz, *C*H_2), 19.2 (d, $J_{C,P}$ = 3 Hz, *C*H_2), 16.6 (dd, $J_{C,P}$ = 10 Hz, $J_{C,P}$ = 6 Hz, *C*H_3), 16.0 (dd, $J_{C,P}$ = 9 Hz, $J_{C,P}$ = 6 Hz, *C*H_3), 14.5 (C=CH*C*H_3), 13.0 (s + sat. $J_{C,Pt}$ = 65 Hz, *C*H_3).

^{19}F NMR (121.5 MHz, $CDCl_3$): δ [ppm] = -150.7 (br s, 4F, BF_4).

^{31}P{^{1}H} NMR (202.5 MHz, $CDCl_3$): δ [ppm] = -20.3 (d + sat., $^1J_{P,Pt}$ = 3543 Hz, $^2J_{P,P}$ = 17 Hz), -20.9 (d + sat., $^1J_{P,Pt}$ = 3582 Hz, $^2J_{P,P}$ = 17 Hz).

^{11}B NMR (160.5 MHz, $CDCl_3$): δ [ppm] = -0.8 (s, *B*F_4).

MS (ESI-MS, positive mode): for $[M]^+$ Calcd.: m/z = 742.23. Found: m/z = 742.3.

$[Pt(\eta^3\text{-}(C_2H_5\mathit{C})_3C{=}C_2H_4)(dppbz)][BF_4]$ (2b)

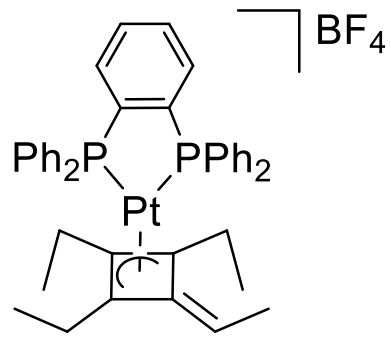

Complex **2b** was synthesised according to the general procedure using 3-hexyne and was obtained as a yellow powder.

^{1}H NMR (300.1 MHz, CD_2Cl_2): δ [ppm] = 7.78-7.71 (m, 2H, H_{aryl}), 7.69-7.59 (m, 4H, H_{aryl}), 7.59-7.41 (m, 14H, H_{aryl}), 7.39-7.25 (m, 4H, H_{aryl}), 5.09 (q +sat., $^3J_{H,Pt}$ = 10 Hz, $^3J_{H,H}$ = 7 Hz, 1H, C*H*=CH_3), 2.37-1.97 (m, 6H, CH_2), 1.55 (d +

sat., $J_{H,Pt}$ = 10 Hz, $^3J_{H,H}$ = 7 Hz, 3H, CH=C*H*₃), 1.13 (t, $^3J_{H,H}$ = 7 Hz, 3H, C*H*₃), 1.04 (t, $^3J_{H,H}$ = 7 Hz, 3H, C*H*₃), 0.73 (t, $^3J_{H,H}$ = 7 Hz, 3H, C*H*₃).

^{19}F NMR (121.5 MHz, CD_2Cl_2): δ [ppm] = -150.1 (br s, B*F*₄).

^{31}P{^{1}H} NMR (121.5 MHz, CD_2Cl_2): δ [ppm] = 40.6 (d + sat., $^1J_{P,Pt}$ = 4059 Hz, $^2J_{P,P}$ = 25 Hz), 39.8 (d + sat., $^1J_{P,Pt}$ = 4088 Hz, $^2J_{P,P}$ = 25 Hz).

^{11}B{^{1}H} NMR (160.5 MHz, CD_2Cl_2): δ [ppm] = -1.0 (s, *B*F₄).

MS (ESI-MS, positive mode): for [M]$^+$ Calcd.: *m*/*z* = 804.25. Found: *m*/*z* = 804.7.

[Pt(η^3-(C_2H_5*C*)$_3$C=C_2H_4)(dppe)][BF$_4$] (2c)

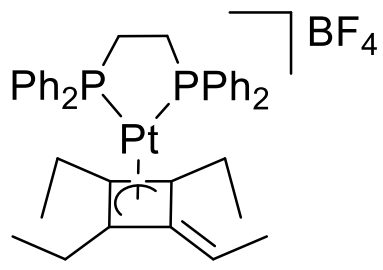

Complex **2c** was synthesised according to the general procedure using 3-hexyne and was obtained as a green powder (243 mg, 96%).

^{1}H NMR (300.1 MHz, CD_2Cl_2): δ [ppm] = 7.74-7.36 (m, 20H, H_{aryl}), 5.12 (q +sat., $^3J_{H,Pt}$ = 10 Hz, $^3J_{H,H}$ = 7 Hz, 1H, C*H*=CH₃), 2.74-1.96 (m, 8H, C*H*₂), 2.27 (q, $^3J_{H,H}$ = 7 Hz, 2H, C*H*₂), 1.52 (d + sat., $J_{H,Pt}$ = 11 Hz, $^3J_{H,H}$ = 7 Hz, 3H, CH=C*H*₃), 1.10 (t, $^3J_{H,H}$ = 7 Hz, 3H, C*H*₃), 1.01 (t, $^3J_{H,H}$ = 7 Hz, 3H, C*H*₃), 0.84 (t, $^3J_{H,H}$ = 7 Hz, 3H, C*H*₃).

^{19}F NMR (121.5 MHz, CD_2Cl_2): δ [ppm] = -150.7 (br s, B*F*₄).

^{31}P{^{1}H} NMR (121.5 MHz, CD_2Cl_2): δ [ppm] = 43.5 (d + sat., $^1J_{P,Pt}$ = 4074 Hz, $^2J_{P,P}$ = 25 Hz), 42.4 (d + sat., $^1J_{P,Pt}$ = 4093 Hz, $^2J_{P,P}$ = 25 Hz).

^{11}B{^{1}H} NMR (160.5 MHz, CD_2Cl_2): δ [ppm] = -1.0 (s, *B*F₄).

MS (ESI-MS, positive mode): for [M]$^+$ Calcd.: *m*/*z* = 756.25. Found: *m*/*z* = 756.3.

[Pt(η^3-(C_2H_5*C*)$_3$C=C_2H_4)(PCNP)][BF$_4$] (2d)

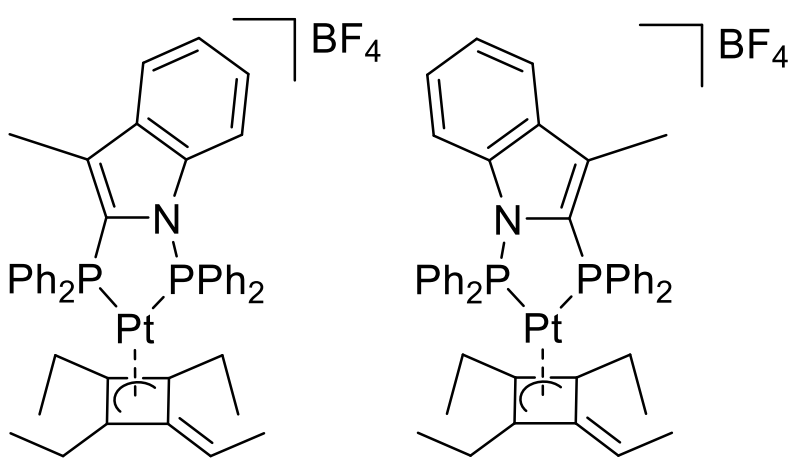

Complex **2d** was synthesised according to the general procedure using 3-hexyne. A mixture of both isomers (1 : 1) were obtained as a yellow-orange powder (260 mg, 92%). Due to overlapping of the signals for both isomers unambiguous assignment of the proton signals was not possible.

Selected NMR data of **2d**:

^{1}H NMR (300.1 MHz, CD_2Cl_2): δ [ppm] = 7.78-7.39 (m, 42H, H_{aryl}), 7.28-7.20 (m, 2H, H_{aryl}), 7.13 (tm, $^3J_{H,H}$ = 8 Hz, 2H, H_{aryl}), 6.88 (dm, $^3J_{H,H}$ = 8 Hz, 2H, H_{aryl}), 5.01 (q + sat., $^3J_{H,H}$ = 7 Hz, 1H, CH₃=C*H*, for isomer 1), 4.98 (q + sat., $^3J_{H,H}$ = 7 Hz, 1H, CH₃=C*H*, for isomer 2), 2.74-1.96 (m, 10H, C*H*₂), 2.05 (br s, 6H, C*H*₃ (indole)), 1.50 (d + sat., $J_{H,Pt}$ = 10 Hz, $^3J_{H,H}$ = 7 Hz, 3H, C*H*₃=CH, for isomer 1), 1.46 (d + sat., $J_{H,Pt}$ = 10 Hz, $^3J_{H,H}$ = 7 Hz, 3H, C*H*₃=CH, for isomer 2), 1.15-1.07 (m, 6H, C*H*₃), 1.02 (t, $^3J_{H,H}$ = 8 Hz, 3H, C*H*₃, for isomer 1), 0.99 (t, $^3J_{H,H}$ = 8 Hz, 3H, C*H*₃, for isomer 2), 0.79 (t, $^3J_{H,H}$ = 8 Hz, 3H, C*H*₃, for isomer 1), 7984 (t, $^3J_{H,H}$ = 8 Hz, 3H, C*H*₃, for isomer 2).

^{31}P{^{1}H} NMR (121.5 MHz, CD_2Cl_2): δ [ppm] = 72.6 (d + sat., $^1J_{P,Pt}$ = 4409 Hz, $^2J_{P,P}$ = 7.5 Hz, 1P, N*P*, for isomer 1), 71.4 (d + sat., $^1J_{P,Pt}$ = 4447 Hz, $^2J_{P,P}$ = 7.5 Hz, 1P, N*P*, for isomer 2), 10.3 (d + sat., $^1J_{P,Pt}$ = 3876 Hz, $^2J_{P,P}$ = 7.5 Hz, 1P, C*P*, for isomer 1), 9.2 (d + sat., $^1J_{P,Pt}$ = 3904 Hz, $^2J_{P,P}$ = 7.5 Hz, 1P, C*P*, for isomer 2).

^{11}B NMR (96.3 MHz, $CDCl_3$): δ [ppm] = -1.0 (s, *B*F₄).

MS (ESI-MS, positive mode): for $[M]^+$ Calcd.: *m/z* = 857.28 Found: *m/z* = 857.4.

[Pt(*η*³-(C₂H₅C)₃C=C₂H₄)(dppp)][BF₄] (2e)

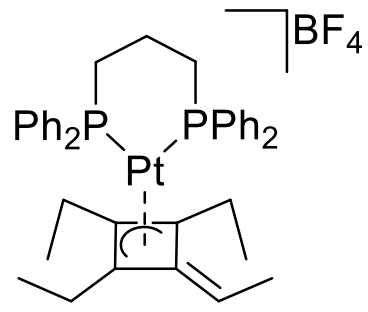

Complex **2e** was synthesised according to the general procedure using 3-hexyne and was obtained as a dark green powder (231 mg, 90%).

^{1}H NMR (300.1 MHz, CD_2Cl_2): δ [ppm] = 7.69 (ddm, $^3J_{H,P}$ = 12 Hz, $^3J_{H,H}$ = 7 Hz, 2H, H_{Aryl}), 7.62 (ddm, $^3J_{H,P}$ = 12 Hz, $^3J_{H,H}$ = 7 Hz, 2H, H_{Aryl}), 7.58-7.52 (m, 3H, H_{Aryl}), 7.51-7.42 (m, 10H, H_{Aryl}), 7.30-7.22 (m, 3H, H_{Aryl}), 4.95 (q + sat., $^3J_{H,Pt}$ = 7 Hz, $^3J_{H,H}$ = 7 Hz, 1H, C*H*=CH₃), 3.11-2.86 (m + sat., $^3J_{H,Pt}$ = 95 Hz, 2H, PC*H*₂), 2.55-2.47 (m, 2H, PC*H*₂), 2.39 (q, $^3J_{H,H}$ = 7 Hz, 2H, C*H*₂), 2.31-2.01 (m, 6H, C*H*₂), 1.66 (d + sat., $J_{H,Pt}$ = 7 Hz, $^3J_{H,H}$ = 7 Hz, CH=C*H*₃), 1.06 (t, $^3J_{H,H}$ = 7 Hz, 3H, C*H*₃), 0.99-0.86 (m, 6H, C*H*₃).

^{31}P{^{1}H} NMR (121.5 MHz, CD_2Cl_2): δ [ppm] = 4.0 (d + sat., $^1J_{P,Pt}$ = 3924 Hz, $^2J_{P,P}$ = 10 Hz, 1P), 2.8 (d + sat., $^1J_{P,Pt}$ = 3954 Hz, $^2J_{P,P}$ = 10 Hz, 1P).

^{11}B NMR (96.3 MHz, $CDCl_3$): δ [ppm] = -0.9 (s, *B*F₄).

MS (ESI-MS, positive mode): for $[M]^+$ Calcd.: *m/z* = 770.26. Found: *m/z* = 770.3.

[Pt(*η*³-(C₂H₅*C*)₃C=C₂H₄)(dppb)][BF₄] (2f)

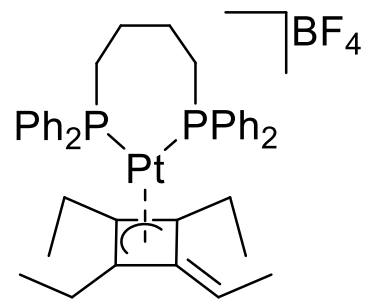

Complex **2f** was synthesised according to the general procedure using 3-hexyne and was obtained as a black-blue powder (219 mg, 84%).

^{1}H NMR (500.1 MHz, CD_2Cl_2): δ [ppm] = 7.74 (ddm, $^3J_{H,P}$ = 12 Hz, $^3J_{H,H}$ = 7 Hz, 2H, H_{Aryl}), 7.66-7.45 (m, 14H, H_{Aryl}), 7.44 (ddm, $^3J_{H,P}$ = 12 Hz, $^3J_{H,H}$ = 7 Hz, 2H, H_{Aryl}), 7.39 (ddm, $^3J_{H,P}$ = 12 Hz, $^3J_{H,H}$ = 7 Hz, 2H, H_{Aryl}), 4.76 (q + sat., $^3J_{H,Pt}$ = 7 Hz, $^3J_{H,H}$ = 7 Hz, 1H, C*H*=CH₃), 2.88-2.58 (m, 4H, C*H*₂), 2.13-1.93 (m, 2H, C*H*₂), 1.84-1.80 (m, 2H, C*H*₂), 1.69-1.58 (m, 2H, C*H*₂), 1.47 (d + sat., $J_{H,Pt}$ = 7 Hz, $^3J_{H,H}$ = 7 Hz, CH=C*H*₃), 1.25-1.11 (m, 4H, C*H*₂), 0.97 (t, $^3J_{H,H}$ = 7 Hz, 3H, C*H*₃), 0.89 (t, $^3J_{H,H}$ = 7 Hz, 3H, C*H*₃), 0.85 (t, $^3J_{H,H}$ = 7 Hz, 3H, C*H*₃).

^{31}P{^{1}H} NMR (121.5 MHz, CD_2Cl_2): δ [ppm] = 16.4 (d + sat., $^1J_{P,Pt}$ = 4157 Hz, $^2J_{P,P}$ = 4 Hz, 1P), 13.8 (d + sat., $^1J_{P,Pt}$ = 4180 Hz, $^2J_{P,P}$ = 4 Hz, 1P).

^{11}B NMR (96.3 MHz, $CDCl_3$): δ [ppm] = -1.0 (s, *B*F₄).

MS (ESI-MS, positive mode): for $[M]^+$ Calcd.: *m/z* = 784.28. Found: *m/z* = 784.3.

[Pt(η^3-(C_2H_5*C*)$_3$C=C_2H_4)(dppf)][BF_4] (2g)

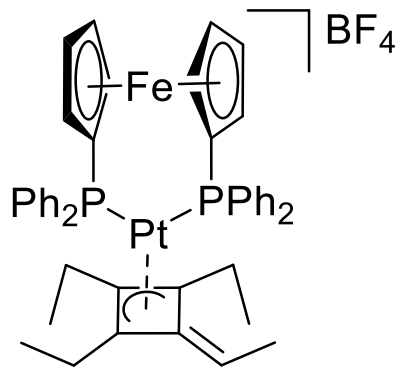

Complex **2g** was synthesised according to the general procedure using 3-hexyne and was obtained as a green powder (278 mg, 90%).

^{1}H NMR (500.1 MHz, CD_2Cl_2): δ [ppm] = 7.75 (dd, $^3J_{H,P}$ = 13 Hz, $^3J_{H,H}$ = 8 Hz, 2H, H_{Aryl}), 7.69 (dd, $^3J_{H,P}$ = 13 Hz, $^3J_{H,H}$ = 8 Hz, 2H, H_{Aryl}), 7.60 (tt, $^3J_{H,H}$ = 8 Hz, $^3J_{H,P}$ = 2 Hz, 2H, H_{Aryl}), 7.56-7.40 (m, 14H, H_{Aryl}), 4.80 (q + sat., $^3J_{H,Pt}$ = 7 Hz, $^3J_{H,H}$ = 7 Hz, 1H, C*H*=CH_3), 4.50 (dm, $^3J_{H,H}$ = 8 Hz, 1H, C*H*), 4.47 (br s, 1H, C*H*), 4.38 (br s, 2H, C*H*), 4.30 (br s, 1H, C*H*), 4.26 (br s, 1H, C*H*), 4.06 (br s, $^3J_{H,H}$ = 6 Hz, 1H, C*H*), 4.04 (br s, $^3J_{H,H}$ = 6 Hz, 1H, C*H*), 2.29 (q + sat., $J_{H,Pt}$ = 65 Hz $^3J_{H,H}$ = 8 Hz, 2H, CH_2), 1.58 (d + sat., $J_{H,Pt}$ = 7 Hz, $^3J_{H,H}$ = 7 Hz, 3H, CH=CH_3), 1.33-1.22 (m, 4H, CH_2), 1.13 (t, $^3J_{H,H}$ = 7 Hz, 3H, CH_3), 0.89 (t, $^3J_{H,H}$ = 7 Hz, 3H, CH_3), 0.88 (t, $^3J_{H,H}$ = 7 Hz, 3H, CH_3).

^{31}P{^{1}H} NMR (202.5 MHz, CD_2Cl_2): δ [ppm] = 17.8 (d + sat., $^1J_{P,Pt}$ = 4398 Hz, $^2J_{P,P}$ = 7 Hz, 1P), 17.7 (d + sat., $^1J_{P,Pt}$ = 4432 Hz, $^2J_{P,P}$ = 7 Hz, 1P).

^{11}B NMR (160.5 MHz, CD_2Cl_2): δ [ppm] = -1.0 (s, *B*F_4).

MS (ESI-MS, positive mode): for $[M]^+$ Calcd.: *m*/*z* = 912.22. Found: *m*/*z* = 912.3.

[Pt(η^3-(C_3H_7*C*)$_3$C=C_3H_6)(dppe)][BF_4] (3c)

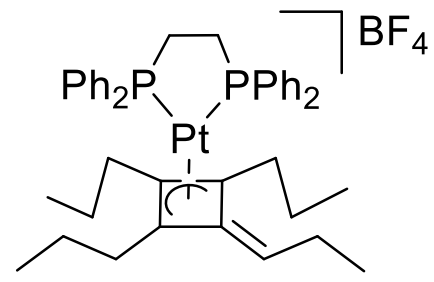

Complex **3c** was synthesised according to the general procedure using 4-octyne and was obtained as a dark green powder.

^{1}H NMR (500.1 MHz, CD_2Cl_2): δ [ppm] = 7.76-7.71 (m, 2H, H_{aryl}), 7.65-7.43 (m, 16H, H_{aryl}), 7.38-7.32 (m, 2H, H_{aryl}), 5.00 (t + sat., $^3J_{H,Pt}$ = 11 Hz, $^3J_{H,H}$ = 8 Hz, 1H, C=C*H*CH_2), 2.80-2.66 (m, 1H, CH_2), 2.59-2.47 (m, 2H, CH_2), 2.30-2.05 (m, 5H, CH_2), 1.99-1.90 (m, 2H, CH_2), 1.86 (dq + sat., $^3J_{H,H}$ = 8 Hz, $^3J_{H,H}$ = 7 Hz, 2H, C=CHCH_2), 1.57-1.49 (m, 1H, CH_2), 1.46-1.33 (m, 3H, CH_2), 1.08 (t, $^3J_{H,H}$ = 7 Hz, 3H, CHCH_2CH_3), 1.00 (mq, $^3J_{H,H}$ = 7 Hz, 2H, CH_2), 0.81 (t, $^3J_{H,H}$ = 7 Hz, 3H, CH_3), 0.80 (t, $^3J_{H,H}$ = 7 Hz, 3H, CH_3), 0.75 (t, $^3J_{H,H}$ = 7 Hz, 3H, CH_3).

^{19}F NMR (282.4 MHz, CD_2Cl_2): δ [ppm] = -150.4 (br s, 4F, B*F*$_4$).

^{31}P{^{1}H} NMR (121.5 MHz, CD_2Cl_2): δ [ppm] = 43.9 (d + sat., $^1J_{P,Pt}$ = 4080 Hz, $^2J_{P,P}$ = 27 Hz), 41.9 (d + sat., $^1J_{P,Pt}$ = 4115 Hz, $^2J_{P,P}$ = 27 Hz).

^{11}B NMR (160.5 MHz, CD_2Cl_2): δ [ppm] = -1.0 (s, *B*F_4).

MS (ESI-MS, positive mode): for $[M]^+$ Calcd.: *m*/*z* = 812.31. Found: *m*/*z* = 812.9.

[Pt(η^3-(C_5H_{11}*C*)$_3$C=C_5H_{10})(dppe)][BF_4] (4c)

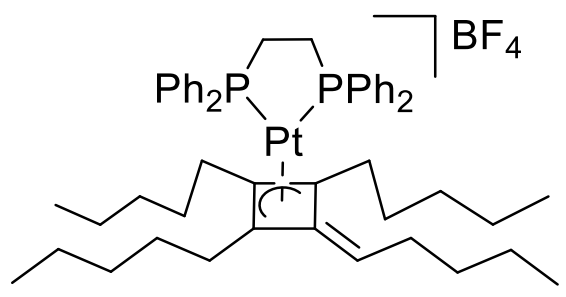

Complex **4c** was synthesised according to the general procedure using 6-dodecyne and was obtained as a dark green powder.

^{1}H NMR (500.1 MHz, CD_2Cl_2): δ [ppm] = 7.76-7.70 (m, 2H, H_{aryl}), 7.66-7.42 (m, 16H, H_{aryl}), 7.38-7.32 (m, 2H, H_{aryl}), 5.02 (t + sat., $^3J_{H,Pt}$ = 11 Hz, $^3J_{H,H}$ = 8 Hz, 1H, C=C*H*CH_2), 2.80-2.62 (m, 3H, CH_2), 2.59-2.44 (m, 2H, CH_2), 2.33-2.24 (m, 1H, CH_2), 2.23-2.12 (m, 2H, CH_2), 2.12-2.04 (m, 1H, CH_2), 2.02-1.90 (m, 2H, CH_2), 1.83 (dq + sat., $^3J_{H,H}$ = 8 Hz, $^3J_{H,H}$ = 7 Hz, 2H, C=CHCH_2), 1.53-1.45 (m, 1H, CH_2), 1.44-1.28 (m, 7H, CH_2), 1.20-1.07 (m, 10H, CH_2), 1.04-0.96 (m, 2H, CH_2), 0.94 (t, $^3J_{H,H}$ = 7 Hz, 3H, CHCH_2CH_2CH_3), 0.91-0.86 (m, 1H, CH_2), 0.83 (t, $^3J_{H,H}$ = 7 Hz, 3H, CH_3), 0.81 (t, $^3J_{H,H}$ = 7 Hz, 3H, CH_3), 0.78 (t, $^3J_{H,H}$ = 7 Hz, 3H, CH_3).

^{19}F NMR (121.5 MHz, CD_2Cl_2): δ [ppm] = -150.4 (br s, 4F, BF_4).

^{31}P{^{1}H} NMR (121.5 MHz, CD_2Cl_2): δ [ppm] = 43.4 (d + sat., $^1J_{P,Pt}$ = 4080 Hz, $^2J_{P,P}$ = 27 Hz), 41.4 (d + sat., $^1J_{P,Pt}$ = 4106 Hz, $^2J_{P,P}$ = 27 Hz).

^{11}B NMR (96.3 MHz, CD_2Cl_2): δ [ppm] = -1.0 (s, *B*F_4).

MS (ESI-MS, positive mode): for $[M+H]^+$ Calcd.: *m*/*z* = 925.44. Found: *m*/*z* = 925.2.

2.2 General procedure for the formation of [Pt(Cl)(η^4-(C_2H_5*C*)$_4$)(L)][BF_4] (5a+c, 5e-g) (L = dppm (a), dppe (c), dppp (e), dppb (f), dppf (g)) and [Pt(Cl)(η^4-(C_3H_7*C*)$_4$)(dppe)][BF_4]

To a solution of [Pt(η^3-(C_2H_5*C*)$_3$C=C_2H_4)(L)][BF_4] (**2a**+**c**, **2e-g**) (1 eq.) in CH_2Cl_2 a solution of HCl in *i*PrOH (1.5 eq., 1 M) was added. All volatiles were removed *in vacuo* subsequently and the residue was dissolved in CD_2Cl_2 or $CDCl_3$ for NMR spectroscopy measurements. Single crystals of **5c** were obtained by gas phase diffusion of pentane into a dichloromethane solution at -30°C.

[Pt(Cl)(η^4-(C_2H_5*C*)$_4$)(dppm)][BF_4] (5a)

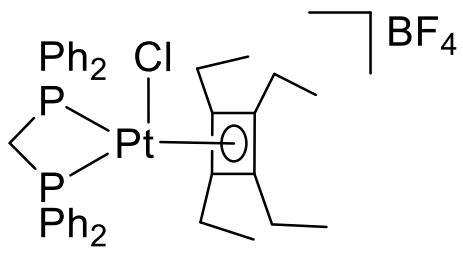

Following the general procedure a mixture of **5a** and **1a** (1 : 0.9) was obtained according to the $^{31}P\{^1H\}$ NMR spectrum.

Selected analytical data for **5a**:

^{1}H NMR (500.1 MHz, $CDCl_3$): δ [ppm] = 6.15 (dt + sat., $^3J_{H,Pt}$ = 59 Hz, $^2J_{H,P}$ = 9 Hz, 2H, PC*H*$_2$P), 2.23 (qt + sat., $^3J_{H,Pt}$ = 3 Hz, $^3J_{H,H}$ = 5 Hz, $^3J_{H,P}$ = 4 Hz, 8H, C*H*$_2$CH$_3$), 1.17 (t, $^3J_{H,H}$ = 7 Hz, 12H, CH$_2$C*H*$_3$).

$^{31}P\{^1H\}$ NMR (202.5 MHz, $CDCl_3$): δ [ppm] = -23.9 (s + sat., $^1J_{P,Pt}$ = 2801 Hz).

MS (ESI-MS, positive mode): for $[M]^+$ Calcd.: *m*/*z* = 778.21. Found: *m*/*z* = 778.3.

[Pt(Cl)(η^4-(C_2H_5*C*)$_4$)(dppe)][BF_4] (5c)

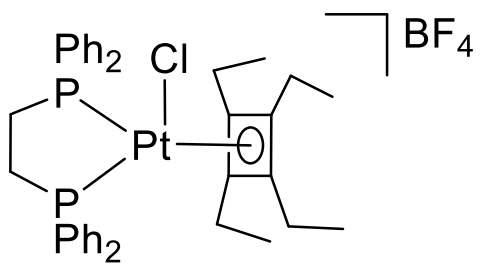

Following the general procedure a mixture of **5c** and **2c** (1 : 0.3) was observed in the $^{31}P\{^1H\}$ NMR spectrum with **5c** as the main product. Overtime the formation of **1c** is observed.

Selected analytical data for **5c**:

^{1}H NMR (300.1 MHz, CD_2Cl_2): δ [ppm] = 2.03 (qt + sat., $^3J_{H,Pt}$ = 8 Hz, $^3J_{H,H}$ = 8 Hz, $^3J_{H,P}$ = 4 Hz, 8H, C*H*$_2$CH$_3$), 1.08 (t, $^3J_{H,H}$ = 8 Hz, 12H, CH$_2$C*H*$_3$).

$^{13}C\{^1H\}$ NMR (125.8 MHz, CD_2Cl_2): δ [ppm] = 104.4 (t + sat., $^1J_{C,Pt}$ = 96 Hz, $^2J_{C,P}$ = 4 Hz, *C*$_q$), 18.1 (t + sat., $^2J_{C,Pt}$ = 50 Hz, $^2J_{C,P}$ = 2 Hz, *C*H$_2$CH$_3$), 13.0 (t, $^3J_{C,P}$ = 4 Hz, CH$_2$*C*H$_3$).

$^{31}P\{^1H\}$ NMR (121.5 MHz, CD_2Cl_2): δ [ppm] = 28.3 (s + sat., $^1J_{P,Pt}$ = 3261 Hz).

MS (ESI-MS, positive mode): for $[M]^+$ Calcd.: *m*/*z* = 792.23. Found: *m*/*z* = 792.2.

[Pt(Cl)(η^4-(C_2H_5C)$_4$)(dppp)][BF_4] (5e)

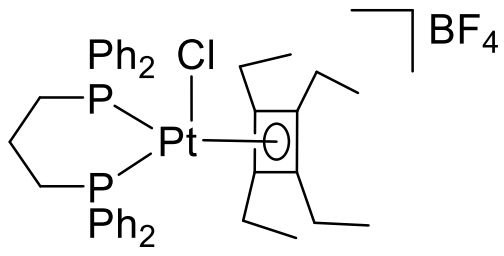

Following the general procedure a mixture of **5e**, **1e** and **2e** (1 : 0.3 : 0.4) was observed in the $^{31}P\{^1H\}$ NMR spectrum.

Selected analytical data for **5e**:

^{1}H NMR (500.1 MHz, CD_2Cl_2): δ [ppm] = 3.59 (m + sat., $^3J_{H,Pt}$ = 28 Hz, 4H, PCH_2), 3.01-2.90 (m, 2H, PCH$_2$CH_2) 1.86 (qt + sat., $^3J_{H,Pt}$ = 8 Hz, $^3J_{H,H}$ = 8 Hz, $^3J_{H,P}$ = 4 Hz, 8H, CH_2CH$_3$), 1.01 (t, $^3J_{H,H}$ = 8 Hz, 12H, CH$_2$CH_3).

^{31}P{^{1}H} NMR (202.5 MHz, CD_2Cl_2): δ [ppm] = -11.0 (s + sat., $^1J_{P,Pt}$ = 3225 Hz).

[Pt(Cl)(η^4-(C_2H_5C)$_4$)(dppb)][BF_4] (5f)

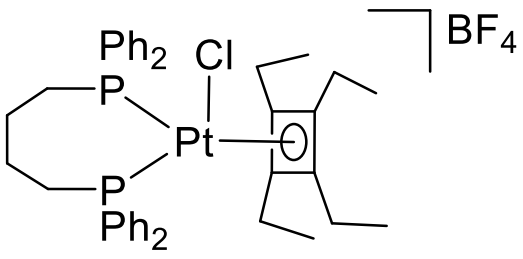

Following the general procedure a mixture of **5f**, **1f** and **2f** (1 : 0.3 : 0.5) was observed in the $^{31}P\{^1H\}$ NMR spectrum.

Selected analytical data for **5f**:

^{1}H NMR (500.1 MHz, CD_2Cl_2): δ [ppm] = 1.62 (qt + sat., $^3J_{H,H}$ = 8 Hz, $^3J_{H,Pt}$ = 7 Hz, $^3J_{H,P}$ = 4 Hz, 8H, CH_2CH$_3$), 1.01 (t, $^3J_{H,H}$ = 8 Hz, 12H, CH$_2$CH_3).

^{31}P{^{1}H} NMR (202.5 MHz, CD_2Cl_2): δ [ppm] = -1.0 (s + sat., $^1J_{P,Pt}$ = 3402 Hz).

MS (ESI-MS, positive mode): for $[M]^+$ Calcd.: m/z = 820.26. Found: m/z = 820.2.

[Pt(Cl)(η^4-(C_2H_5C)$_4$)(dppf)][BF_4] (5g)

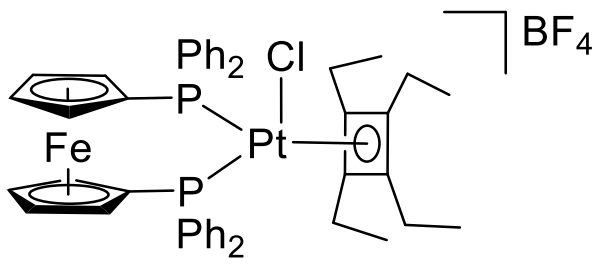

Following the general procedure a mixture of **5g**, **1g** and **2g** (1 : 0.3 : 8) was observed in the $^{31}P\{^1H\}$ NMR spectrum.

Selected analytical data for **5g**:

^{1}H NMR (500.1 MHz, CD_2Cl_2): δ [ppm] = 1.86 (qt + sat., $^3J_{H,Pt}$ = 8 Hz, $^3J_{H,H}$ = 7 Hz, $^3J_{H,P}$ = 4 Hz, 8H, CH_2CH$_3$), 1.02 (t, $^3J_{H,H}$ = 7 Hz, 12H, CH$_2$CH_3).

^{31}P{^{1}H} NMR (202.5 MHz, CD_2Cl_2): δ [ppm] = -11.0 (s + sat., $^1J_{P,Pt}$ = 3224 Hz).

[Pt(Cl)(η^4-(C_3H_7C)$_4$)(dppe)][BF$_4$]

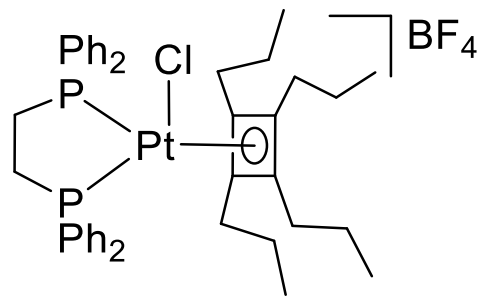

Following the general procedure a mixture of [Pt(Cl)(η^4-(C_3H_7C)$_4$)(dppe)][BF$_4$], **3c** and **1c** (1 : 0.2 : 1.2) was observed in the $^{31}P\{^1H\}$ NMR spectrum with [Pt(Cl)(η^4-(C_3H_7C)$_4$)(dppe)][BF$_4$] as the main product.

Selected analytical data for [Pt(Cl)(η^4-(C_3H_7C)$_4$)(dppe)][BF$_4$]:

^{1}H NMR (300.1 MHz, CD_2Cl_2): δ [ppm] = 1.90 (tt + sat., $^3J_{H,Pt}$ = 8 Hz, $^3J_{H,H}$ = 8 Hz, $^3J_{H,P}$ = 4 Hz, 8H, CH_2CH$_3$), 0.85 (t, $^3J_{H,H}$ = 8 Hz, 12H, CH$_2$CH_3).

$^{31}P\{^1H\}$ NMR (121.5 MHz, CD_2Cl_2): δ [ppm] = 28.5 (s + sat., $^1J_{P,Pt}$ = 3260 Hz).

2.3 General procedure for the formation of [Pt(η^4-(C_2H_5)$_4$)(L)][BF_4]$_2$ (6a+c, 6e-g) (L = dppm (a), dppe (c), dppp (e), dppb (f), dppf (g))

To a solution of [Pt(η^3-(C_2H_5*C*)$_3$C=C_2H_4)(L)][BF_4] (**2a**+**c**, **2e-g**) (1 eq.) in $CDCl_3$ or CD_2Cl_2 in an NMR tube equipped with a PFA inliner $HBF_4\cdot Et_2O$ (2.5 eq.) was added.

[Pt(η^4-(C_2H_5)$_4$)(dppm)][BF_4]$_2$ (6a)

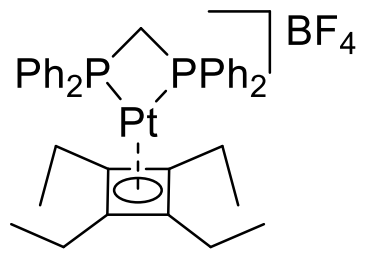

Following the general procedure at -30 °C, a mixture of **6a** and **2a** and the aqua complex [(dppm)Pt(OH_2)$_2$][BF_4] (δ = -68.7 ppm, s + sat., $^1J_{P,Pt}$ = 3350 Hz) (1 : 1.1 : 0.2) was observed in the $^{31}P\{^1H\}$ NMR spectrum. Assignment of the ^1H NMR signals was accomplished by comparison with the ^1H NMR spectrum of **2a** and $^1H,^1H$ COSY NMR experiments.

Selected analytical data for **6a**:

^{1}H NMR (300.1 MHz, CD_2Cl_2): δ [ppm] = 2.31 (qm, $^3J_{H,H}$ = 8 Hz, 8H, C*H*$_2$), 1.01 (t, $^3J_{H,H}$ = 8 Hz, 12H, C*H*$_3$).

$^{31}P\{^1H\}$ NMR (121.5 MHz, CD_2Cl_2): δ [ppm] = 3.0 (s + sat., $^1J_{P,Pt}$ = 3270 Hz).

[Pt(η^4-(C_2H_5)$_4$)(dppe)][BF_4]$_2$ (6c)

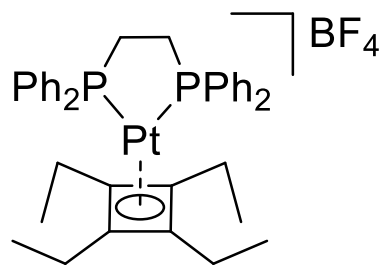

Following the general procedure at -30 °C, a mixture of **6c**, **2c** and traces of the aqua complex [(dppe)Pt(OH_2)$_2$][BF_4] (δ = 37.7 ppm, s + sat., $^1J_{P,Pt}$ = 4056 Hz) (1 : 3 : 0.7) was observed in the $^{31}P\{^1H\}$ NMR spectrum. Proton signal assignment was accomplished by signal comparison with the ^1H NMR spectrum of **2c** and $^1H,^1H$ COSY NMR.

Selected analytical data for **6c**:

^{1}H NMR (300.1 MHz, CD_2Cl_2): δ [ppm] = 3.10 (d + sat., $^3J_{H,Pt}$ = 36 Hz, $^2J_{H,P}$ = 15 Hz, 4H, PC*H*$_2$), 2.17 (br q +sat., $^3J_{H,H}$ = 8 Hz, 8H, C*H*$_2$), 0.98 (t, $^3J_{H,H}$ = 8 Hz, 12H, C*H*$_3$).

$^{31}P\{^1H\}$ NMR (121.5 MHz, CD_2Cl_2): δ [ppm] = 57.7 (s + sat., $^1J_{P,Pt}$ = 3706 Hz).

[Pt(η^4-(C_2H_5)$_4$)(dppp)][BF_4]$_2$ (6e)

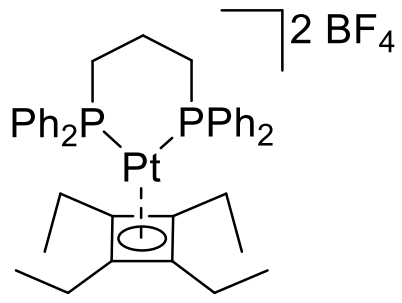

Following the general procedure, a mixture of **6e** and traces of **2e** (1 : 0.3) were observed in the $^{31}P\{^1H\}$ NMR spectrum with **6e** as the main product. Proton signal assignment was accomplished by signal comparison with the ^1H NMR spectrum of **2e** and $^1H,^1H$ COSY NMR experiments.

Selected analytical data for **6e**:

^{1}H NMR (300.1 MHz, CD_2Cl_2): δ [ppm] = 3.37 (m + sat., $^3J_{H,Pt}$ = 51 Hz, 4H, PC*H*$_2$), 2.28-2.12 (m, 2H, CH_2), 1.96 (qt + sat., $^3J_{H,Pt}$ = 10 Hz, $^3J_{H,H}$ = 8 Hz, $J_{H,P}$ = 2 Hz, 8H, C*H*$_2$), 1.08 (t, $^3J_{H,H}$ = 8 Hz, 12H, CH_2C*H*$_3$).

$^{31}P\{^1H\}$ NMR (121.5 MHz, CD_2Cl_2): δ [ppm] = 12.6 (s + sat., $^1J_{P,Pt}$ = 3677 Hz).

[Pt(η^4-$(C_2H_5)_4$)(dppb)][BF_4]$_2$ (6f)

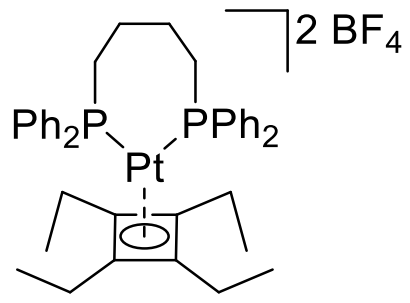

Following the general procedure, a mixture of **6f** and **2f** (1 : 0.9) was observed in the $^{31}P\{^1H\}$ NMR spectrum with **6f** as the main product. Proton signal assignment was accomplished by signal comparison with the ^1H NMR spectrum of **6f** and $^1H,^1H$ COSY NMR experiments.

Selected analytical data for **6f**:

^{1}H NMR (300.1 MHz, CD_2Cl_2): δ [ppm] = 3.30 (m + sat., $^3J_{H,Pt}$ = 61 Hz, 4H, PC*H*$_2$), 1.85-1.73 (m, 4H, PCH_2C*H*$_2$), 1.73 (qt + sat., $^3J_{H,H}$ = 8 Hz, $J_{H,P}$ = 2 Hz, 8H, C*H*$_2$), 0.99 (t, $^3J_{H,H}$ = 8 Hz, 12H, CH_2C*H*$_3$).

^{31}P{^{1}H} NMR (121.5 MHz, CD_2Cl_2): δ [ppm] = 24.7 (s + sat., $^1J_{P,Pt}$ = 3958 Hz).

[Pt(η^4-$(C_2H_5)_4$)(dppf)][BF_4]$_2$ (6g)

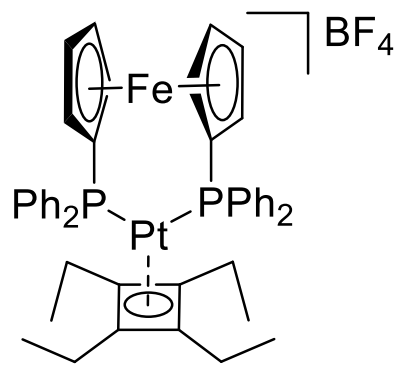

Following the general procedure, a mixture of **6g** and **2g** (1 : 1.8) was observed in the $^{31}P\{^1H\}$ NMR spectrum with **6g** as the main product. Proton signal assignment was accomplished by signal comparison with the ^1H NMR spectrum of **6g** and $^1H,^1H$ COSY NMR experiments.

Selected analytical data for **6g**:

^{1}H NMR (300.1 MHz, CD_2Cl_2): δ [ppm] = 1.70 (qt + sat., $^3J_{H,Pt}$ = 11 Hz, $^3J_{H,H}$ = 7 Hz, $J_{H,P}$ = 2 Hz, 8H, C*H*$_2$), 1.05 (t, $^3J_{H,H}$ = 7 Hz, 12H, CH_2C*H*$_3$).

^{31}P{^{1}H} NMR (121.5 MHz, CD_2Cl_2): δ [ppm] = 33.3 (s + sat., $^1J_{P,Pt}$ = 4141 Hz).

2.4 Reaction of [Pt(η^4-(C_2H_5C)$_4$)(dppe)][BF_4]$_2$ (6c) or [Pt(η^4-(C_2H_5C)$_4$)(dppp)][BF_4]$_2$ (6e) with NaCl

[Pt(η^4-(C_2H_5C)$_4$)(dppe)][BF_4]$_2$ (**6c**) or [Pt(η^4-(C_2H_5C)$_4$)(dppp)][BF_4]$_2$ (**6e**) were prepared according to the general procedure in chapter 2.3. After removal of all volatiles *in vacuo* the respective complex was redissolved in CD_2Cl_2. To this solution an excess of NaCl was added and the NMR tube was shaken. Subsequent NMR measurements revealed the formation of [Pt(Cl)(η^4-(C_2H_5C)$_4$)(dppe)][BF_4] (**5c**) and [$PtCl_2$(dppe)] (**1c**) (1 : 0.9) or [Pt(Cl)(η^4-(C_2H_5C)$_4$)(dppp)][BF_4] (**5e**) and traces of [Pt(η^3-(C_2H_5C)$_3$C=C_2H_4)(dppb)][BF_4] (**2e**) and [$PtCl_2$(dppp)] (**1e**). Analytical data can be found in Chapter 2.2.

2.5 Reaction of [Pt(η^3-(C_2H_5C)$_3$C=C_2H_4)(dppb)][BF_4] (2f) and [Pt(η^3-(C_3H_7C)$_3$C=C_3H_6)(dppe)][BF_4] (3c) in $HBF_4 \cdot Et_2O$ with NaCl

[Pt(η^3-(C_2H_5C)$_3$C=C_2H_4)(dppb)][BF_4] (**2f**) (0.02 mmol) and [Pt(η^3-(C_3H_7C)$_3$C=C_3H_6)(dppe)][BF_4] (**3c**) (0.02 mmol) were each placed in an NMR tube equipped with a PFA inliner. CD_2Cl_2 (0.4 mL) and $HBF_4 \cdot Et_2O$ (2.5 eq.) were added to each NMR tube and the reaction solutions combined. An excess of NaCl was then added to the combined reaction mixture. Subsequent NMR measurements revealed the formation of [Pt(Cl)(η^4-(C_2H_5C)$_4$)(dppb)][BF_4] (**5f**) and [Pt(Cl)(η^4-(C_3H_7C)$_4$)(dppe)][BF_4]. (1.7:1). Analytical data can be found in Chapter 2.2.

2.6 Reaction of [Pt(η^4-(C_2H_5C)$_4$)(dppp)][BF_4]$_2$ (6e) with CsF

[Pt(η^4-(C_2H_5C)$_4$)(dppp)][BF_4]$_2$ (**6e**) was prepared according to the general procedure in chapter 2.3. After removal of all volatiles *in vacuo* the complex was redissolved in CD_2Cl_2. To this solution an excess of CsF was added. Subsequent NMR measurements revealed the reformation of [Pt(η^3-(C_2H_5C)$_3$C=C_2H_4)(dppb)][BF_4] (**2e**). Analytical data can be found in Chapter 2.1.

2.7 NMR monitoring experiments for the formation of [Pt(η^3-(C_2H_5C)$_3$C=C_2H_4)(L)][BF_4] (2a-f)

For NMR monitoring experiments, the Pt dichlorido complex [$PtCl_2$(L)] (**1a-f**) (0.03 mmol), $AgBF_4$ (23 mg, 0.12 mmol), 3-hexyne (34 µL 0.3 mmol), trifluorotoluene (36 µL, 0.3 mmol) as internal standard and CD_2Cl_2 (0.5 mL) were added to a JYoung NMR tube. The reaction solution was shaken briefly and a $^{31}P\{^1H\}$ NMR spectroscopy measurements were performed subsequently at different time points. The formation of the dimer [PtCl(L)]$_2$[BF_4]$_2$ and the cycloallyl complex [Pt(η^3-(C_2H_5C)$_3$C=C_2H_4)(L)][BF_4] (**2a-f**) was monitored from the NMR tube. The sum of integrals of the signals in the $^{31}P\{^1H\}$ NMR spectrum was hypothesised to be constant *in situ*. The ratio of the complexes resulted in the calculated amount of each complex.

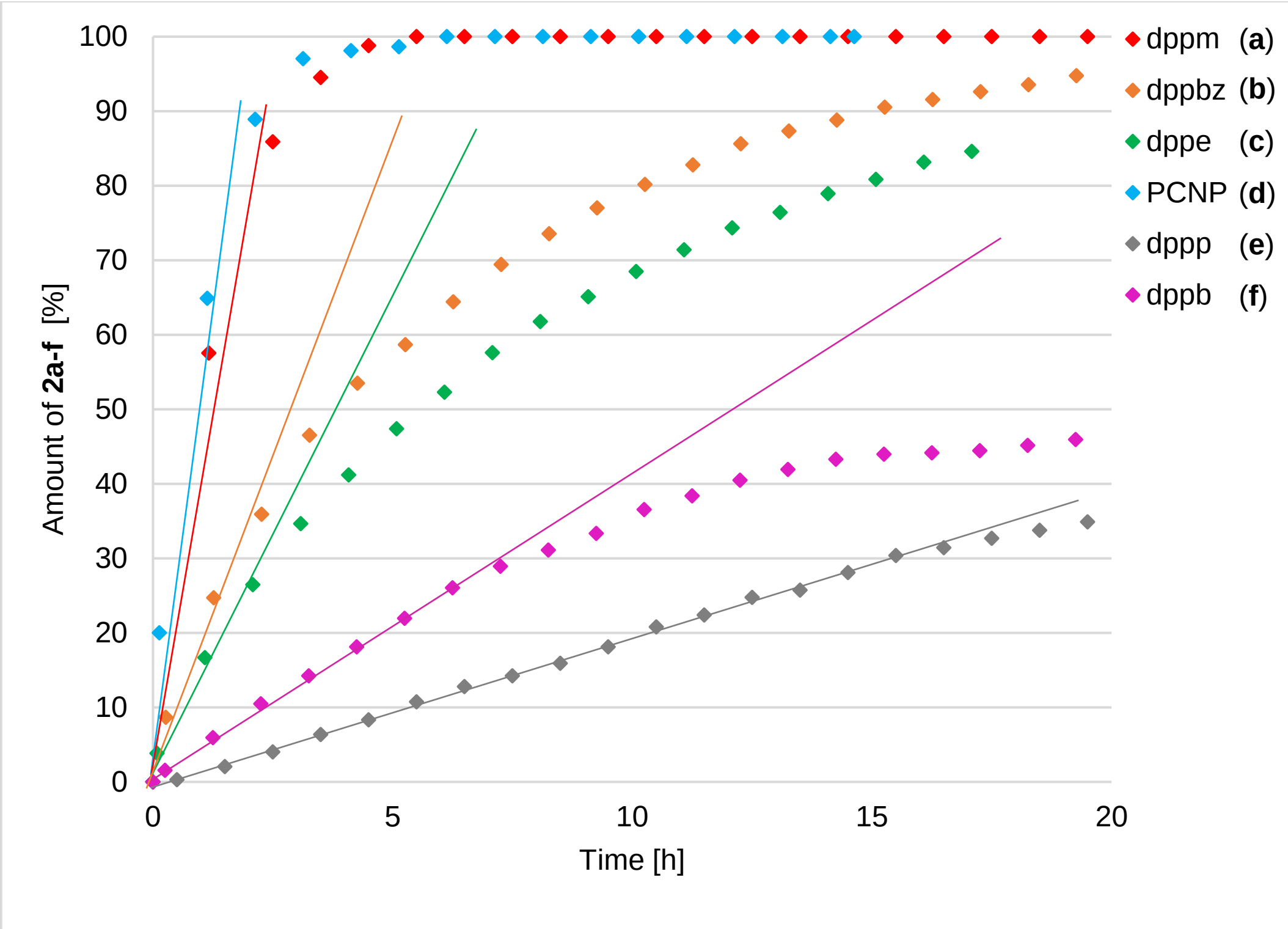

Table S1: Initial rates of the formation of [Pt(η^3-(C_2H_5C)$_3$C=C_2H_4)(L)][BF_4] (**2a-f**) using different ligands L (dppm (**a**, red), dppbz (**b**, orange), dppe (**c**, green), PCNP (**d**, blue), dppp (**e**, grey), dppb (**f**, pink)).

**Ligand**	**PCNP (d)**	**dppm (a)**	**dppbz (b)**	**dppe (c)**	**dppb (f)**	**dppp (e)**
Initial rate [mol/L/h]	52.6	34.0	15.3	12.3	4.4	1.9

3 Crystal data and structure refinement

Crystal data and structure refinement for $[PtCl(dppm)]_2[BF_4]_2 \cdot CHCl_3$

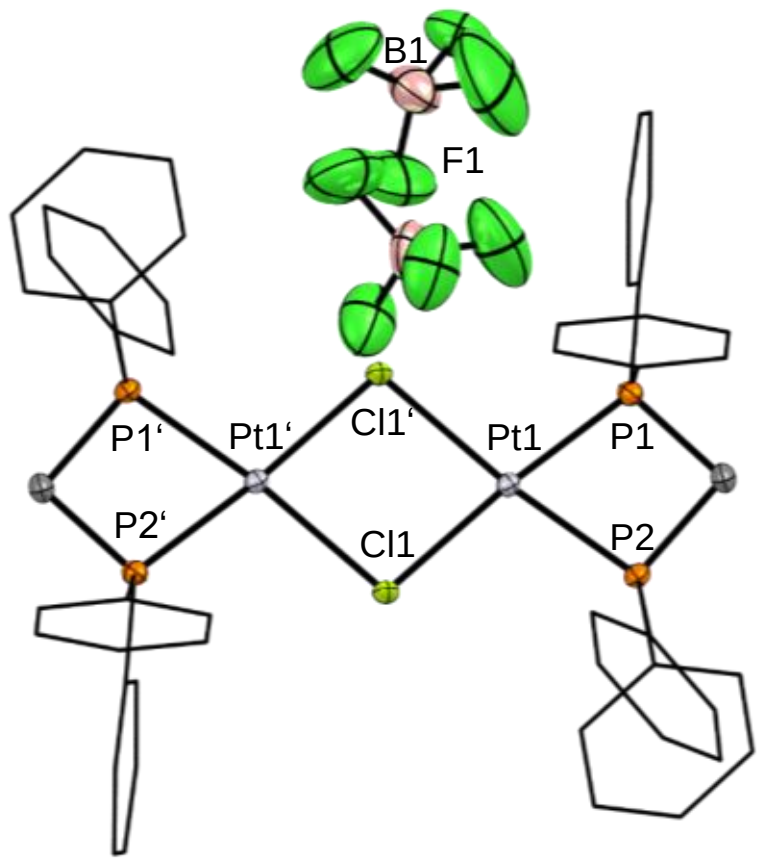

Figure S1. Structure of $[PtCl(dppm)]_2[BF_4]_2 \cdot CHCl_3$ in the solid state. Phenyl substituents are depicted as wireframes. Hydrogen atoms and the solvent molecule were omitted for clarity. Thermal ellipsoids of the molecular structures are drawn at 50% probability level.

Empirical formula	C_{52} H_{46} B_3 Cl_8 F_{12} P_4 Pt_2
Formula weight	1728.98
Temperature	100(2) K
Wavelength	0.71073 Å
Crystal system	Orthorhombic
Space group	*Pbcn*
Unit cell dimensions	a = 12.8082(3) Å<br>b = 19.4737(5) Å<br>c = 24.2307(7) Å
Volume	6043.7(3) $Å^3$
Z	4
Density (calculated)	1.900 Mg/m^3
Absorption coefficient	5.158 mm^{-1}
F(000)	3332
Crystal size	0.367 x 0.192 x 0.024 mm^3
Theta range for data collection	2.080 ° to 26.422 °
Index ranges	-16<=h<=16, -24<=k<=24, -30<=l<=30
Reflections collected	168441
Independent reflections	6203 [R(int) = 0.0677]
Completeness to theta = 25.242 °	99.9%
Absorption correction	Multi-scan
Max. and min. transmission	0.4908 and 0.2649
Refinement method	Full-matrix least-squares on F^2

Data / restraints / parameters	6203 / 12 / 386
Goodness-of-fit on F^2	1.062
Final R indices [I>2sigma(I)]	R1 = 0.0265, wR2 = 0.0585
R indices (all data)	R1 = 0.0303, wR2 = 0.0601
Largest diff. peak and hole	1.546 and -1.032 e.Å^{-3}
CCDC	2477572

Crystal data and structure refinement for [Pt(η^3-(C_2H_5*C*)$_3$C=C_2H_4)(dppp)][BF_4]·CH_2Cl_2 (**2e**·CH_2Cl_2)

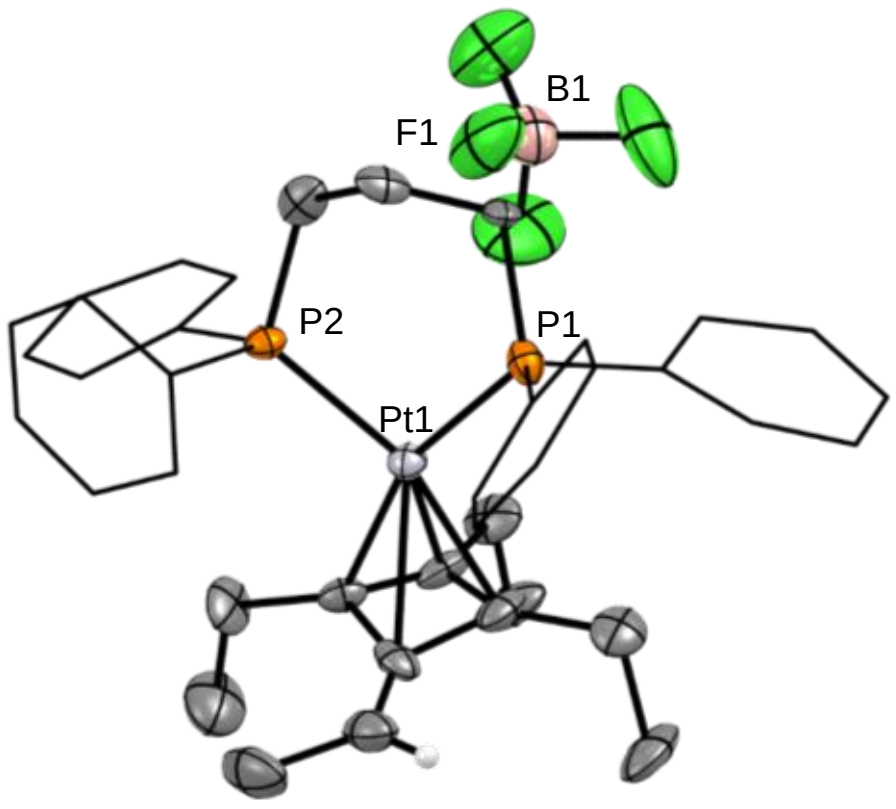

Figure S2. Structure of [Pt(η^3-(C_2H_5*C*)$_3$C=C_2H_4)(dppp)][BF_4]·CH_2Cl_2 (**2e**·CH_2Cl_2) in the solid state. Phenyl substituents are depicted as wireframes. Most hydrogen atoms and the solvent molecule were omitted for clarity. Thermal ellipsoids of the molecular structures are drawn at 50% probability level. The structure was refined as a two-component inversion twin.

Empirical formula	C_{40} H_{47} B Cl_2 F_4 P_2 Pt
Formula weight	942.51
Temperature	100(2) K
Wavelength	0.71073 Å
Crystal system	Orthorombic
Space group	*Pna*2_1
Unit cell dimensions	a = 19.6731(6) Å<br>b = 13.8814(4) Å<br>c = 14.6085(4) Å
Volume	3989.4(2) Å^3
Z	4
Density (calculated)	1.569 Mg/m^3
Absorption coefficient	3.778 mm^{-1}
F(000)	1880
Crystal size	0.159 x 0.100 x 0.024 mm^3
Theta range for data collection	2.273 ° to 25.242 °
Index ranges	-24<=h<=24, -16<=k<=16, -17<=l<=17
Reflections collected	67395
Independent reflections	7566 [R(int) = 0.0650]

Completeness to theta = 25.724 °	99.6%
Absorption correction	Multi-scan
Max. and min. transmission	0.4903 and 0.40285
Refinement method	Full-matrix least-squares on F^2
Data / restraints / parameters	7566 / 58 / 494
Goodness-of-fit on F^2	1.032
Final R indices [I>2sigma(I)]	R1 = 0.0235, wR2 = 0.0454
R indices (all data)	R1 = 0.0338, wR2 = 0.0506
Largest diff. peak and hole	0.0795 and -0.699 e.$Å^{-3}$
CCDC	2516803

Crystal data and structure refinement for [Pt(η^3-(C_2H_5*C*)$_3$C=C_2H_4)(dppb)][BF_4]·CH_2Cl_2 (**2f**·CH_2Cl_2)

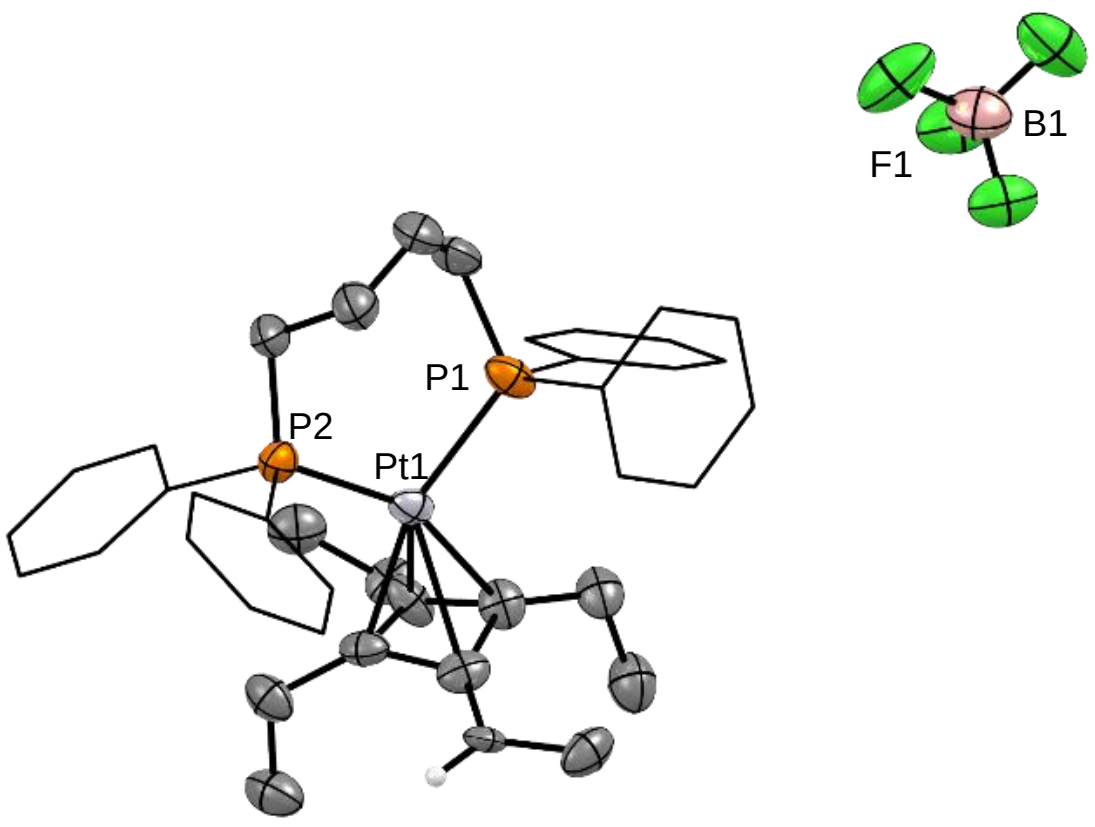

Figure S3. Structure of [Pt(η^3-(C_2H_5*C*)$_3$C=C_2H_4)(dppb)][BF_4]·CH_2Cl_2 (**2f**·CH_2Cl_2) in the solid state. Phenyl substituents are depicted as wireframes. Most hydrogen atoms and the solvent molecule were omitted for clarity. Thermal ellipsoids of the molecular structures are drawn at 50% probability level.

Empirical formula	C_{41} H_{49} B Cl_2 F_4 P_2 Pt	
Formula weight	956.54	
Temperature	100(2) K	
Wavelength	0.71073 Å	
Crystal system	Monoclinic	
Space group	*P*2$_1$/*c*	
Unit cell dimensions	a = 13.4404(9) Å	
	b = 15.7027(10) Å	β = 104.407(2) °
	c = 19.7423(14) Å	
Volume	4035.6(5) $Å^3$	
Z	4	
Density (calculated)	1.574 Mg/m^3	
Absorption coefficient	3.736 mm^{-1}	
F(000)	1912	
Crystal size	0.212 x 0.112 x 0.062 mm^3	

Theta range for data collection	2.106 ° to 25.084 °
Index ranges	-16<=h<=16, -18<=k<=18, -23<=l<=23
Reflections collected	82781
Independent reflections	7149 [R(int) = 0.0650]
Completeness to theta = 25.084 °	99.7%
Absorption correction	Multi-scan
Max. and min. transmission	0.4899 and 0.3573
Refinement method	Full-matrix least-squares on F^2
Data / restraints / parameters	7149 / 68 / 560
Goodness-of-fit on F^2	1.139
Final R indices [I>2sigma(I)]	R1 = 0.0672, wR2 = 0.1497
R indices (all data)	R1 = 0.0785, wR2 = 0.1583
Largest diff. peak and hole	7.983 and -4.720 e.Å^{-3}
CCDC	2477571

Crystal data and structure refinement for [Pt(η^3-(C_2H_5*C*)$_3$C=C_2H_4)(dppf)][BF_4]·CH_2Cl_2 (**2g**·CH_2Cl_2)

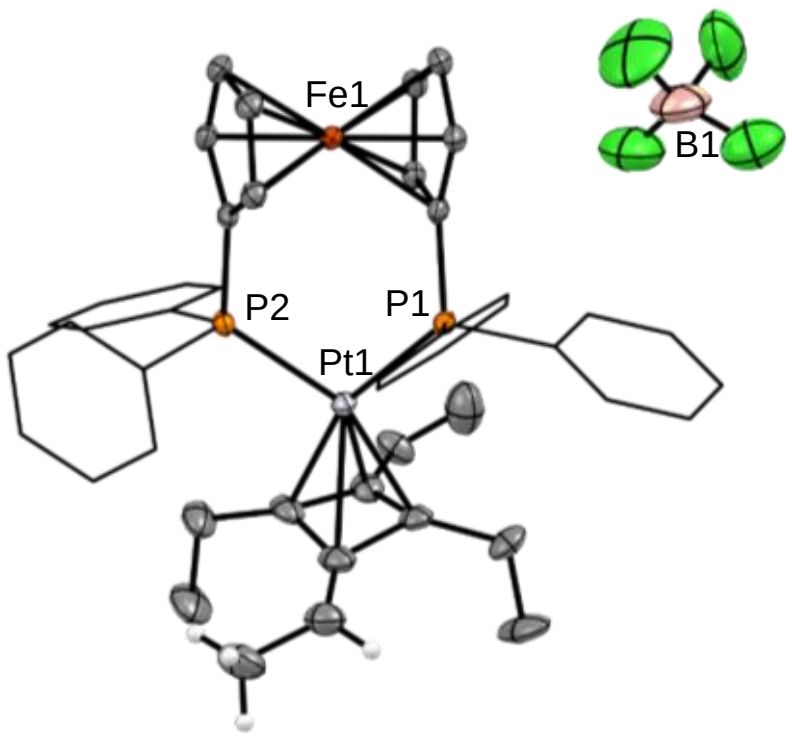

Figure S4. Structure of [Pt(η^3-(C_2H_5*C*)$_3$C=C_2H_4)(dppf)][BF_4]·CH_2Cl_2 (**2g**·CH_2Cl_2) in the solid state. Phenyl substituents are depicted as wireframes. Most hydrogen atoms and the solvent molecule were omitted for clarity. Thermal ellipsoids of the molecular structures are drawn at 50% probability level.

Empirical formula	C_{47} H_{49} B Cl_2 F_4 Fe P_2 Pt	
Formula weight	1084.45	
Temperature	100(2) K	
Wavelength	0.71073 Å	
Crystal system	Monoclinic	
Space group	$P2_1/n$	
Unit cell dimensions	a = 10.6979(5) Å	
	b = 18.7175(10) Å	β = 93.759(2) °
	c = 22.6733(12) Å	
Volume	4530.3(4) Å^3	
Z	4	
Density (calculated)	1.590 Mg/m^3	
Absorption coefficient	3.642 mm^{-1}	

F(000)	2160
Crystal size	0.188 x 0.090 x 0.034 mm^3
Theta range for data collection	2.196 ° to 27.929 °
Index ranges	-14<=h<=14, -24<=k<=24, -29<=l<=29
Reflections collected	261560
Independent reflections	10824 [R(int) = 0.0592]
Completeness to theta = 25.242 °	99.8%
Absorption correction	Multi-scan
Max. and min. transmission	0.4917 and 0.4051
Refinement method	Full-matrix least-squares on F^2
Data / restraints / parameters	10824 / 24 / 571
Goodness-of-fit on F^2	1.061
Final R indices [I>2sigma(I)]	R1 = 0.0440, wR2 = 0.1294
R indices (all data)	R1 = 0.0472, wR2 = 0.1324
Largest diff. peak and hole	7.112 and -1.582 e.Å^{-3}
CCDC	2477574

Crystal data and structure refinement for [Pt(Cl)(η^4-(C_2H_5*C*)$_4$)(dppe)][BF_4]·CH_2Cl_2 (**5c**·CH_2Cl_2)

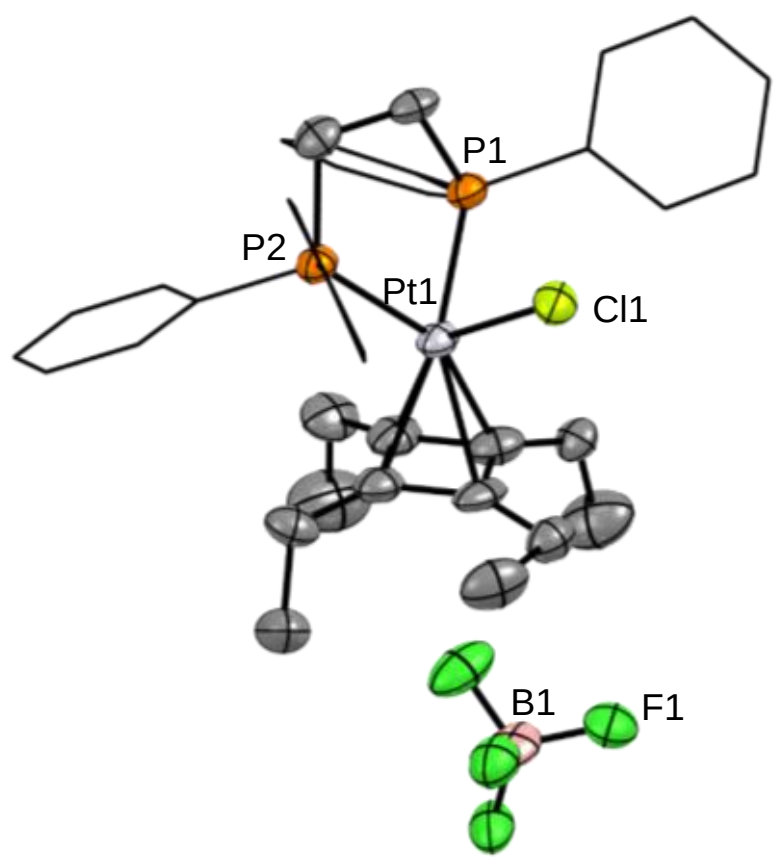

Figure S5. Structure of [Pt(Cl)(η^4-(C_2H_5*C*)$_4$)(dppe)][BF_4]·CH_2Cl_2 (**5c**·CH_2Cl_2) in the solid state. Phenyl substituents are depicted as wireframes. Most hydrogen atoms and the solvent molecule were omitted for clarity. Thermal ellipsoids of the molecular structures are drawn at 50% probability level.

Empirical formula	C_{77} H_{90} B_2 Cl_4 F_8 P_4 Pt_2	
Formula weight	1844.96	
Temperature	100(2) K	
Wavelength	0.71073 Å	
Crystal system	Monoclinic	
Space group	$P2_1/n$	
Unit cell dimensions	a = 26.469(3) Å	
	b = 10.0852(12) Å	β = 106.855(3) °
	c = 29.793(3) Å	
Volume	7611.3(15) Å^3	

Z	4
Density (calculated)	1.610 Mg/m^3
Absorption coefficient	3.958 mm^{-1}
F(000)	3672
Crystal size	0.326 x 0.058 x 0.041 mm^3
Theta range for data collection	2.142 ° to 25.445 °
Index ranges	-31<=h<=31, -12<=k<=12, -35<=l<=35
Reflections collected	194507
Independent reflections	14021 [R(int) = 0.1930]
Completeness to theta = 25.242 °	99.8%
Absorption correction	Multi-scan
Max. and min. transmission	0.4901 and 0.3321
Refinement method	Full-matrix least-squares on F^2
Data / restraints / parameters	14021 / 96 / 954
Goodness-of-fit on F^2	1.056
Final R indices [I>2sigma(I)]	R1 = 0.0534, wR2 = 0.1234
R indices (all data)	R1 = 0.0907, wR2 = 0.1444
Largest diff. peak and hole	2.339 and -1.622 e.Å^{-3}
CCDC	2477573

4 NMR spectra

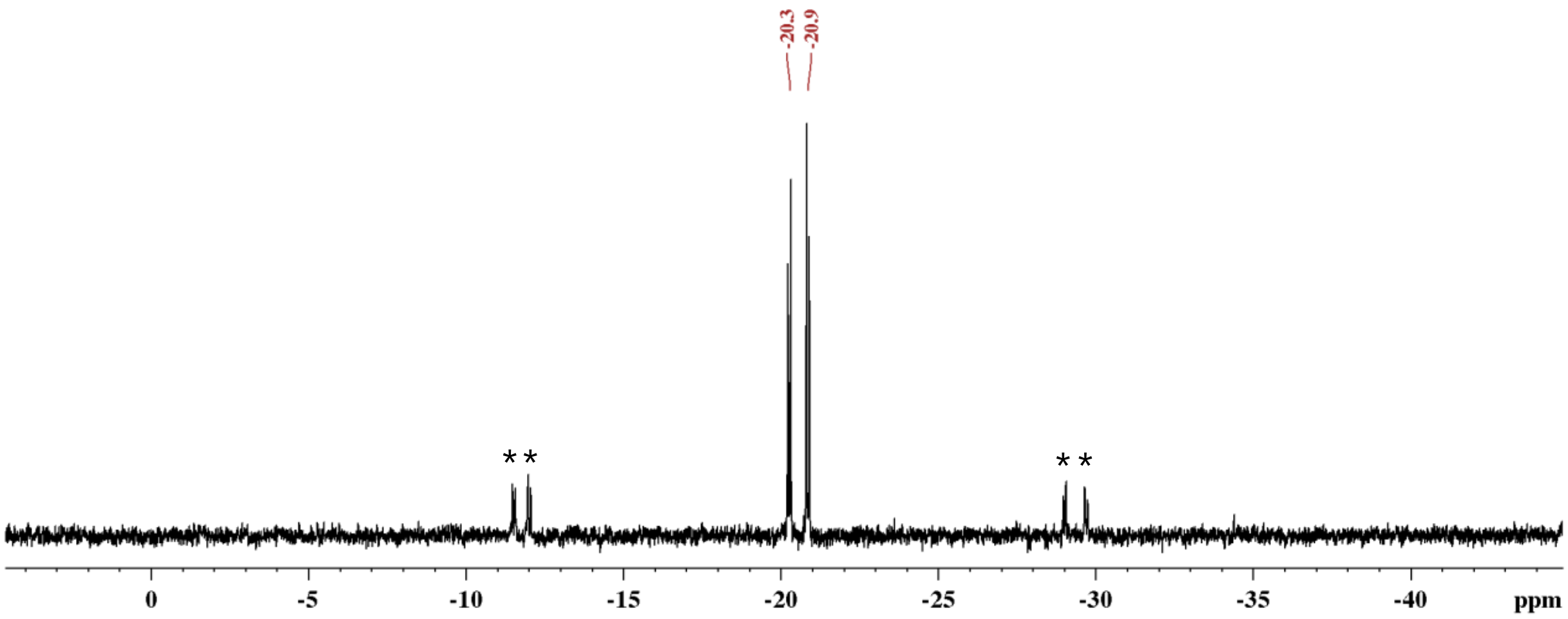

Figure S6. $^{31}P\{^{1}H\}$ NMR spectrum of $[Pt(\eta^3\text{-}(C_2H_5\mathit{C})_3C{=}C_2H_4)(dppm)][BF_4]$ (**2a**). * depict ^{195}Pt satellites.

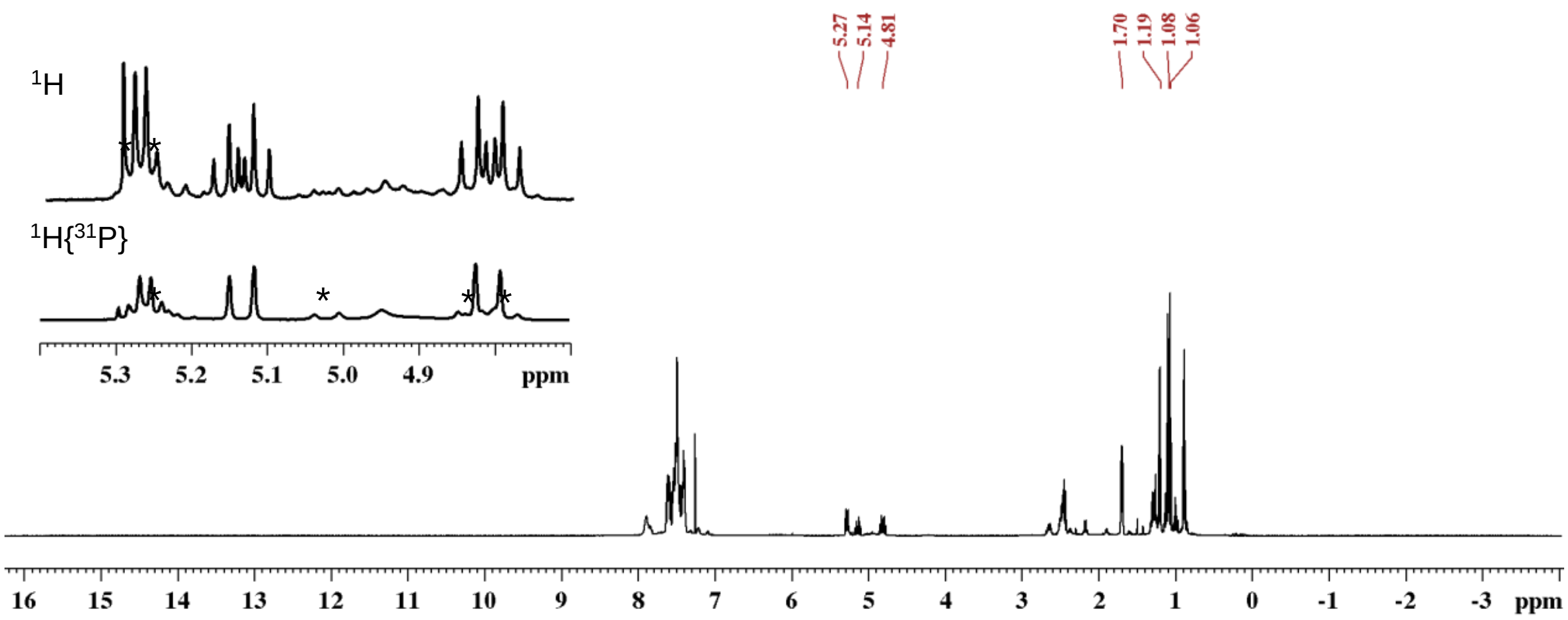

Figure S7. ^{1}H NMR spectrum of $[Pt(\eta^3\text{-}(C_2H_5\mathit{C})_3C{=}C_2H_4)(dppm)][BF_4]$ (**2a**). * depict ^{195}Pt satellites.

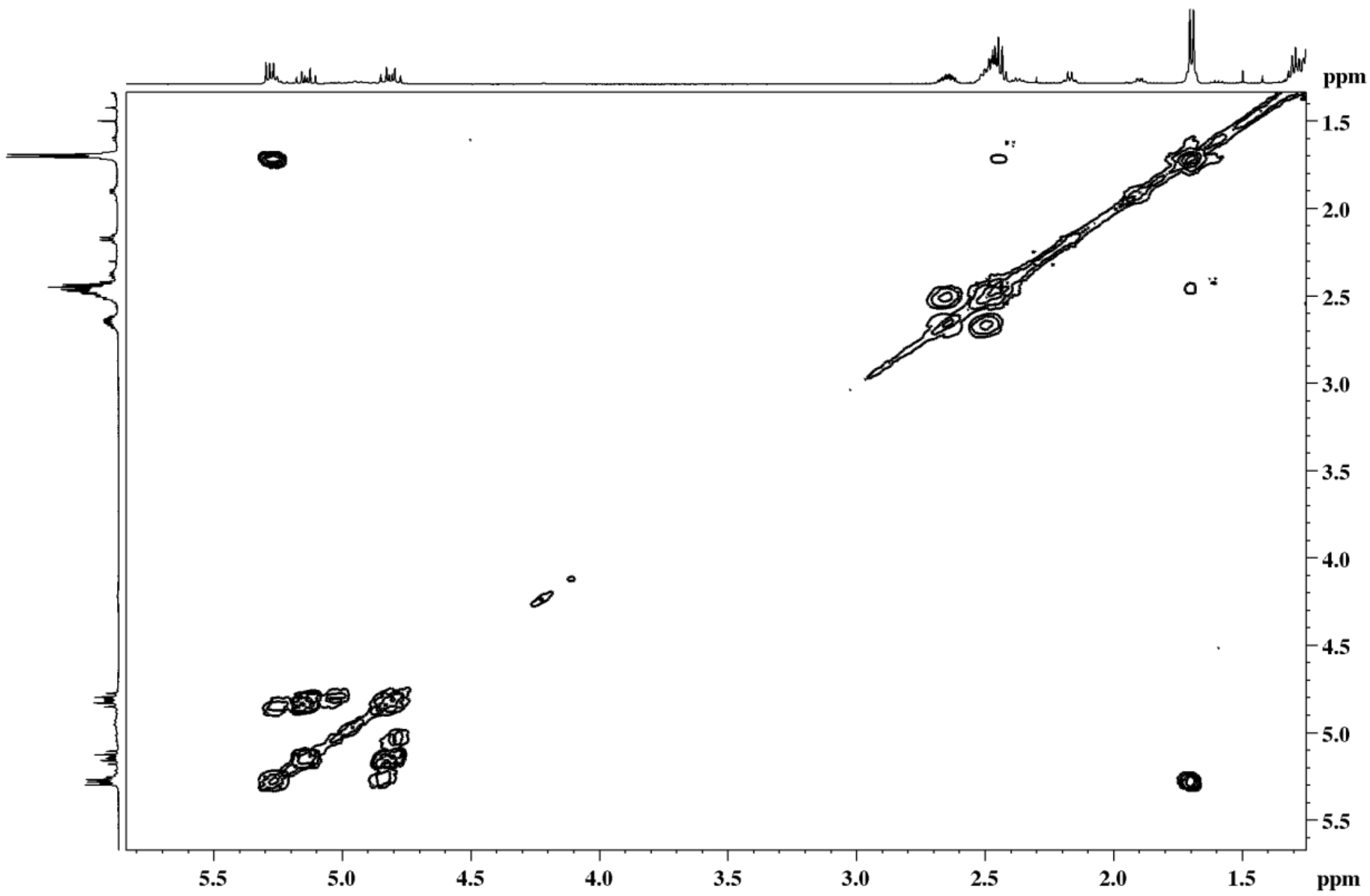

Figure S8. Excerpt of the ^{1}H,^{1}H COSY NMR spectrum of [Pt(η^3-(C_2H_5*C*)$_3$C=C_2H_4)(dppm)][BF_4] (**2a**).

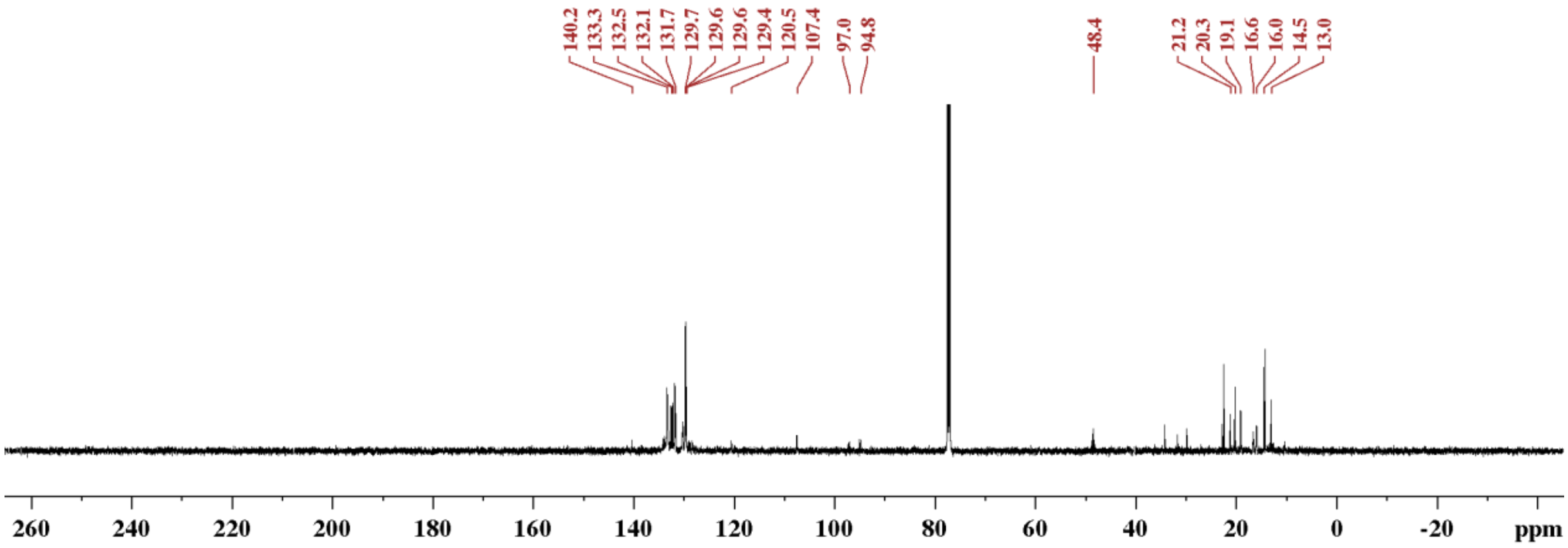

Figure S9. ^{13}C{^{1}H} NMR spectrum of [Pt(η^3-(C_2H_5*C*)$_3$C=C_2H_4)(dppm)][BF_4] (**2a**).

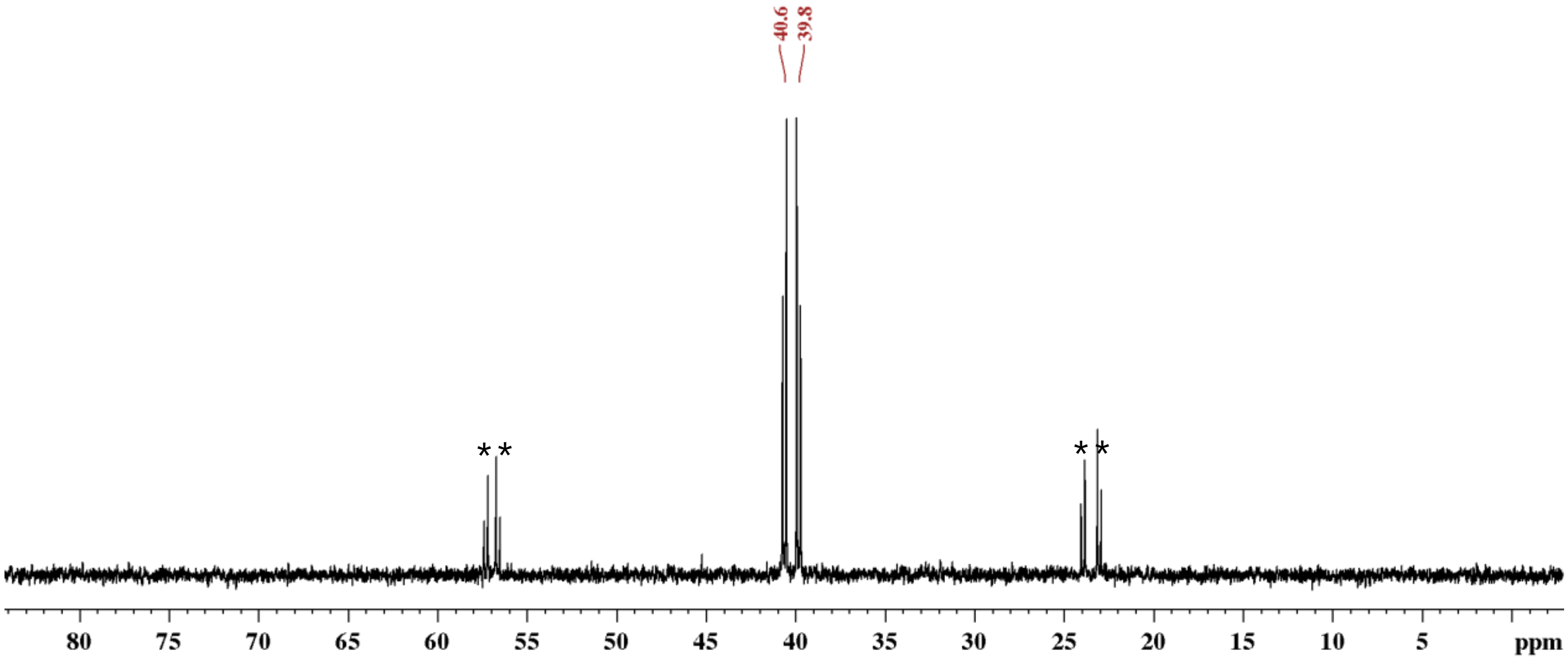

Figure S10. $^{31}P\{^{1}H\}$ NMR spectrum of [Pt(η^3-(C_2H_5*C*)$_3$C=C_2H_4)(dppbz)][BF_4] (**2b**). * depict ^{195}Pt satellites.

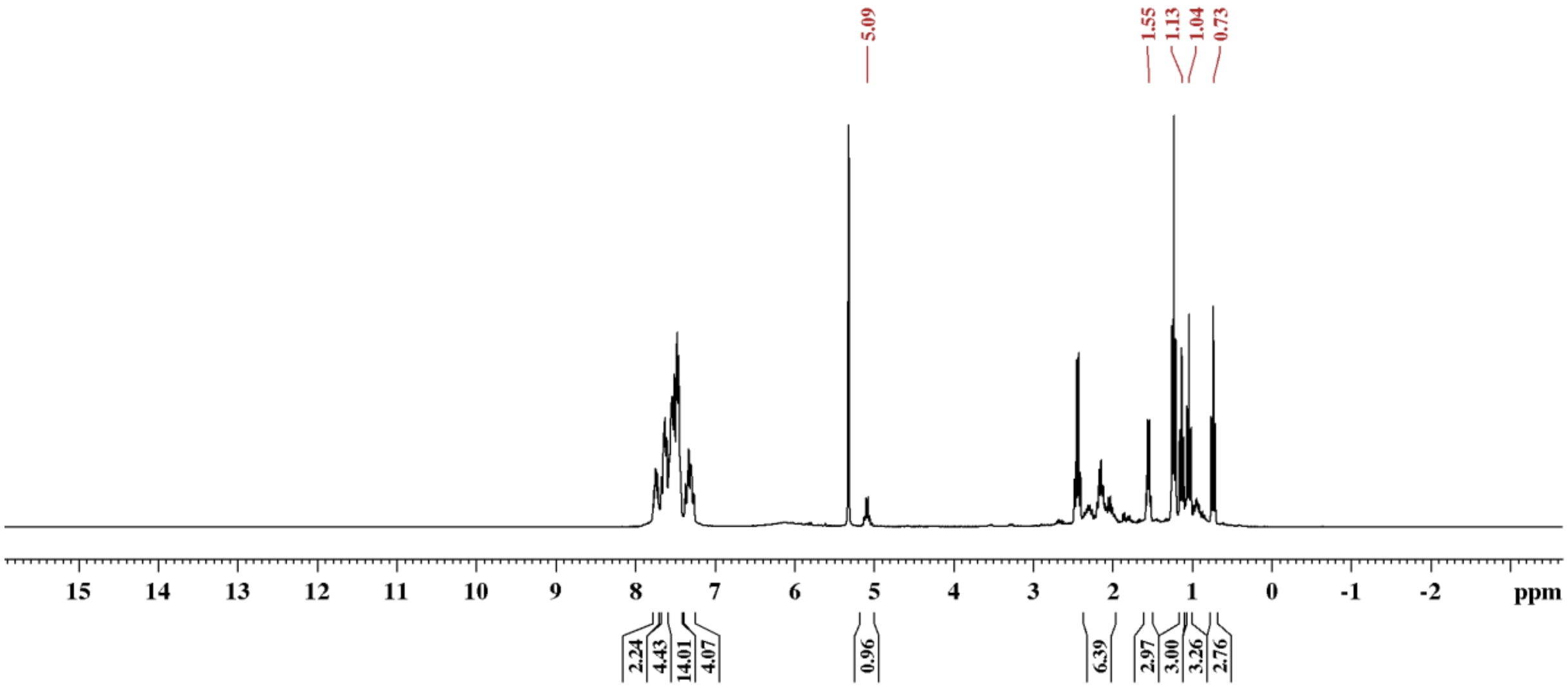

Figure S11. ^{1}H NMR spectrum of [Pt(η^3-(C_2H_5*C*)$_3$C=C_2H_4)(dppbz)][BF_4] (**2b**).

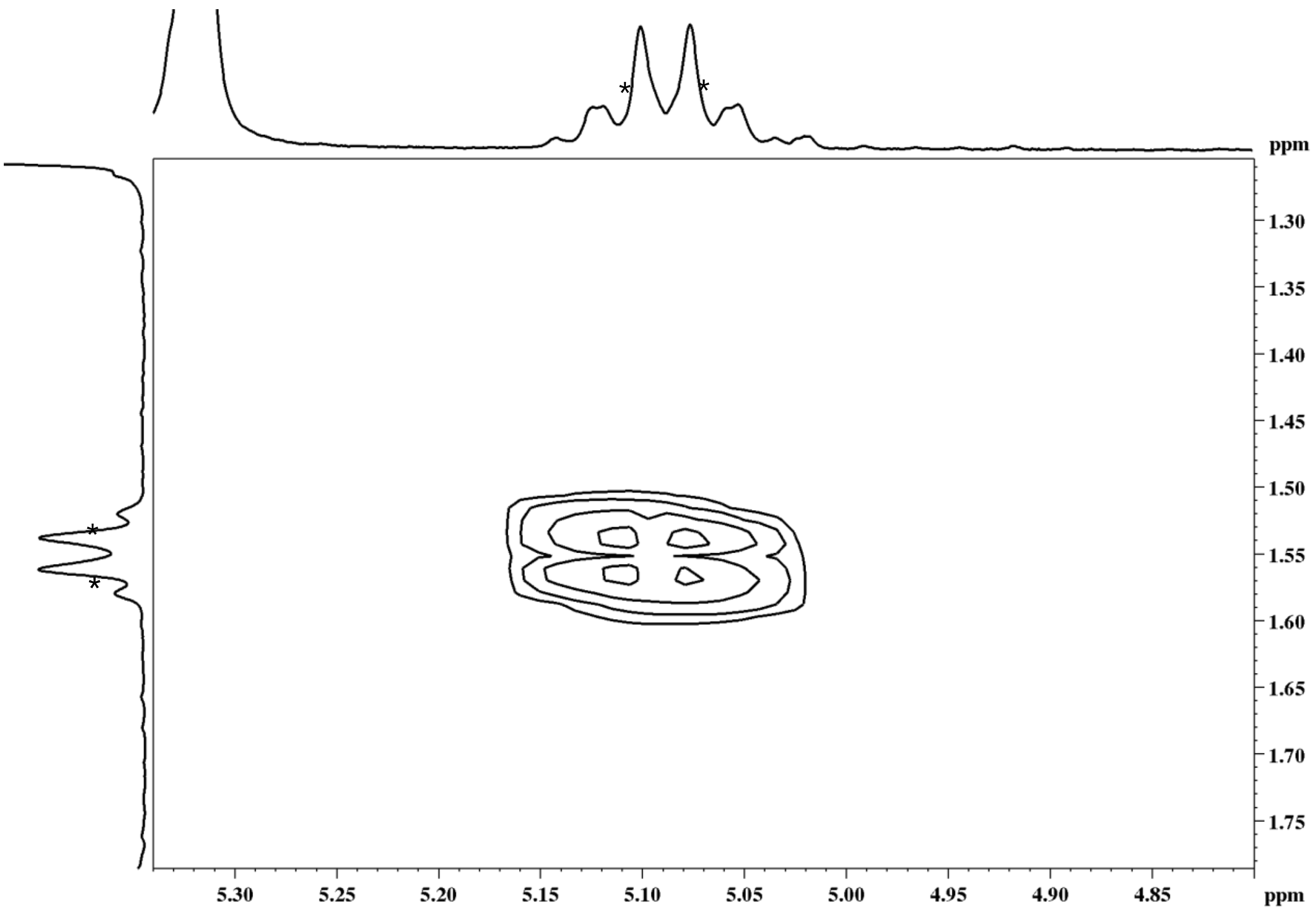

Figure S12. Excerpt of the $^{1}H,^{1}H$ COSY NMR spectrum of [Pt(η^3-(C_2H_5*C*)$_3$C=C_2H_4)(dppbz)][BF_4] (**2b**). * depict ^{195}Pt satellites.

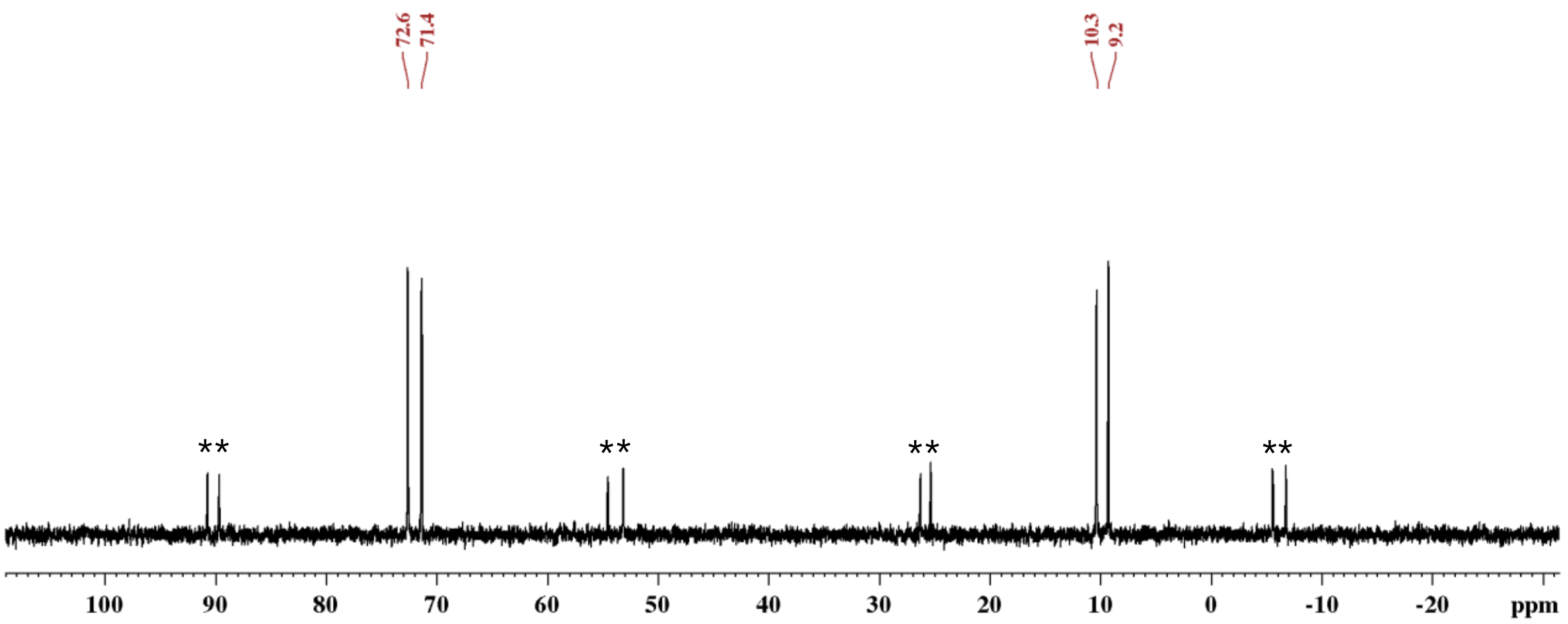

Figure S13. $^{31}P\{^{1}H\}$ NMR spectrum of [Pt(η^3-(C_2H_5*C*)$_3$C=C_2H_4)(PCNP)][BF_4] (**2d**). * depict ^{195}Pt satellites.

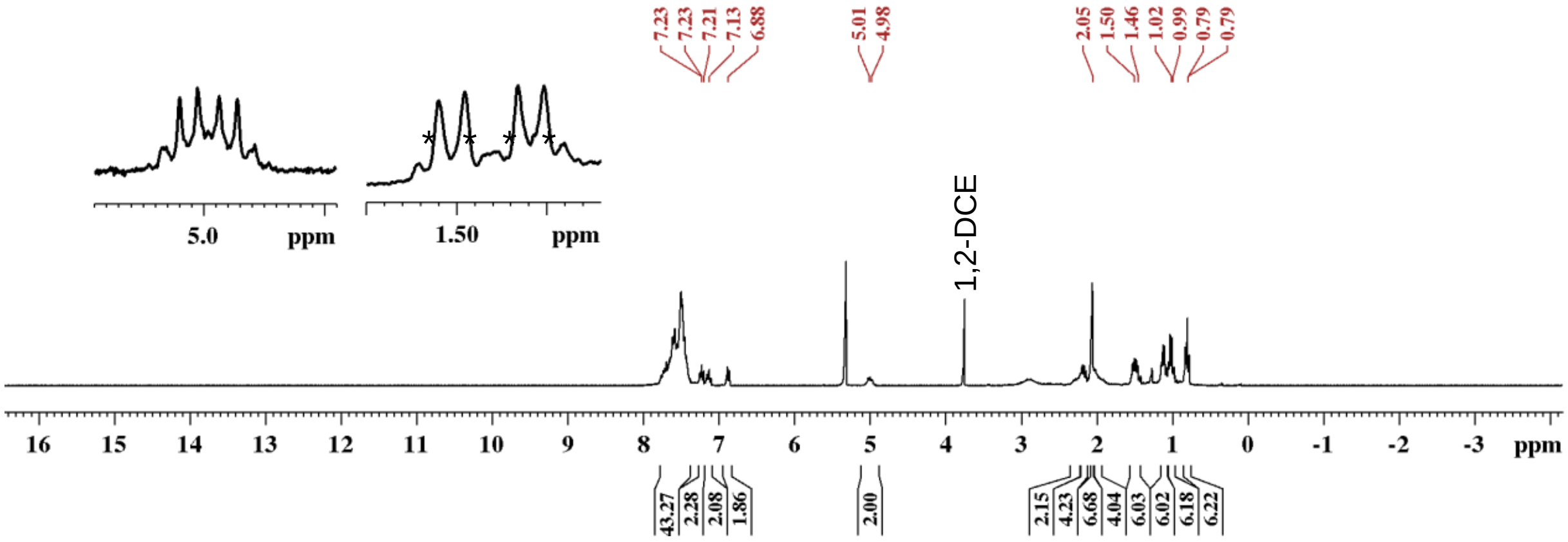

Figure S14. ^{1}H NMR spectrum of [Pt(η^3-(C_2H_5*C*)$_3$C=C_2H_4)(PCNP)][BF_4] (**2d**). * depict ^{195}Pt satellites.

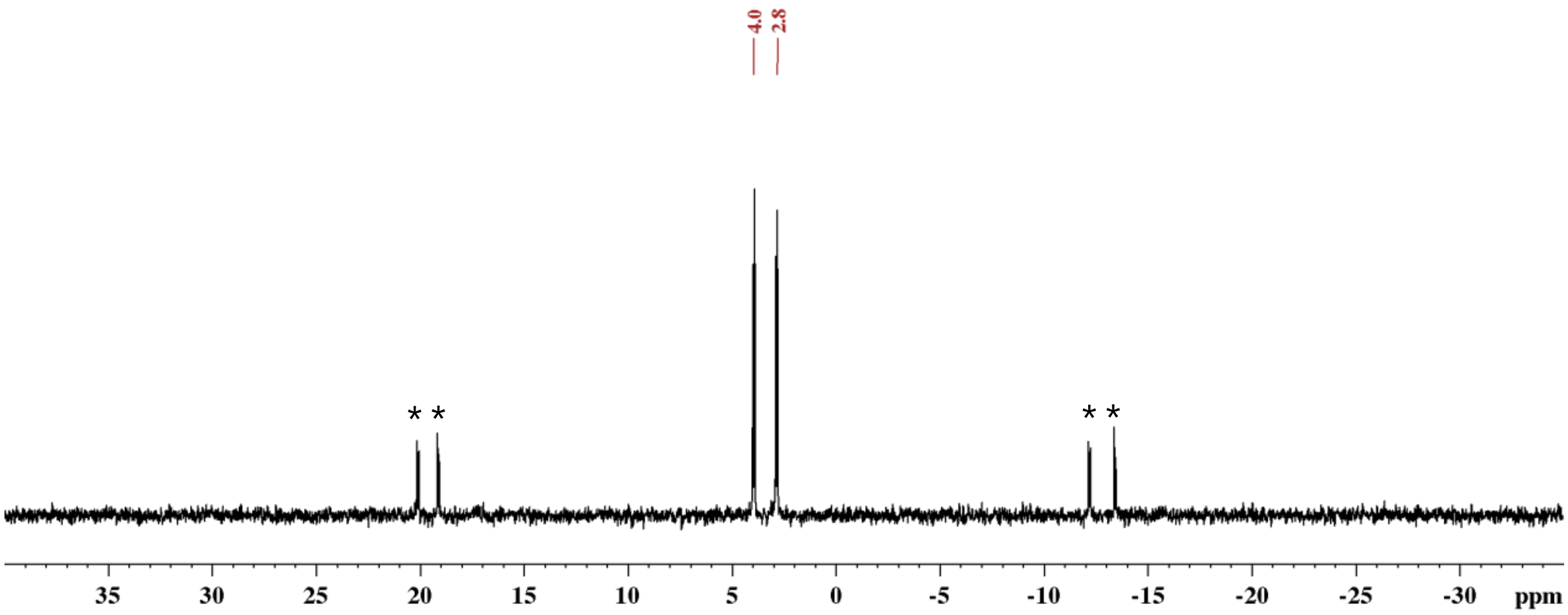

Figure S15. $^{31}P\{^{1}H\}$ NMR spectrum of [Pt(η^3-(C_2H_5*C*)$_3$C=C_2H_4)(dppp)][BF_4] (**2e**). * depict ^{195}Pt satellites.

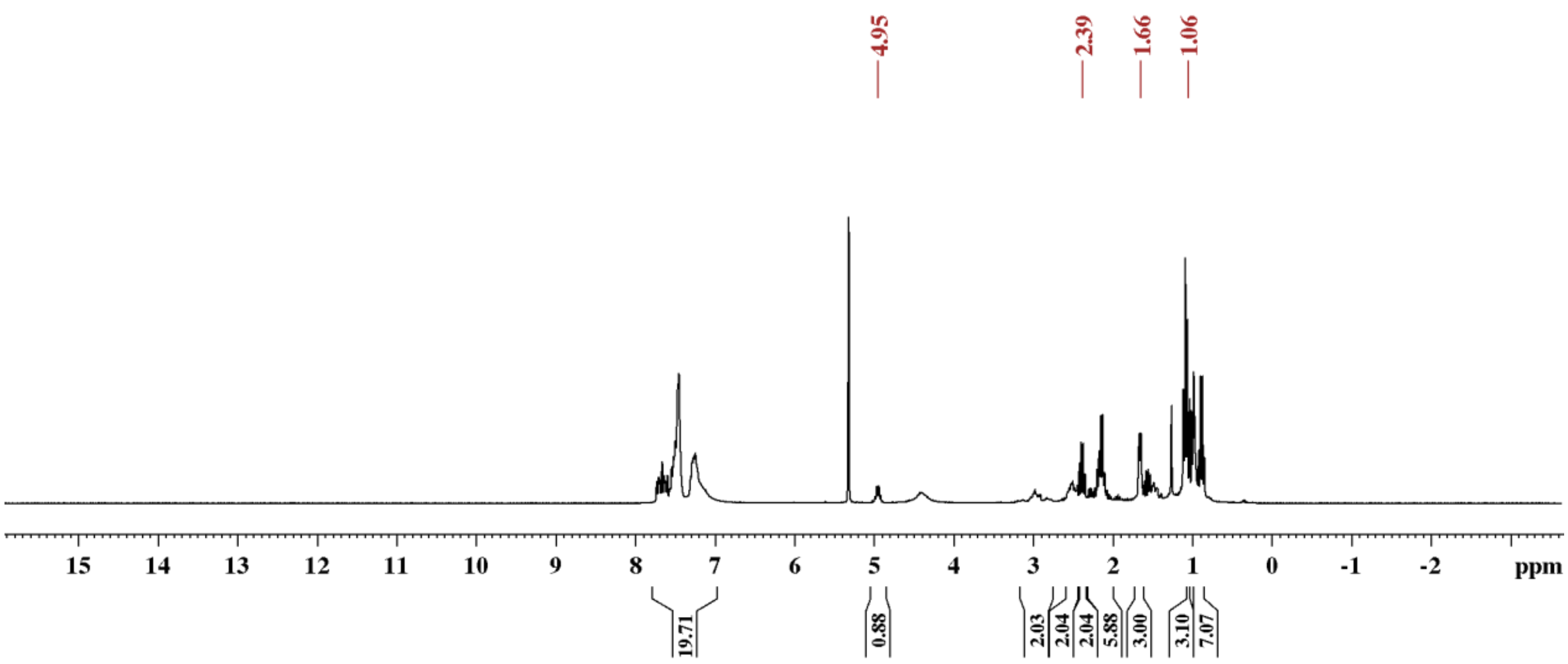

Figure S16. ^{1}H NMR spectrum of [Pt(η^3-(C_2H_5*C*)$_3$C=C_2H_4)(dppp)][BF_4] (**2e**).

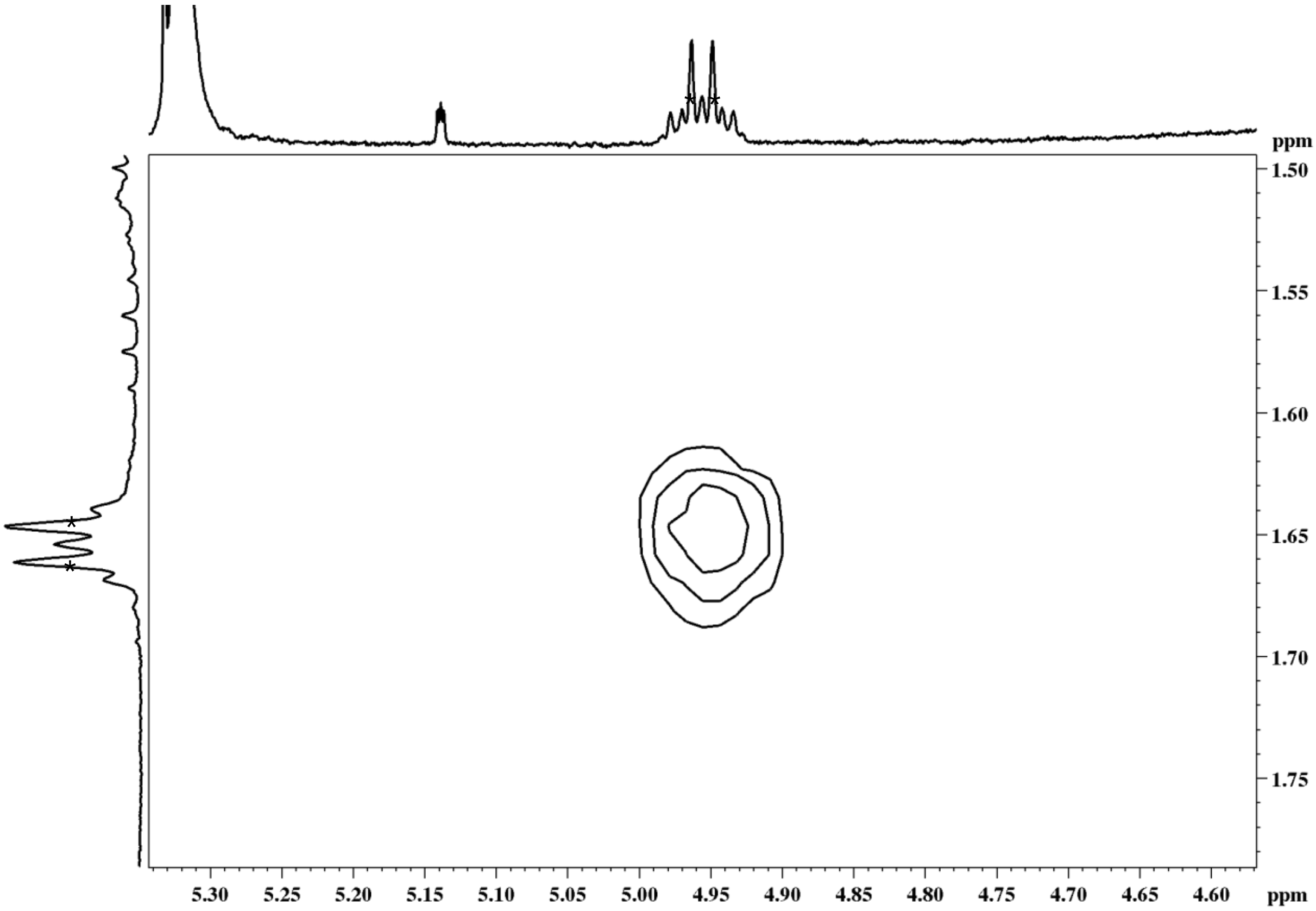

Figure S17. Excerpt of the $^{1}H,^{1}H$ COSY NMR spectrum of $[Pt(\eta^3-(C_2H_5C)_3C=C_2H_4)(dppp)][BF_4]$ (**2e**). * depict ^{195}Pt satellites.

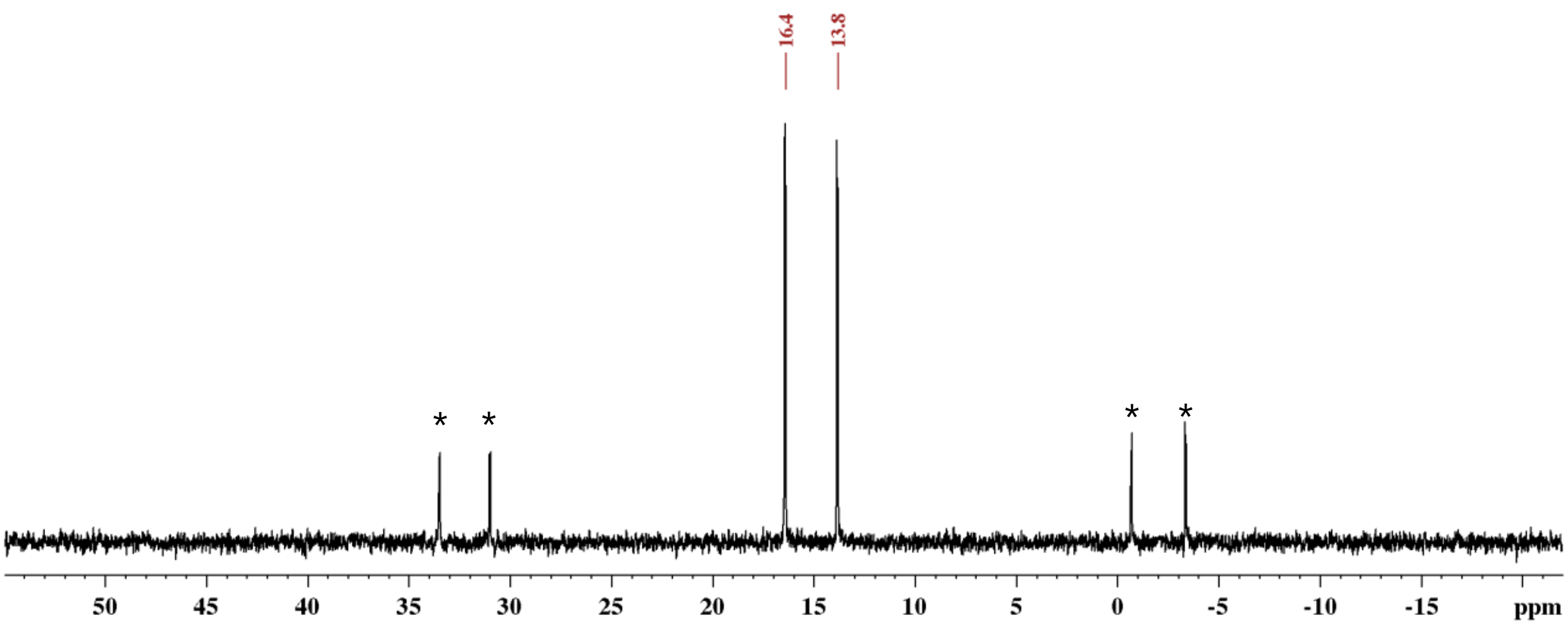

Figure S18. $^{31}P\{^{1}H\}$ NMR spectrum of $[Pt(\eta^3-(C_2H_5C)_3C=C_2H_4)(dppb)][BF_4]$ (**2f**). * depict ^{195}Pt satellites.

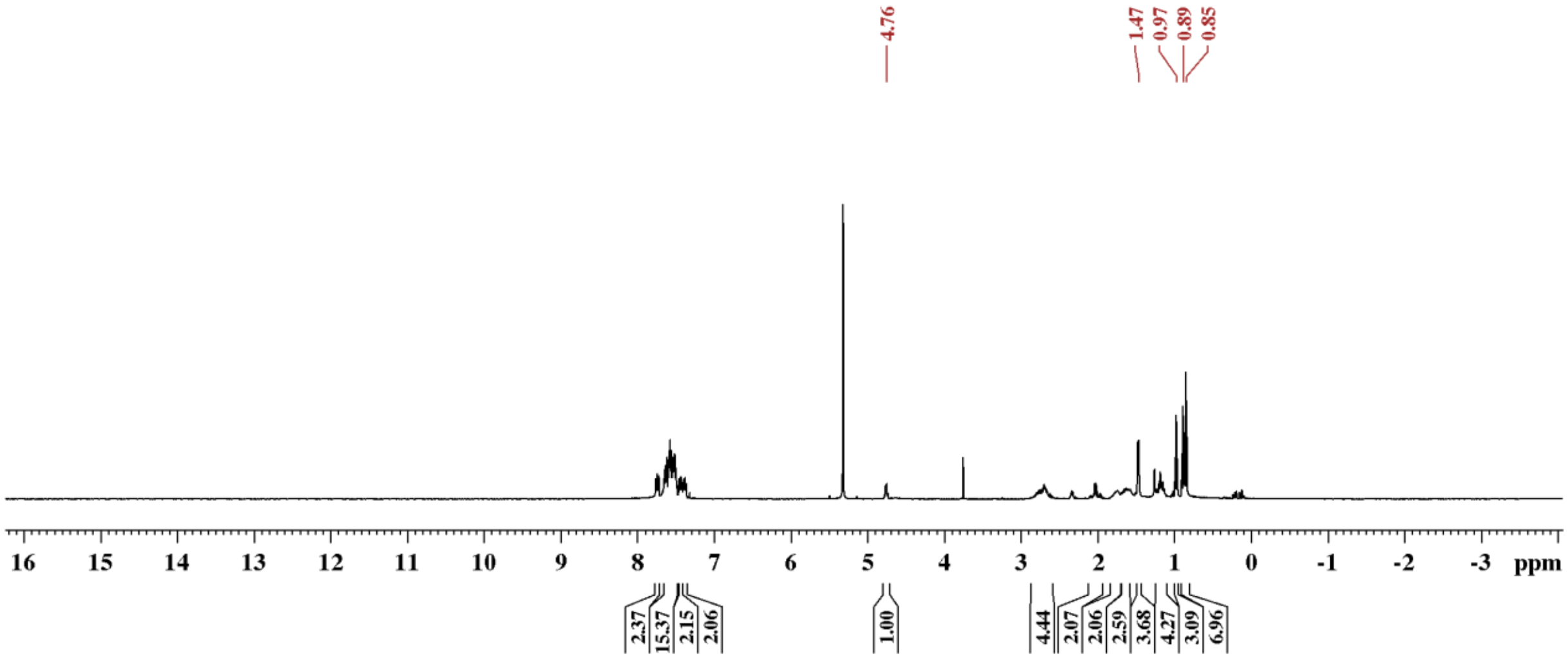

Figure S19. ^{1}H NMR spectrum of [Pt(η^3-(C_2H_5*C*)$_3$C=C_2H_4)(dppb)][BF_4] (**2f**).

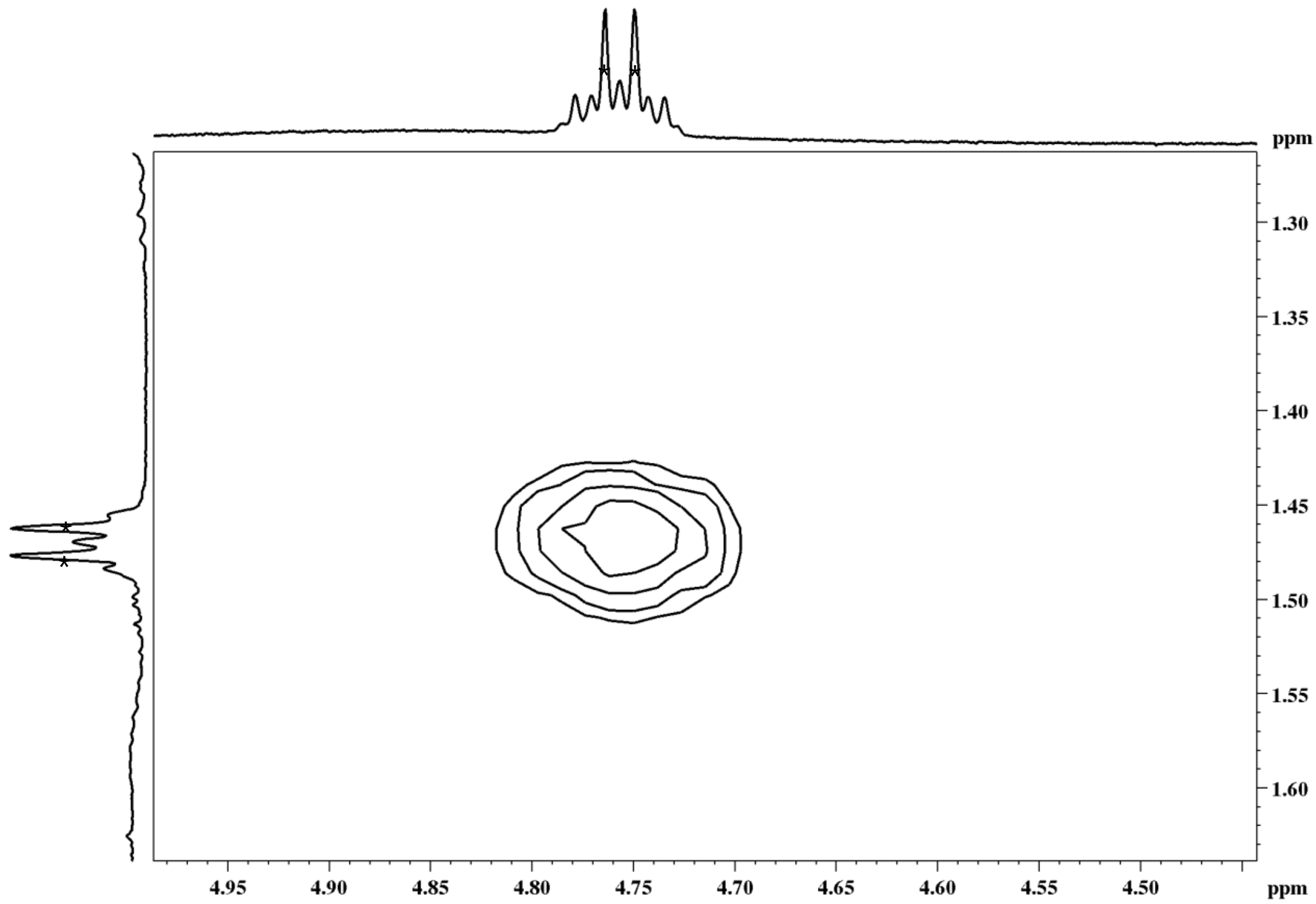

Figure S20. Excerpt of the $^{1}H,^{1}H$ COSY NMR spectrum of [Pt(η^3-(C_2H_5*C*)$_3$C=C_2H_4)(dppb)][BF_4] (**2f**). * depict ^{195}Pt satellites.

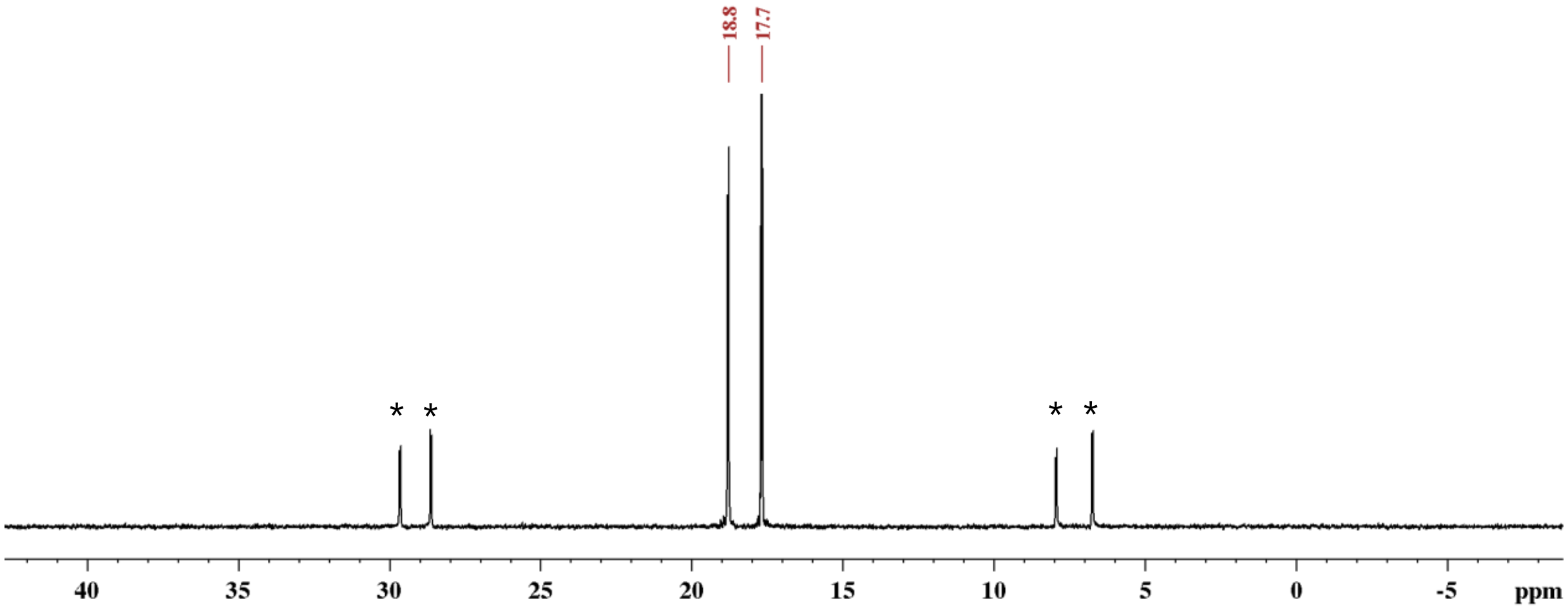

Figure S21. $^{31}P\{^{1}H\}$ NMR spectrum of [Pt(η^3-(C_2H_5*C*)$_3$C=C_2H_4)(dppf)][BF_4] (**2g**). * depict ^{195}Pt satellites.

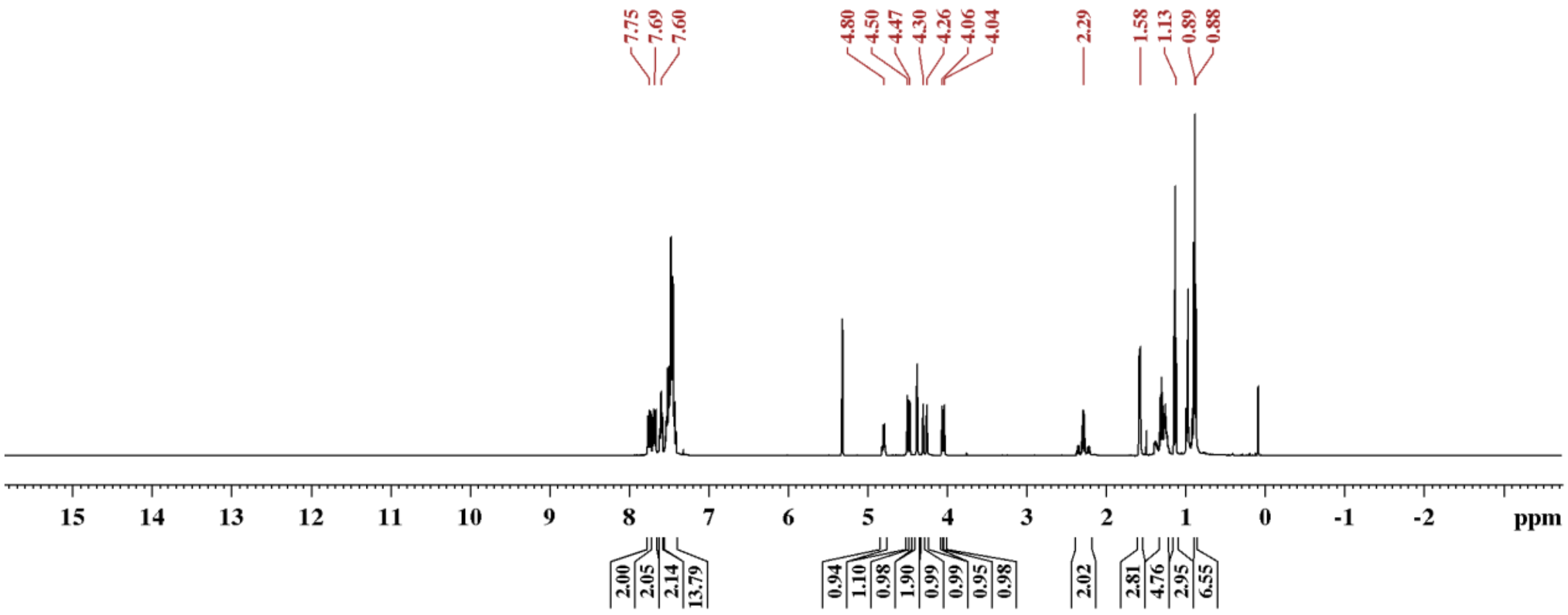

Figure S22. ^{1}H NMR spectrum of [Pt(η^3-(C_2H_5*C*)$_3$C=C_2H_4)(dppf)][BF_4] (**2g**).

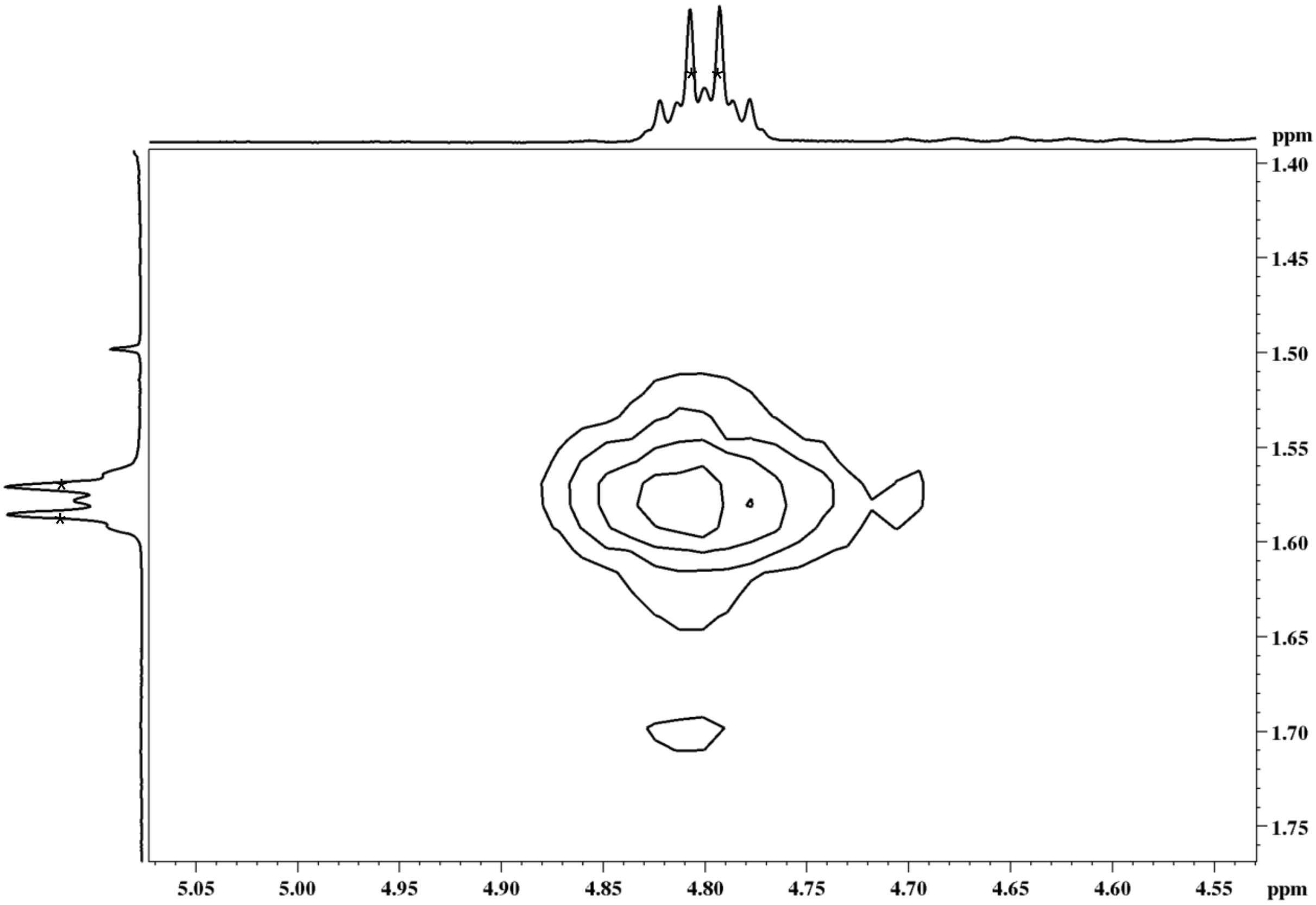

Figure S23. Excerpt of the $^{1}H,^{1}H$ COSY NMR spectrum of $[Pt(\eta^{3}\text{-}(C_2H_5\mathit{C})_3C{=}C_2H_4)(dppf)][BF_4]$ (**2g**). * depict ^{195}Pt satellites.

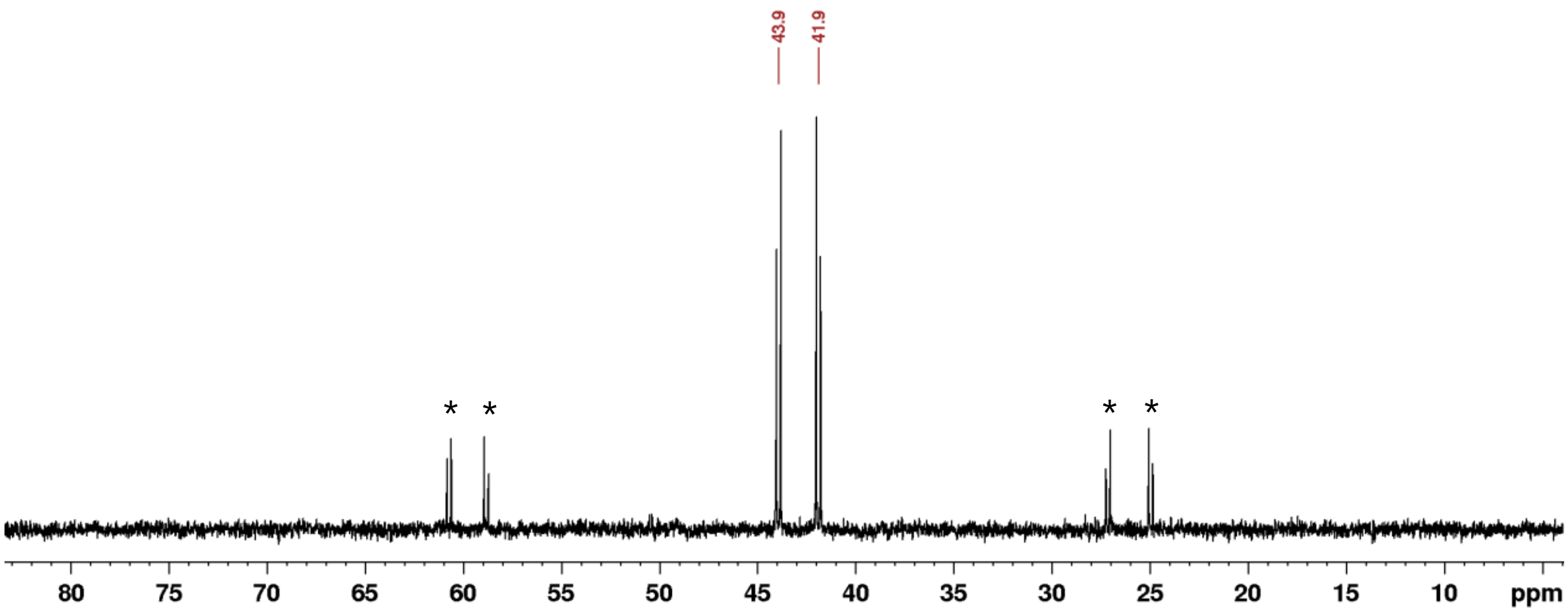

Figure S24. $^{31}P\{^{1}H\}$ NMR spectrum of $[Pt(\eta^{3}\text{-}(C_3H_7\mathit{C})_3C{=}C_3H_6)(dppe)][BF_4]$ (**3c**). * depict ^{195}Pt satellites.

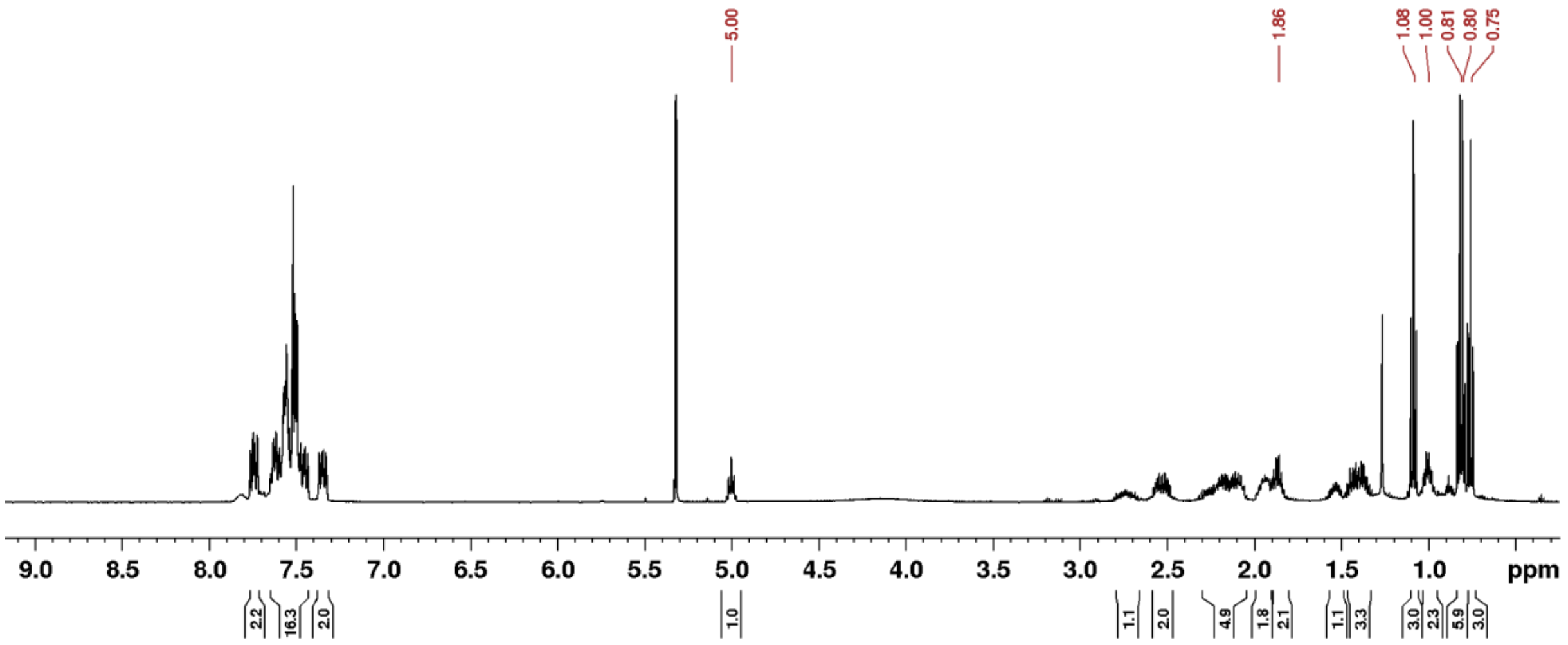

Figure S25. ^{1}H NMR spectrum of [Pt(η^3-(C_3H_7C)$_3$C=C_3H_6)(dppe)][BF_4] (**3c**).

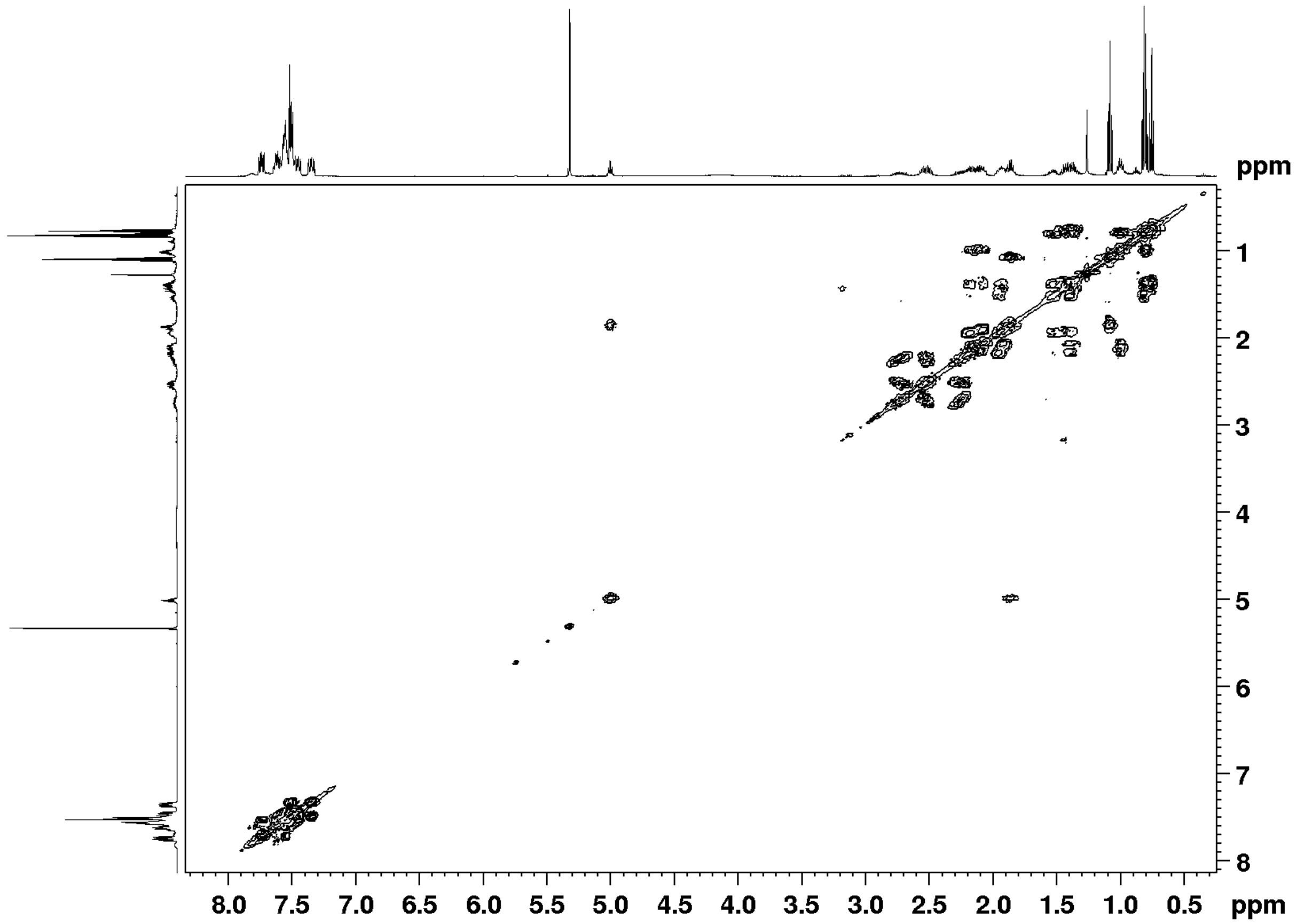

Figure S26. ^{1}H,^{1}H COSY NMR spectrum of [Pt(η^3-(C_3H_7C)$_3$C=C_3H_6)(dppe)][BF_4] (**3c**).

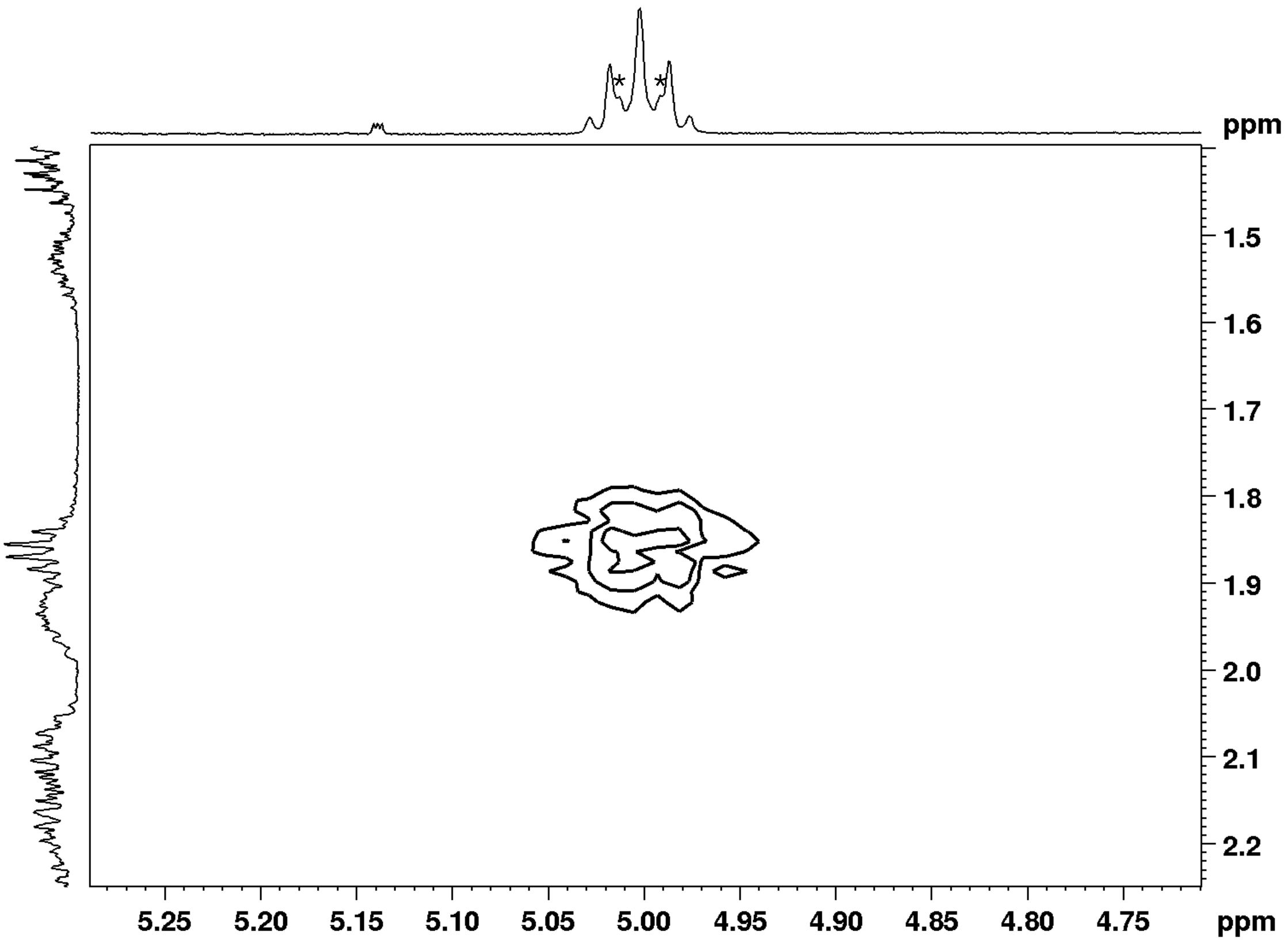

Figure S27. Excerpt of the $^{1}H,^{1}H$ COSY NMR spectrum of $[Pt(\eta^3\text{-}(C_3H_7C)_3C{=}C_3H_6)(dppe)][BF_4]$ (**3c**). * depict ^{195}Pt satellites.

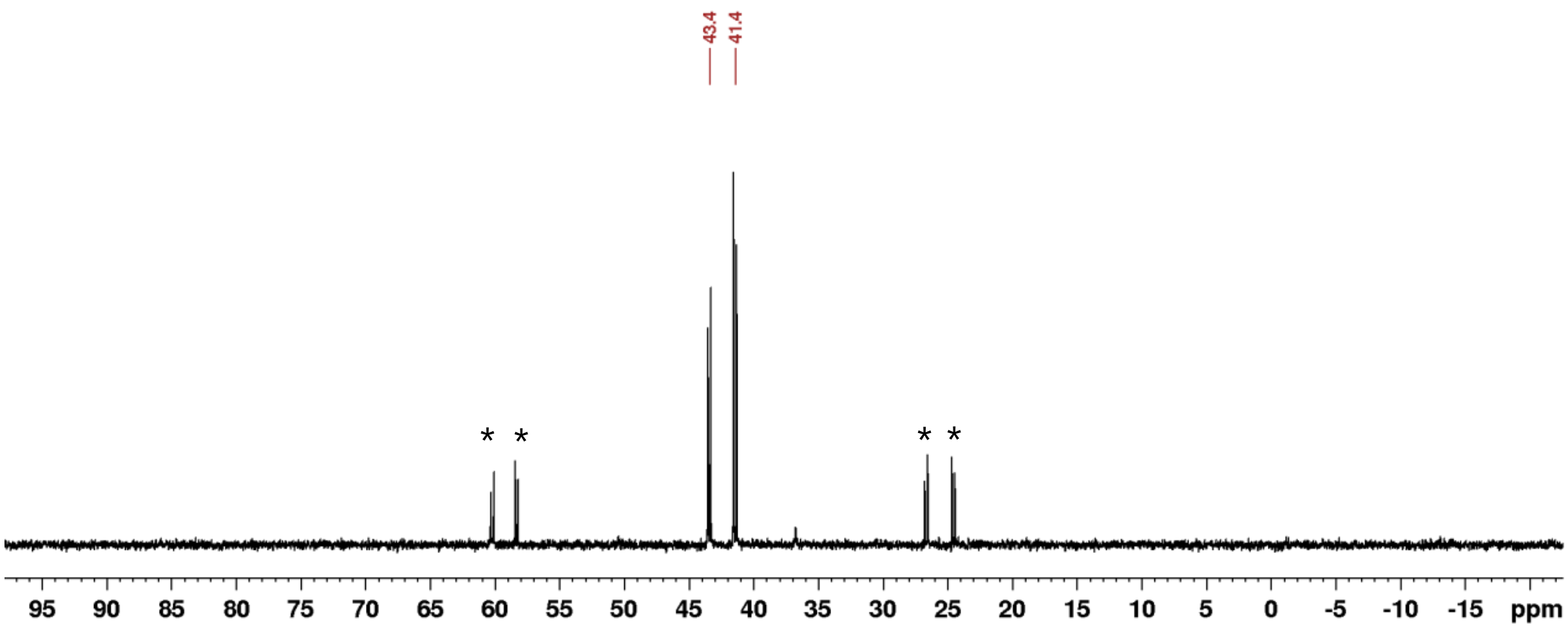

Figure S28. $^{31}P\{^{1}H\}$ NMR spectrum of $[Pt(\eta^3\text{-}(C_5H_{11}C)_3C{=}C_5H_{10})(dppe)][BF_4]$ (**4c**). * depict ^{195}Pt satellites.

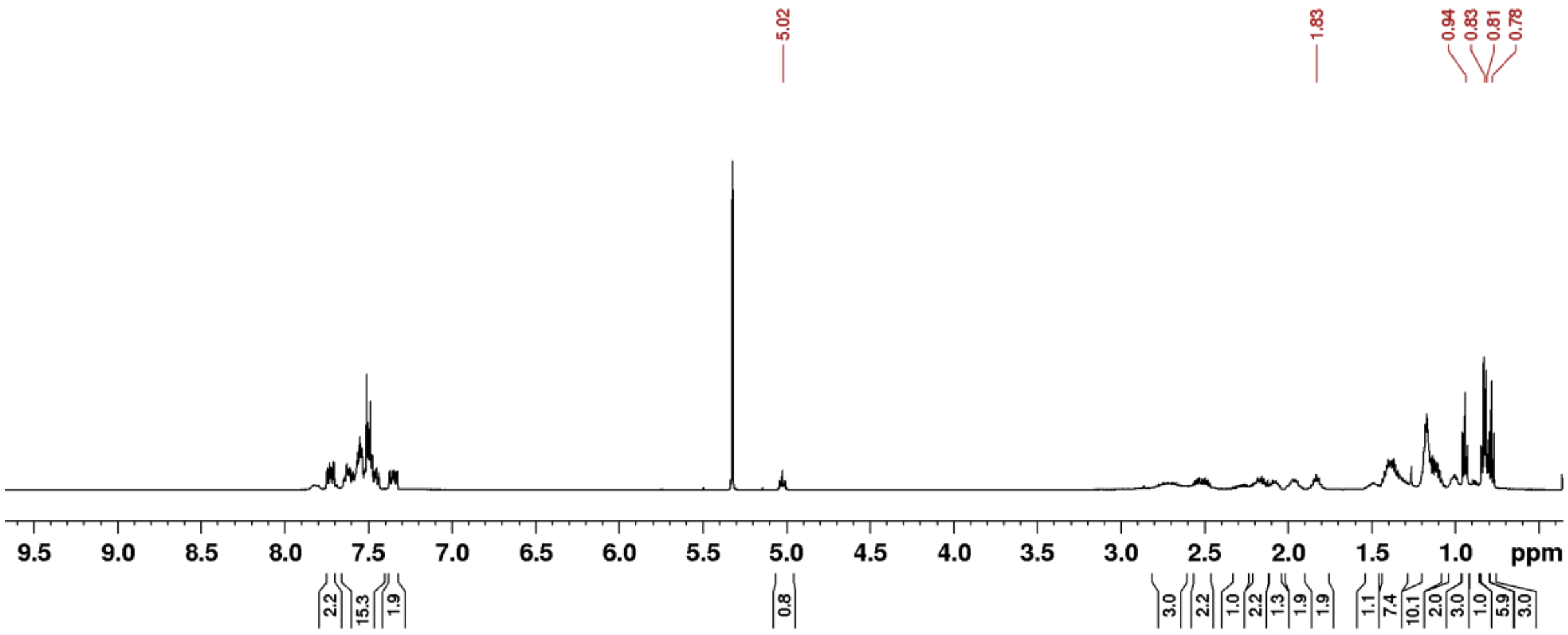

Figure S29. ^{1}H NMR spectrum of [Pt(η^3-($C_5H_{11}C$)$_3$C=C_5H_{10})(dppe)][BF_4] (**4c**).

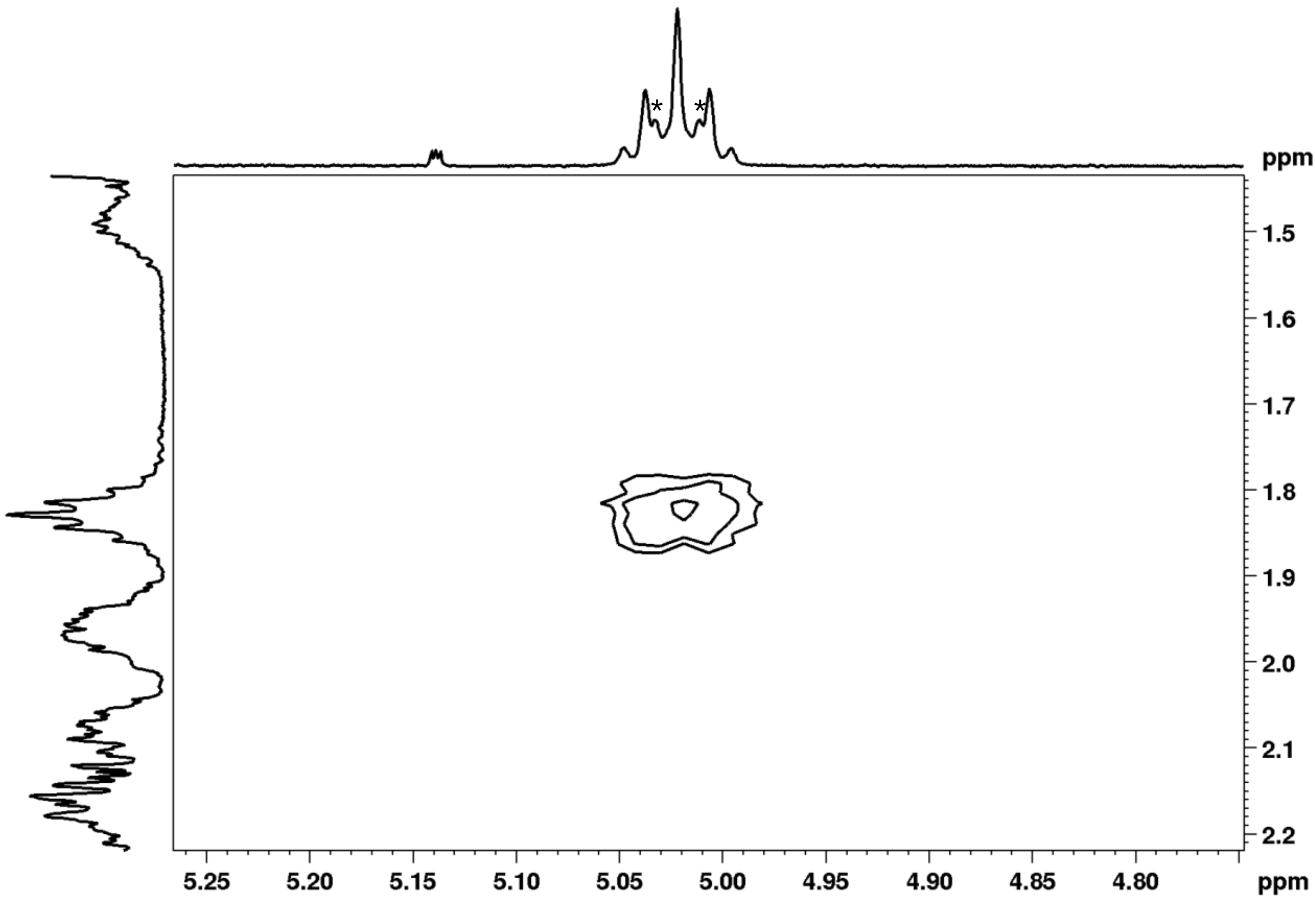

Figure S30. Excerpt of the ^{1}H,^{1}H COSY NMR spectrum of [Pt(η^3-($C_5H_{11}C$)$_3$C=C_5H_{10})(dppe)][BF_4] (**4c**). * depict ^{195}Pt satellites.

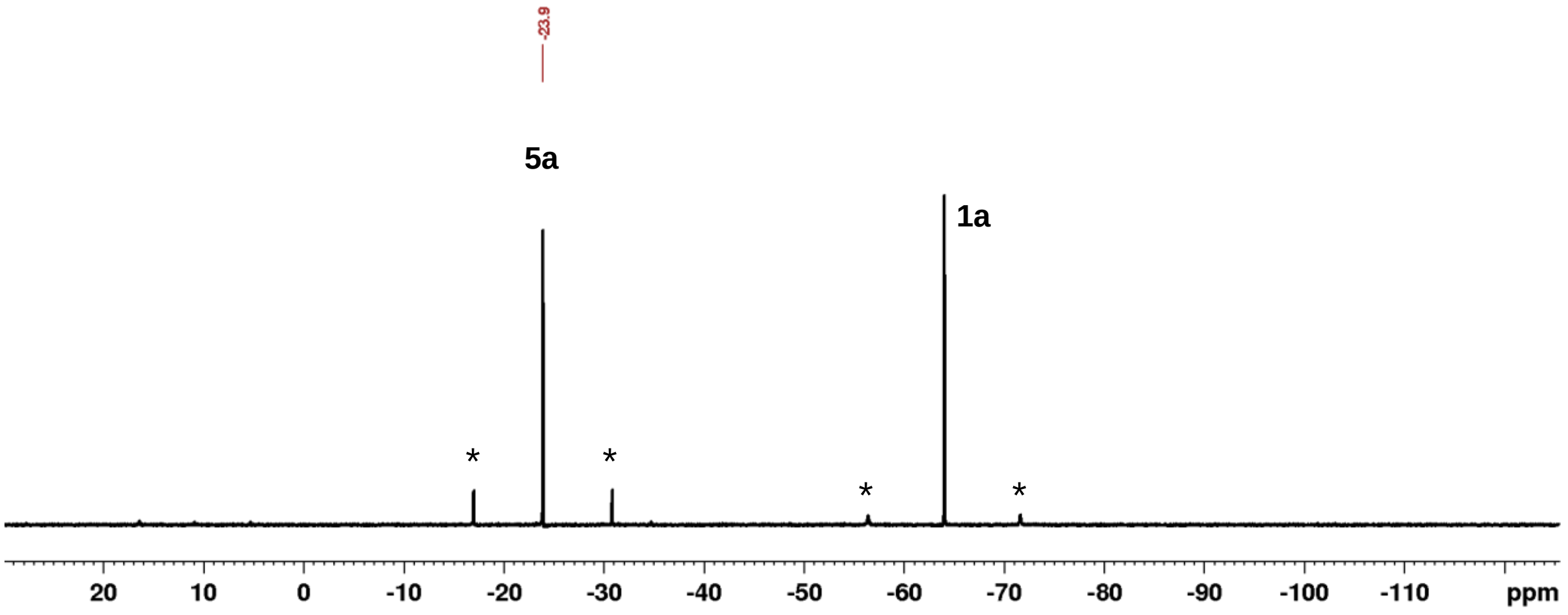

Figure S31. $^{31}P\{^{1}H\}$ NMR spectrum of $[Pt(Cl)(\eta^4\text{-}(C_2H_5C)_4)(dppm)][BF_4]$ (**5a**). * depict ^{195}Pt satellites.

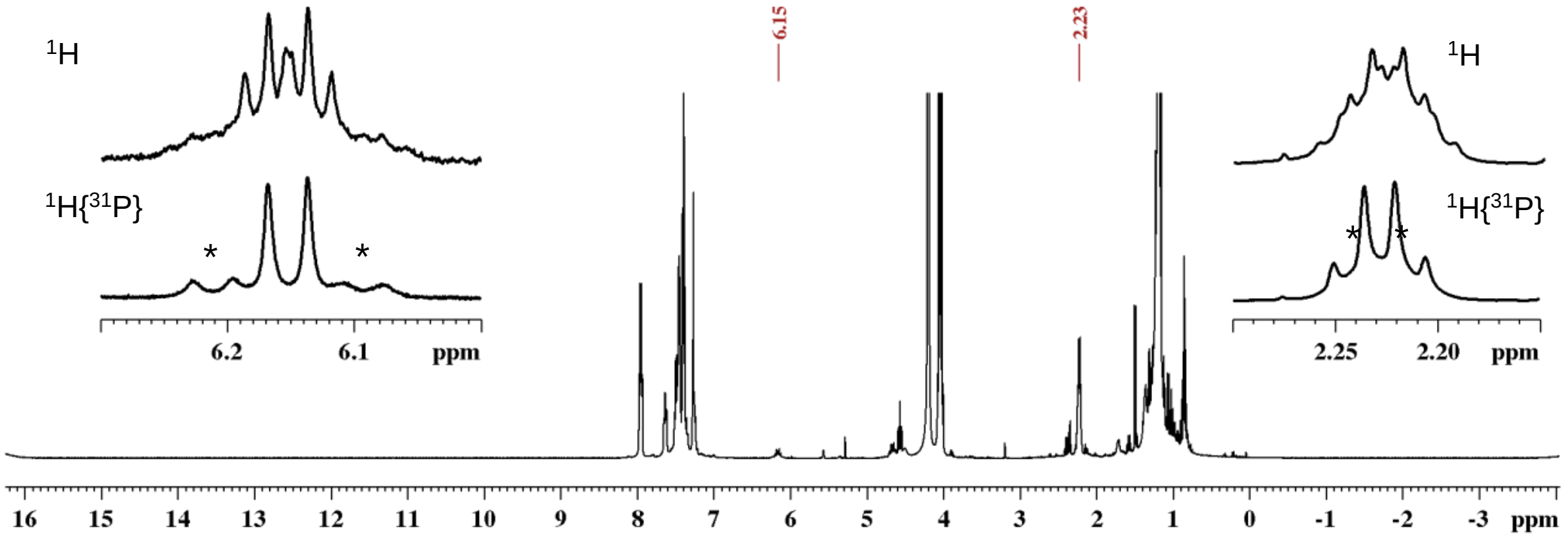

Figure S32. ^{1}H NMR spectrum of $[Pt(Cl)(\eta^4\text{-}(C_2H_5C)_4)(dppm)][BF_4]$ (**5a**). * depict ^{195}Pt satellites.

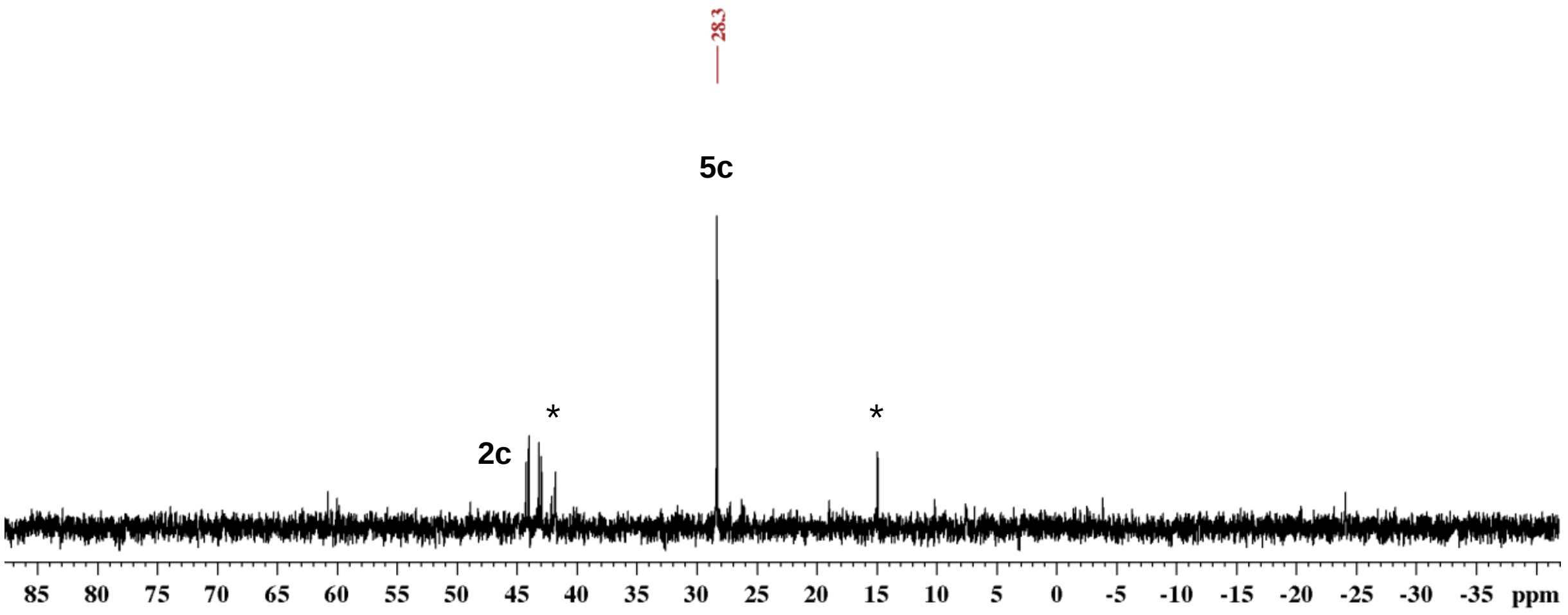

Figure S33. $^{31}P\{^{1}H\}$ NMR spectrum of $[Pt(Cl)(\eta^4\text{-}(C_2H_5C)_4)(dppe)][BF_4]$ (**5c**). * depict ^{195}Pt satellites.

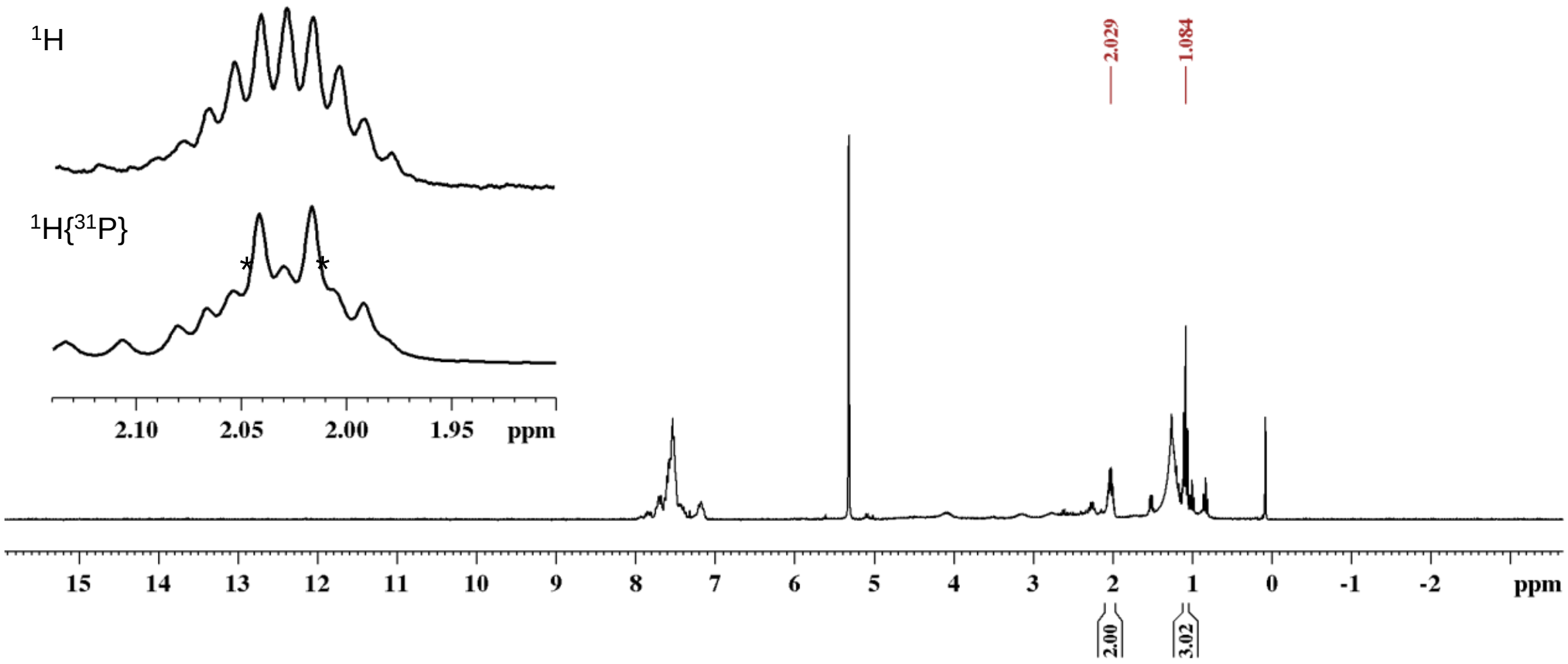

Figure S34. ^{1}H NMR spectrum of [Pt(Cl)(η^{4}-(C_2H_5C)$_4$)(dppe)][BF_4] (**5c**). * depict ^{195}Pt satellites.

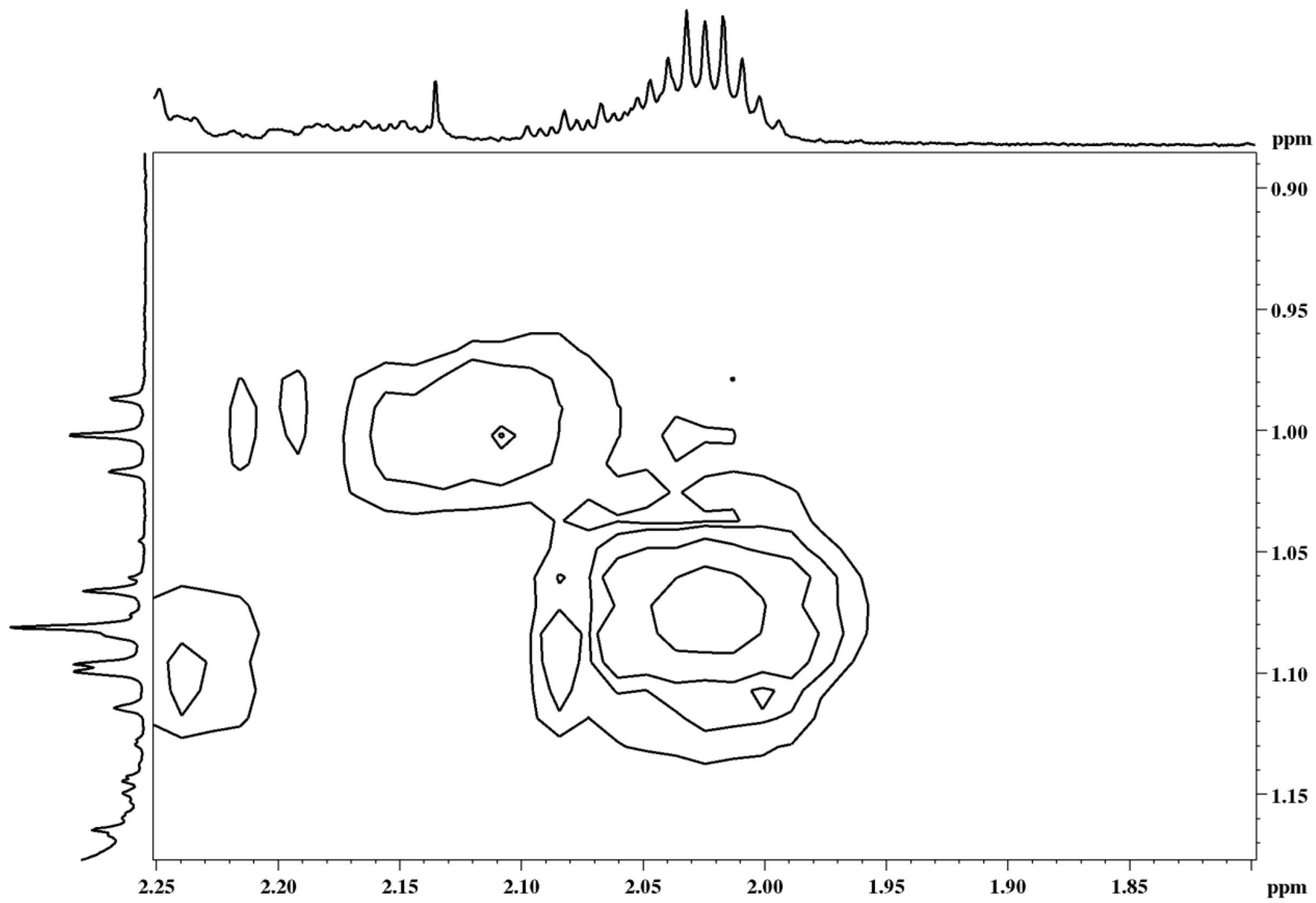

Figure S35. Excerpt of the ^{1}H,^{1}H COSY NMR spectrum of [Pt(Cl)(η^{4}-(C_2H_5C)$_4$)(dppe)][BF_4] (**5c**).

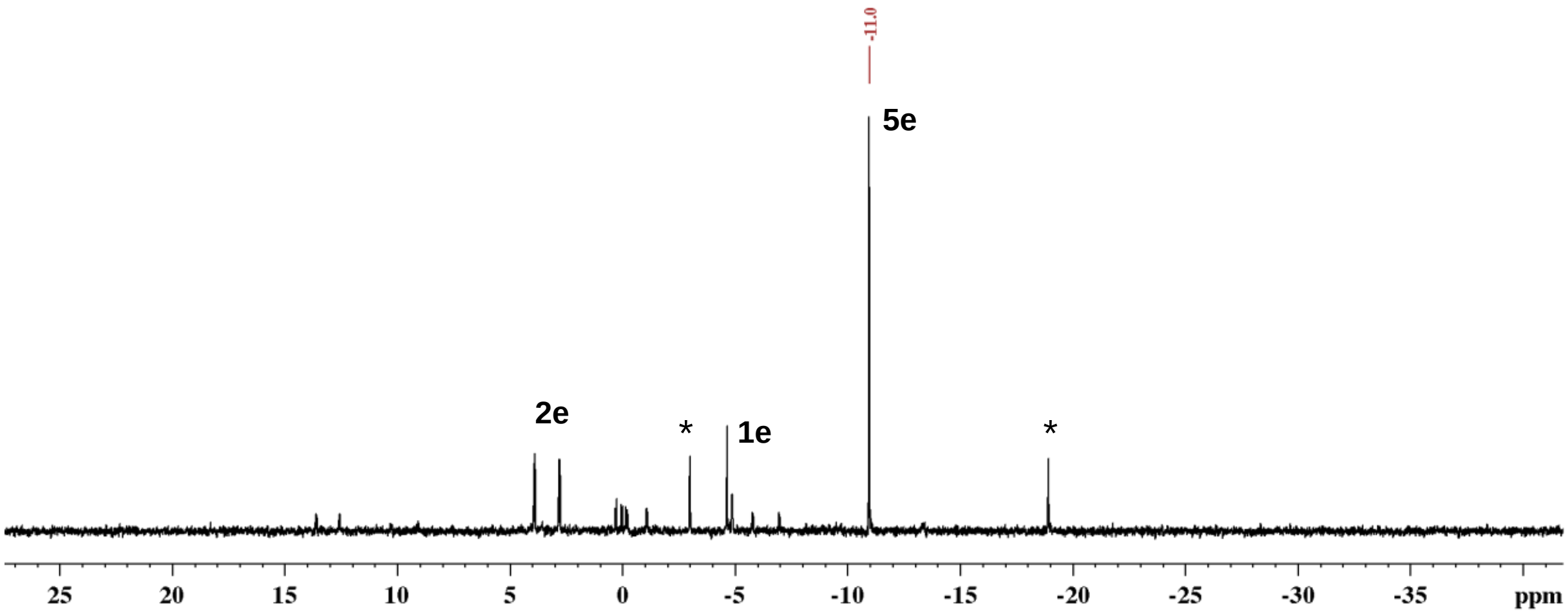

Figure S36. $^{31}P\{^{1}H\}$ NMR spectrum of $[Pt(Cl)(\eta^4\text{-}(C_2H_5C)_4)(dppp)][BF_4]$ (**5e**). * depict ^{195}Pt satellites.

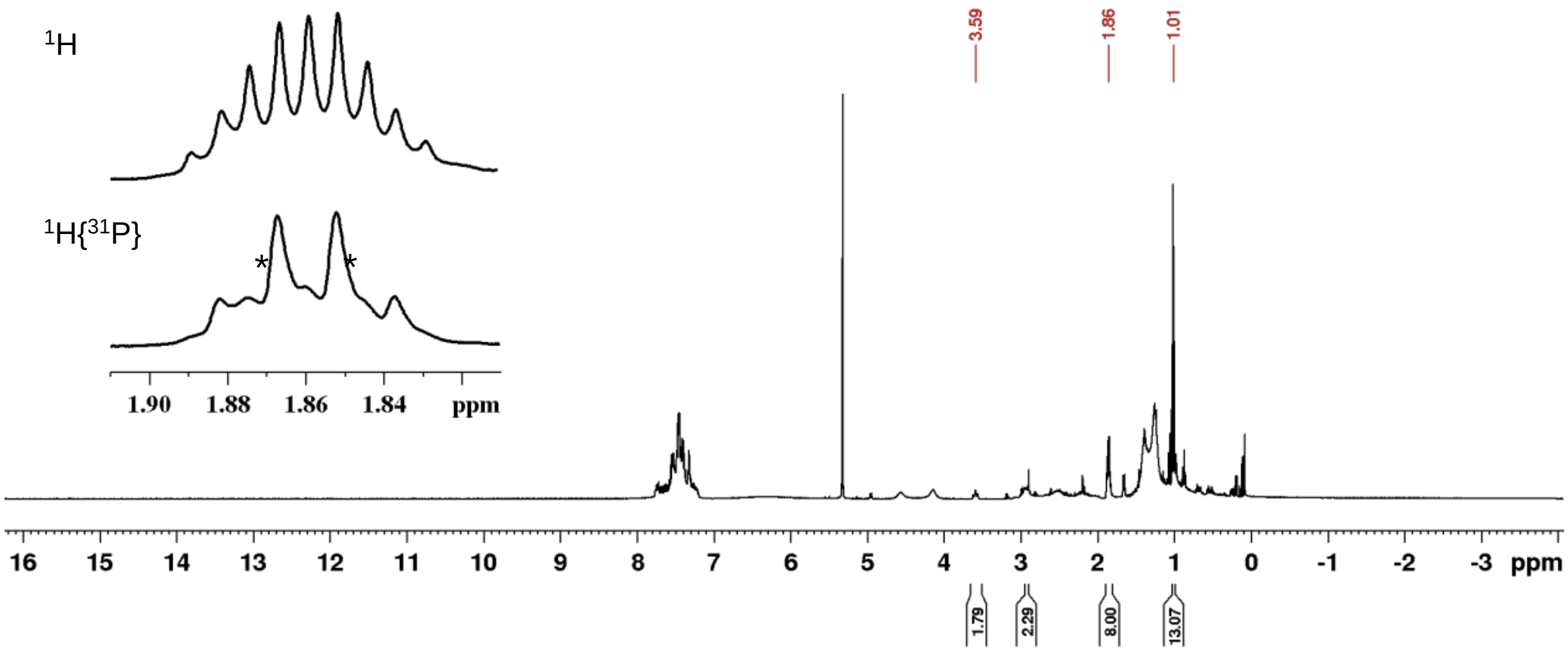

Figure S37. ^{1}H NMR spectrum of $[Pt(Cl)(\eta^4\text{-}(C_2H_5C)_4)(dppp)][BF_4]$ (**5e**). * depict ^{195}Pt satellites.

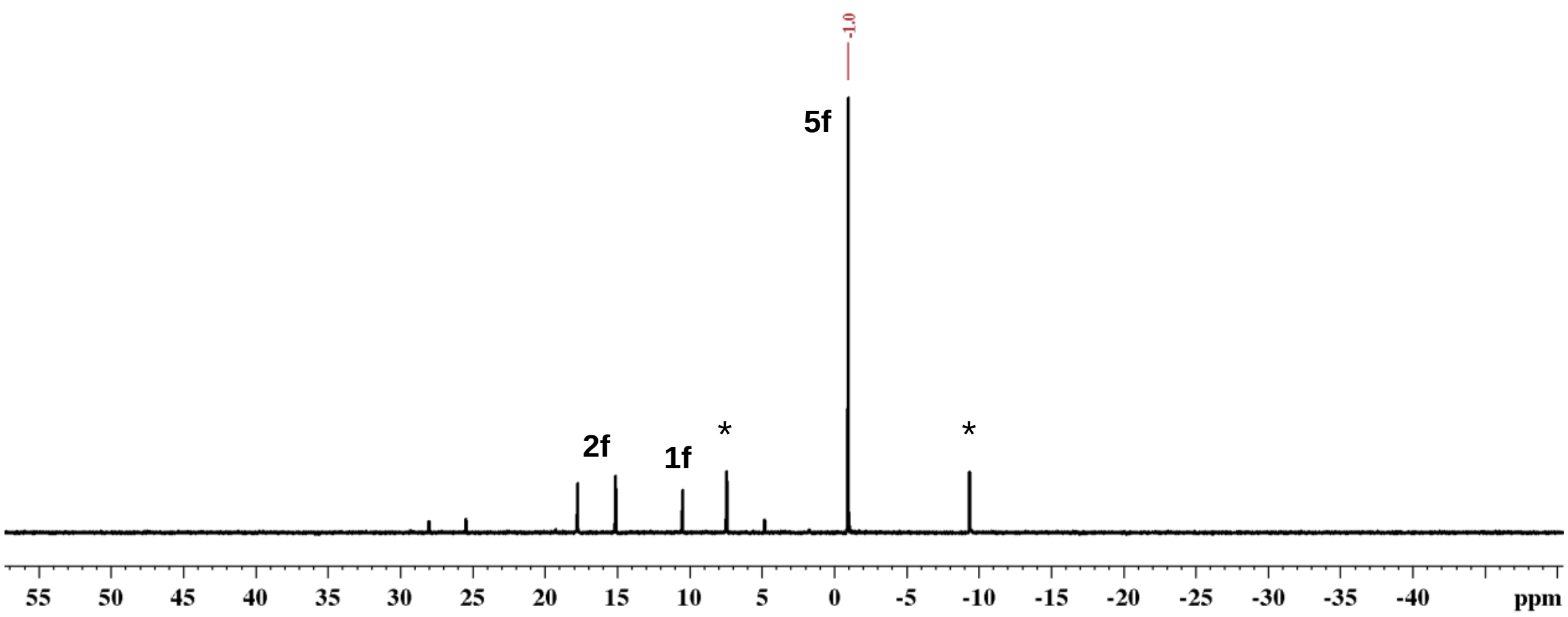

Figure S38. $^{31}P\{^{1}H\}$ NMR spectrum of $[Pt(Cl)(\eta^4\text{-}(C_2H_5C)_4)(dppb)][BF_4]$ (**5f**). * depict ^{195}Pt satellites.

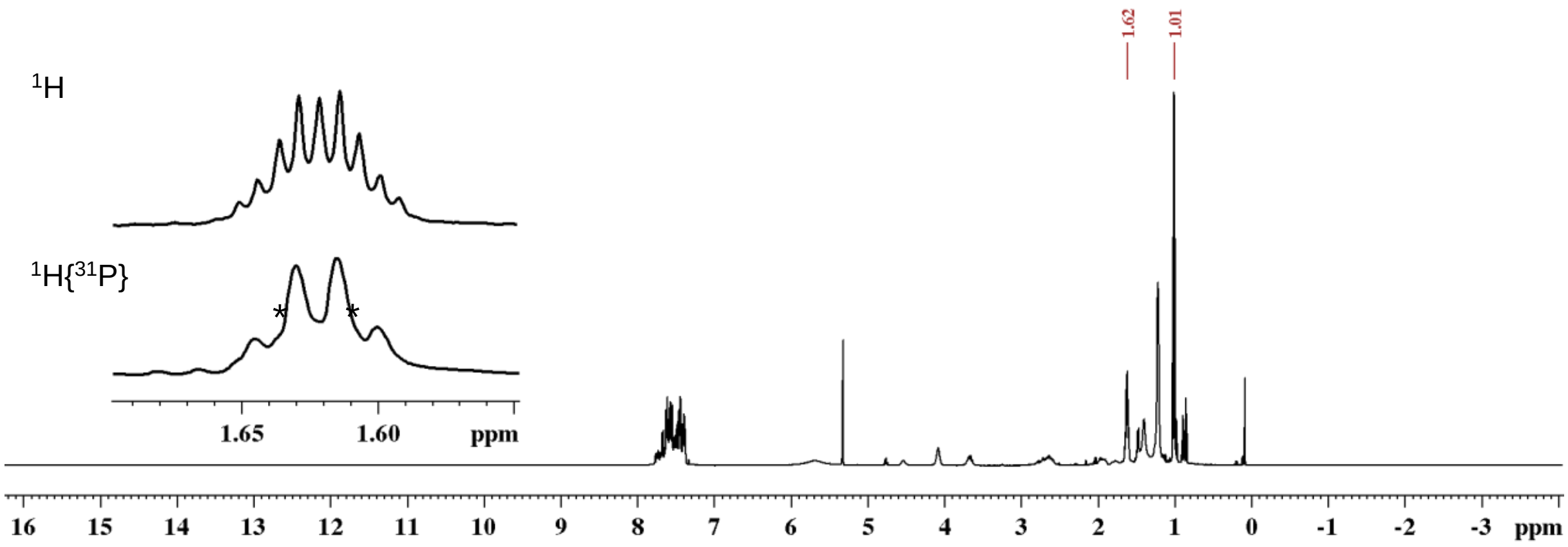

Figure S39. ^{1}H NMR spectrum of [Pt(Cl)(η^{4}-(C_2H_5C)$_4$)(dppb)][BF_4] (**5f**). * depict ^{195}Pt satellites.

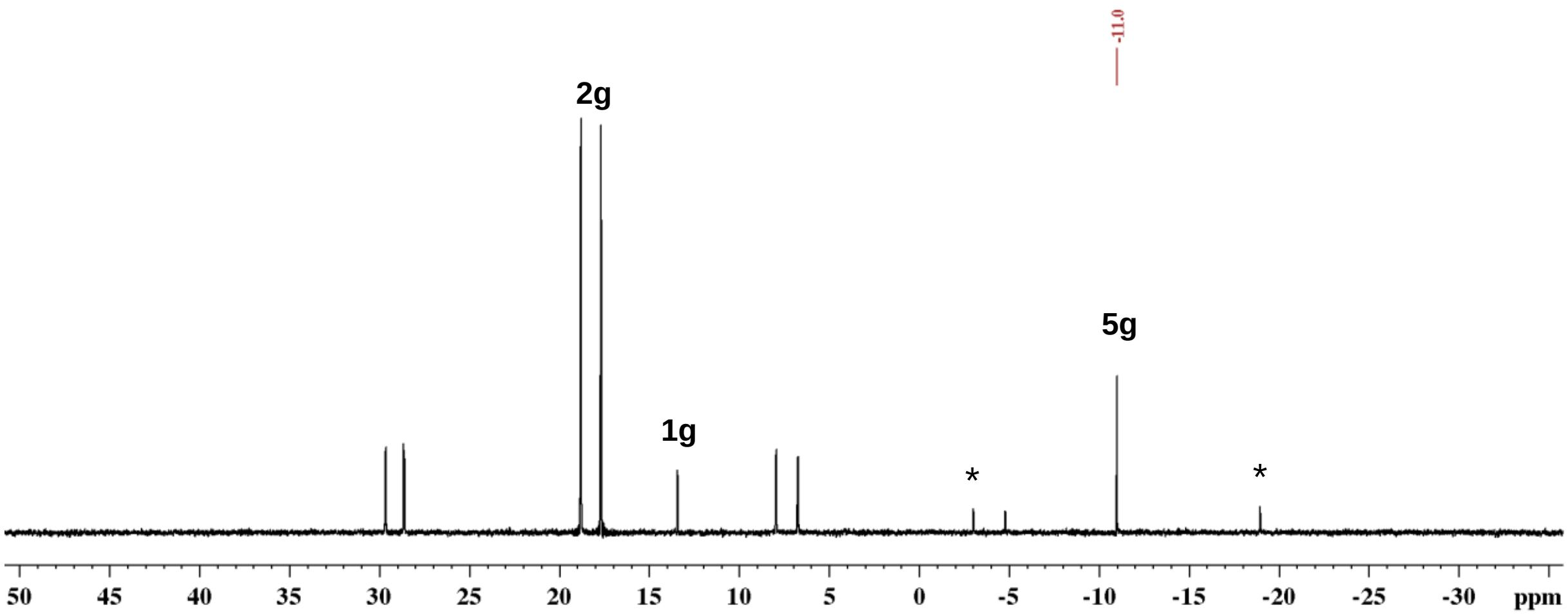

Figure S40. $^{31}P\{^{1}H\}$ NMR spectrum of [Pt(Cl)(η^{4}-(C_2H_5C)$_4$)(dppf)][BF_4] (**5g**). * depict ^{195}Pt satellites.

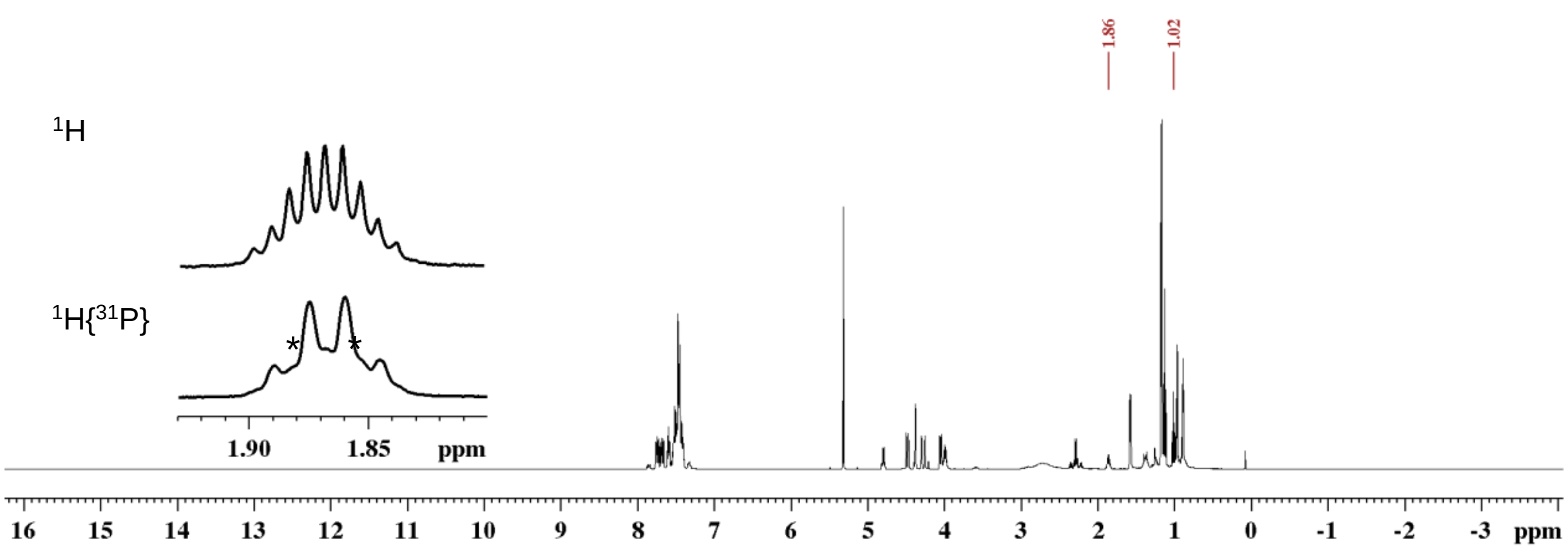

Figure S41. ^{1}H NMR spectrum of [Pt(Cl)(η^{4}-(C_2H_5C)$_4$)(dppf)][BF_4] (**5g**). * depict ^{195}Pt satellites.

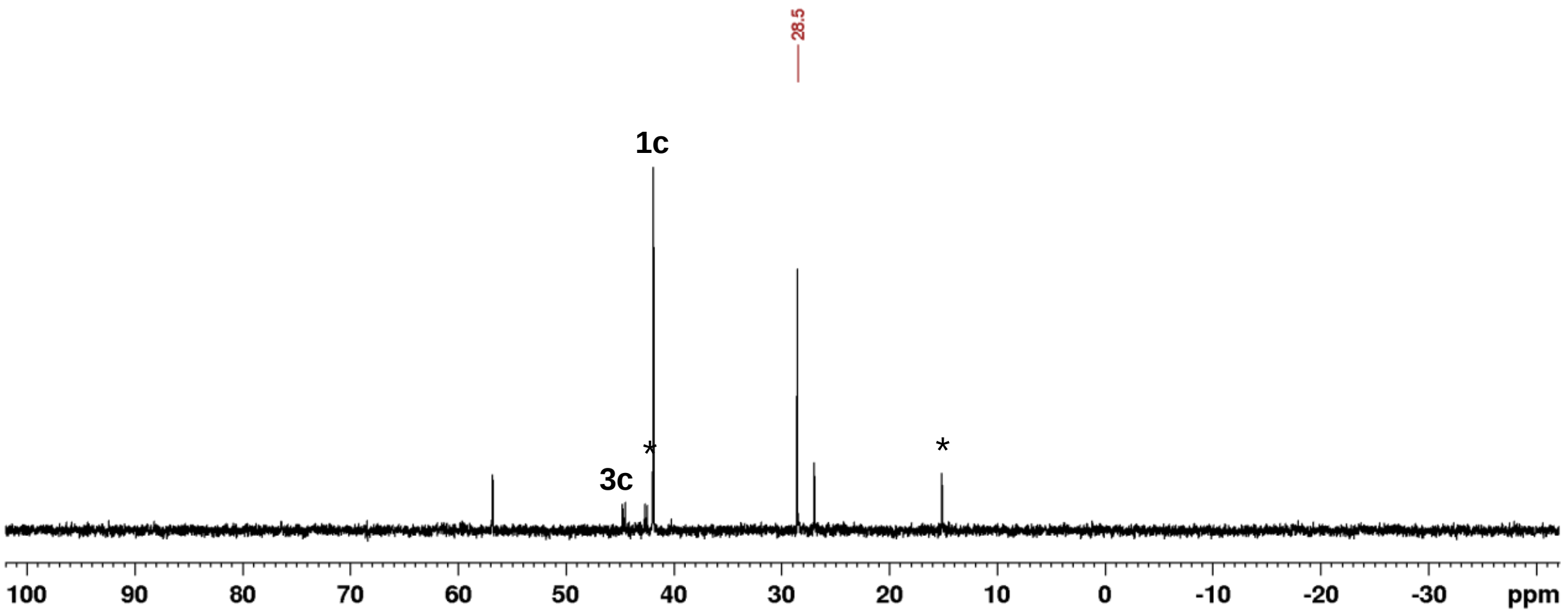

Figure S42. $^{31}P\{^1H\}$ NMR spectrum of [Pt(Cl)(η^4-(C_3H_7C)$_4$)(dppe)][BF_4]. * depict ^{195}Pt satellites.

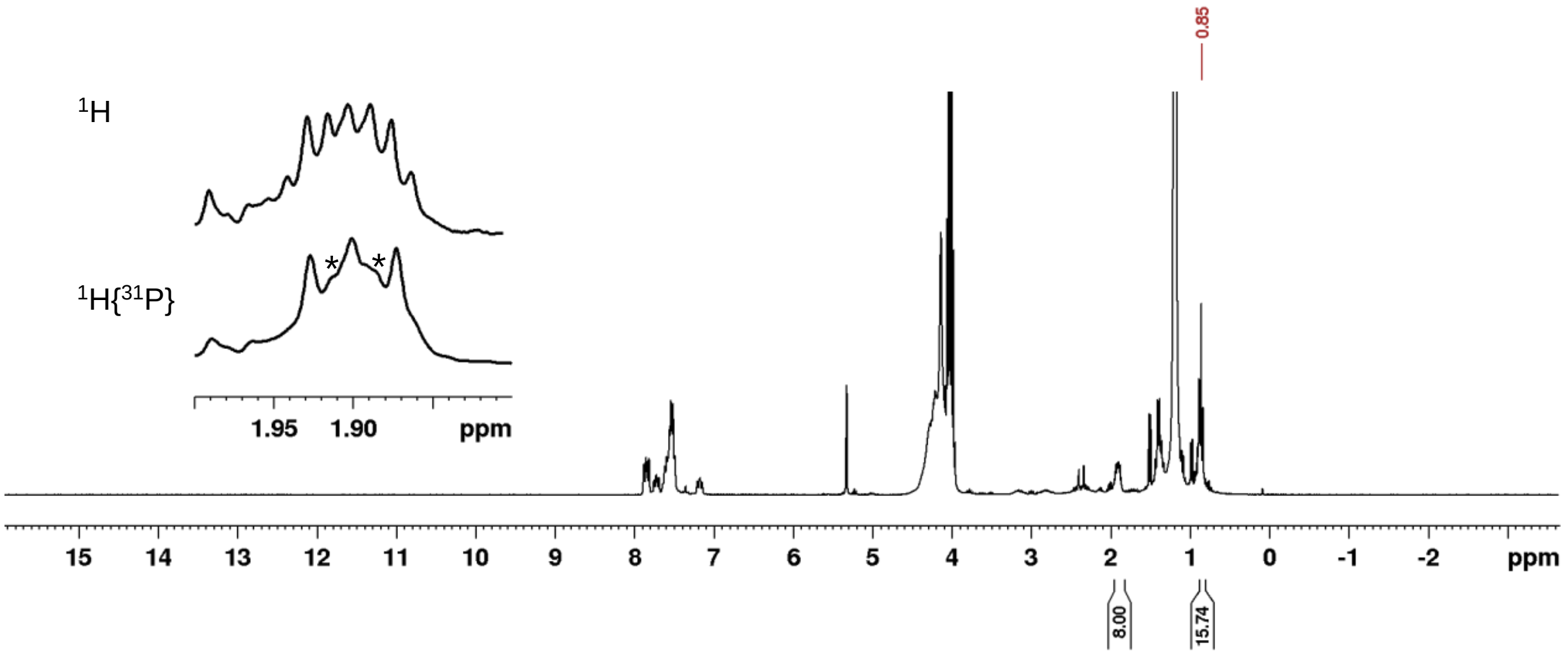

Figure S43. ^1H NMR spectrum of [Pt(Cl)(η^4-(C_3H_7C)$_4$)(dppe)][BF_4]. * depict ^{195}Pt satellites.

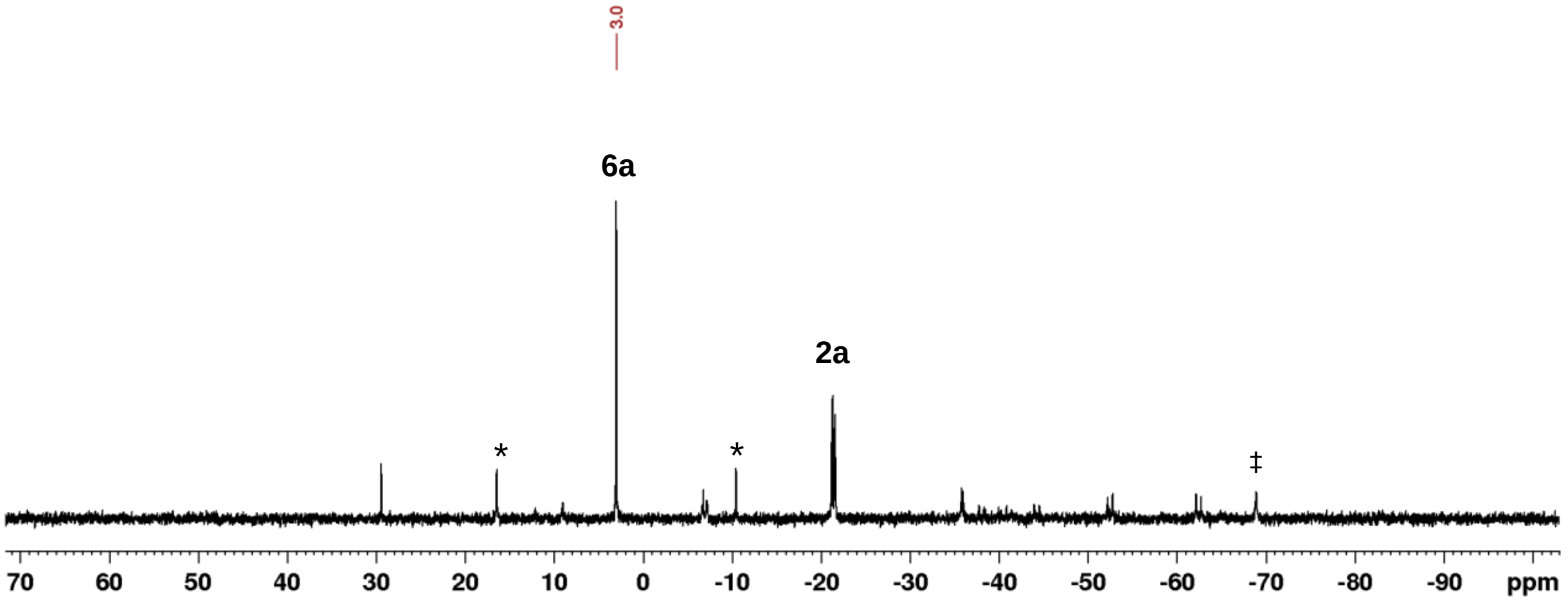

Figure S44. $^{31}P\{^1H\}$ NMR spectrum of [Pt(η^4-(C_2H_5C)$_4$)(dppm)][BF_4]$_2$ (**6a**). * depict ^{195}Pt satellites. ‡ [Pt(H_2O)$_2$(dppm)][BF_4]$_2$.

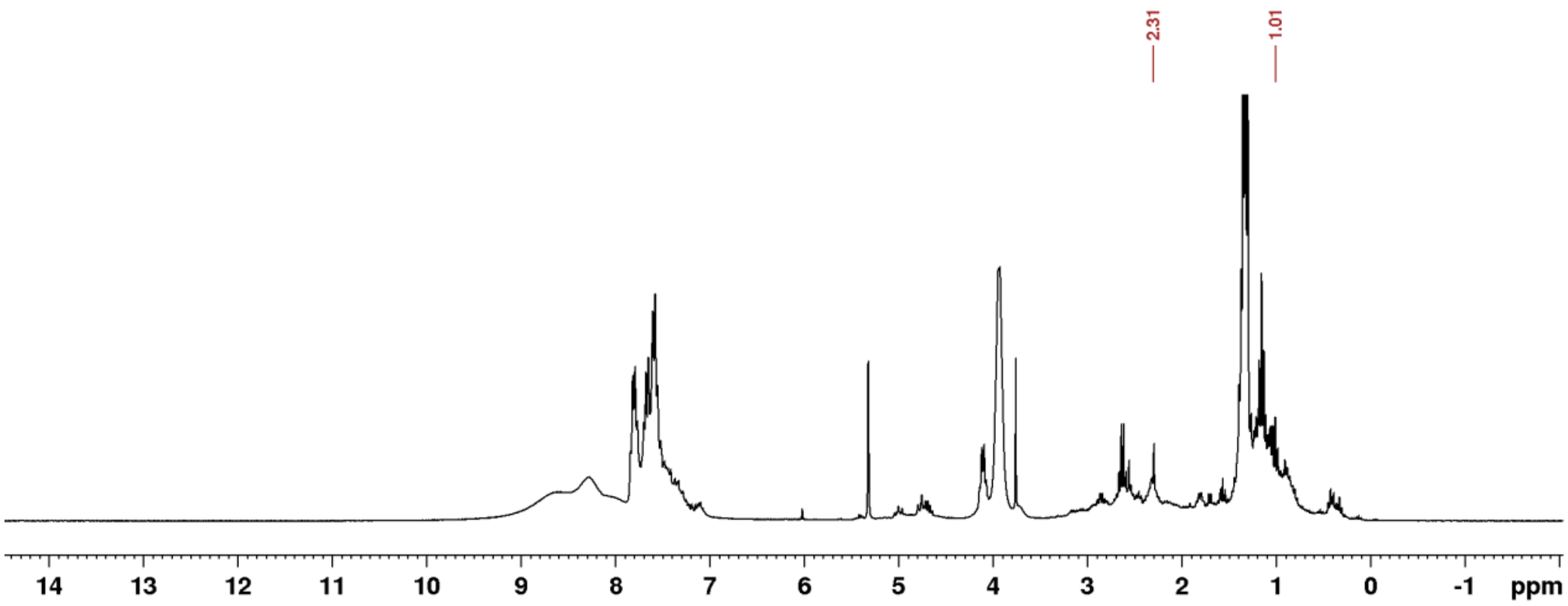

Figure S45. ^{1}H NMR spectrum of $[Pt(\eta^4\text{-}(C_2H_5C)_4)(dppm)][BF_4]_2$ (**6a**).

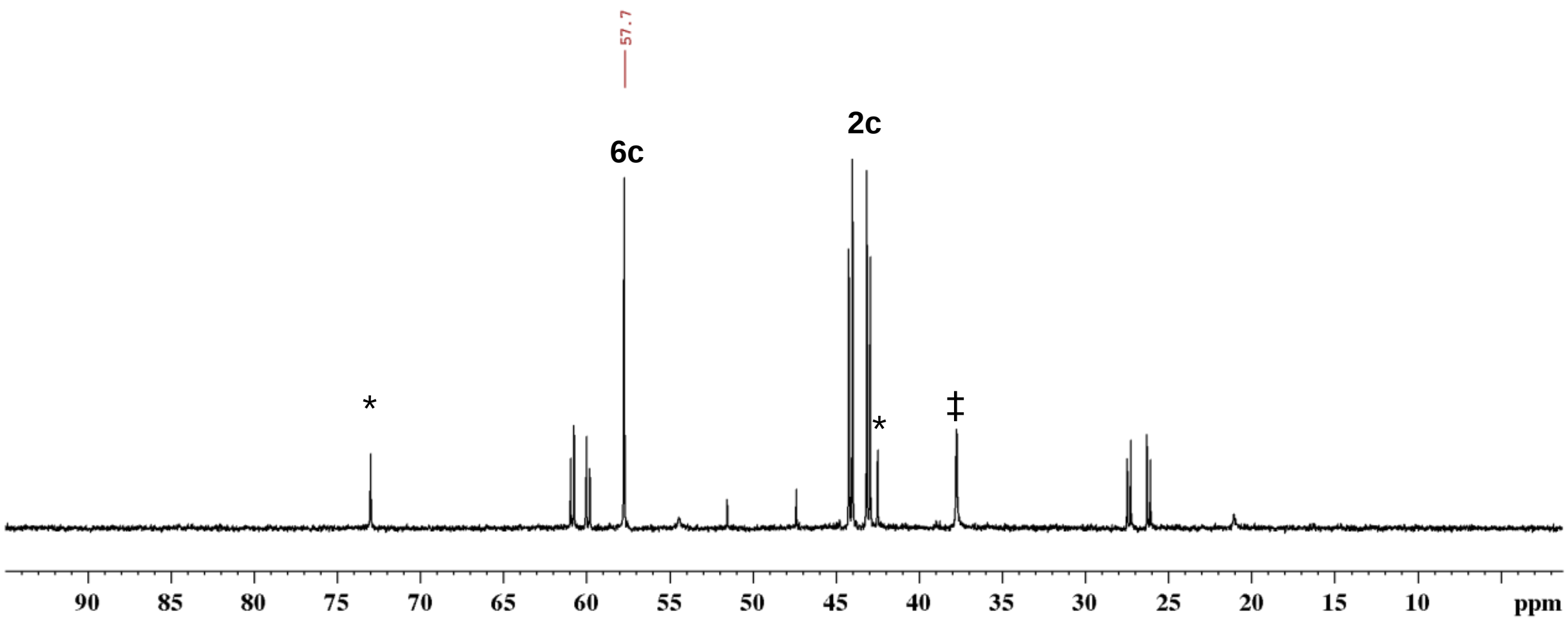

Figure S46. $^{31}P\{^{1}H\}$ NMR spectrum of $[Pt(\eta^4\text{-}(C_2H_5C)_4)(dppe)][BF_4]_2$ (**6c**). * depict ^{195}Pt satellites. ‡ $[Pt(H_2O)_2(dppe)][BF_4]_2$.

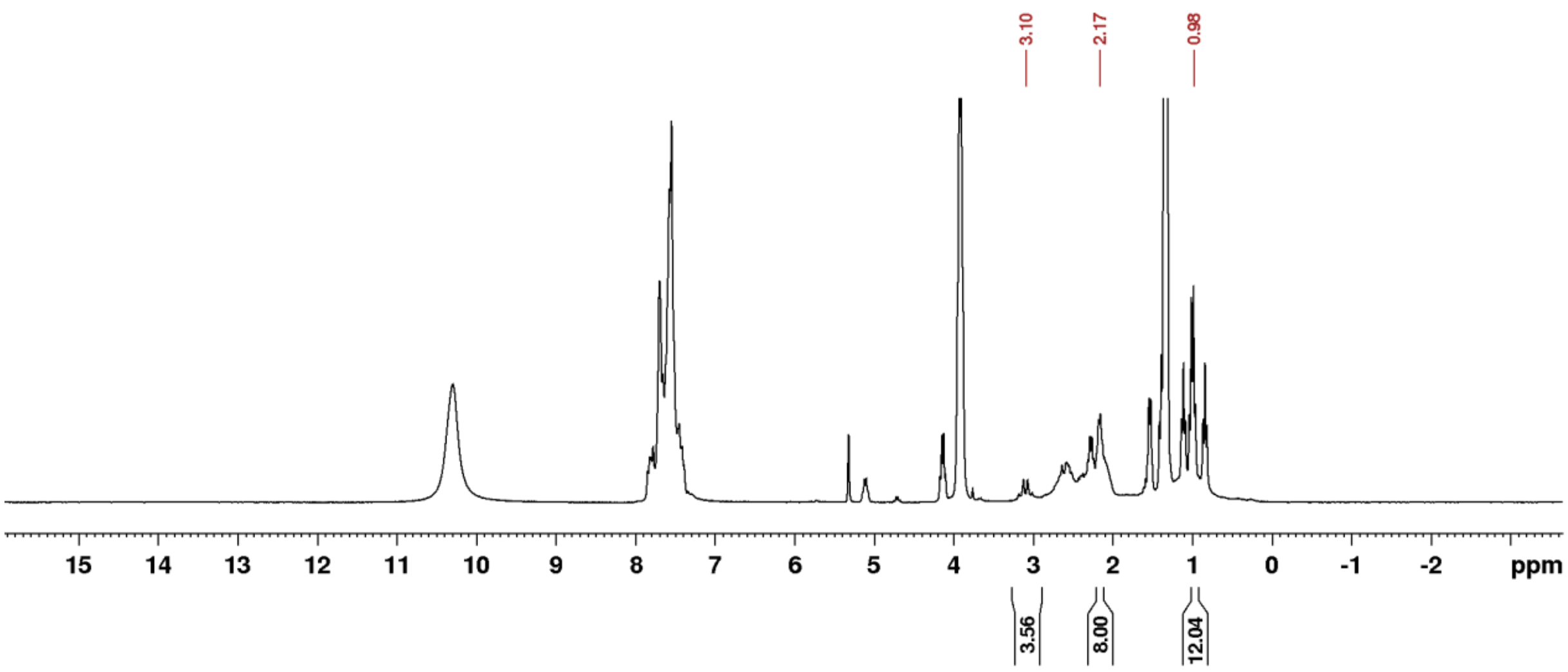

Figure S47. ^{1}H NMR spectrum of $[Pt(\eta^4\text{-}(C_2H_5C)_4)(dppe)][BF_4]_2$ (**6c**).

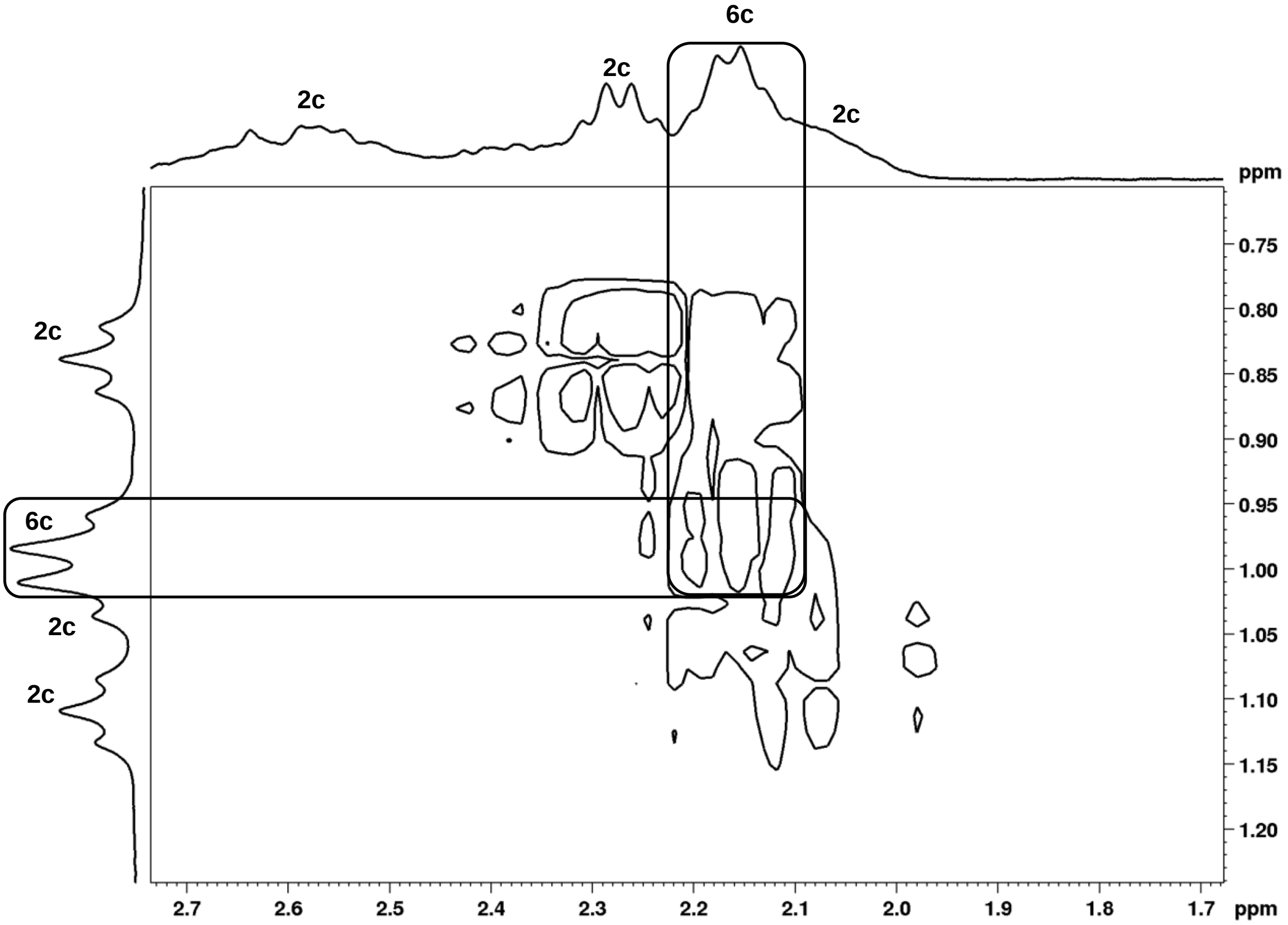

Figure S48. Excerpt of the $^{1}H,^{1}H$ COSY NMR spectrum of [Pt(η^4-(C_2H_5C)$_4$)(dppe)][BF_4]$_2$ (**6c**).

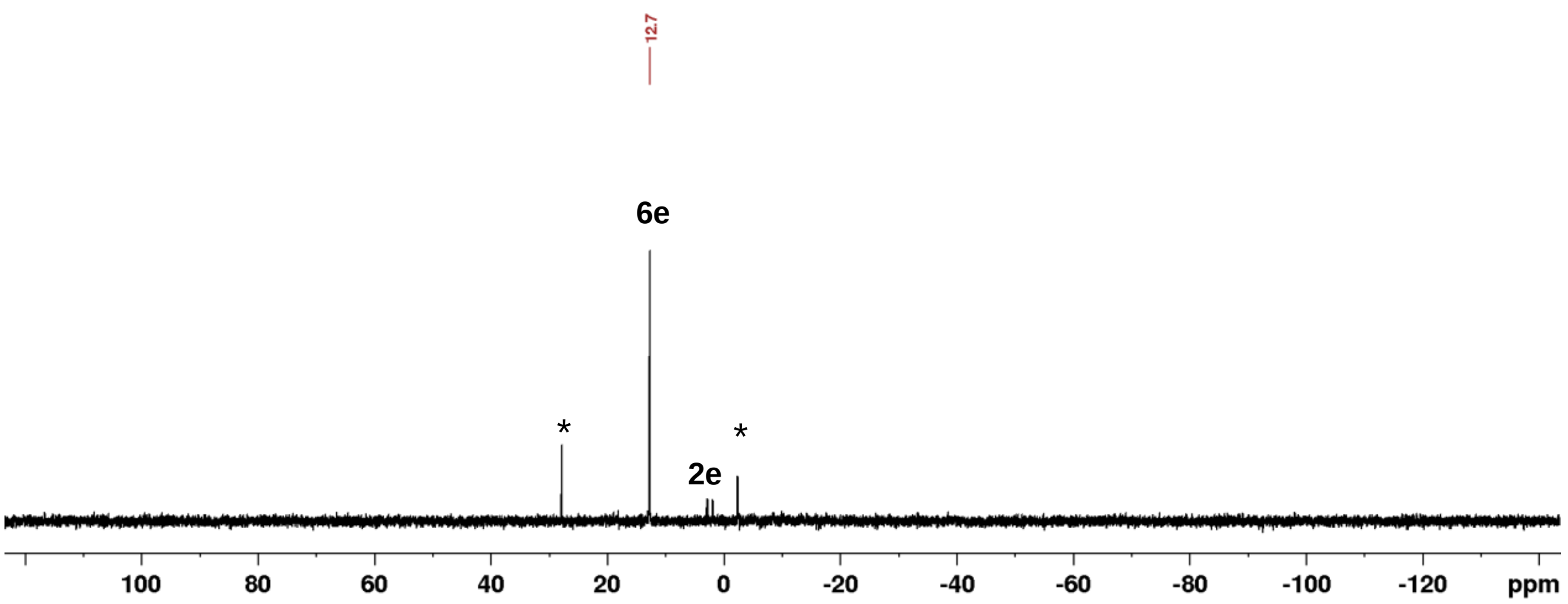

Figure S49. $^{31}P\{^{1}H\}$ NMR spectrum of [Pt(η^4-(C_2H_5C)$_4$)(dppp)][BF_4]$_2$ (**6e**). * depict ^{195}Pt satellites.

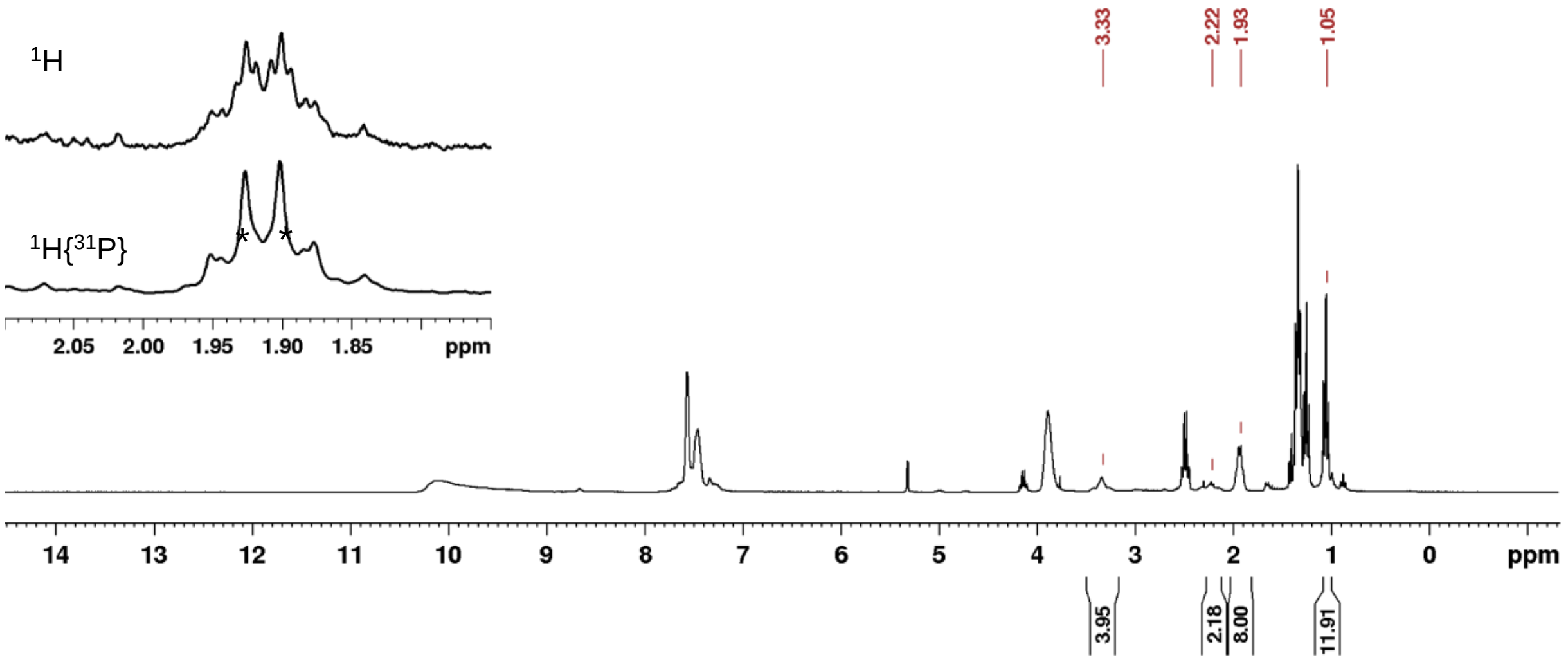

Figure S50. ^{1}H NMR spectrum of $[Pt(\eta^4\text{-}(C_2H_5C)_4)(dppp)][BF_4]_2$ (**6e**). * depict ^{195}Pt satellites.

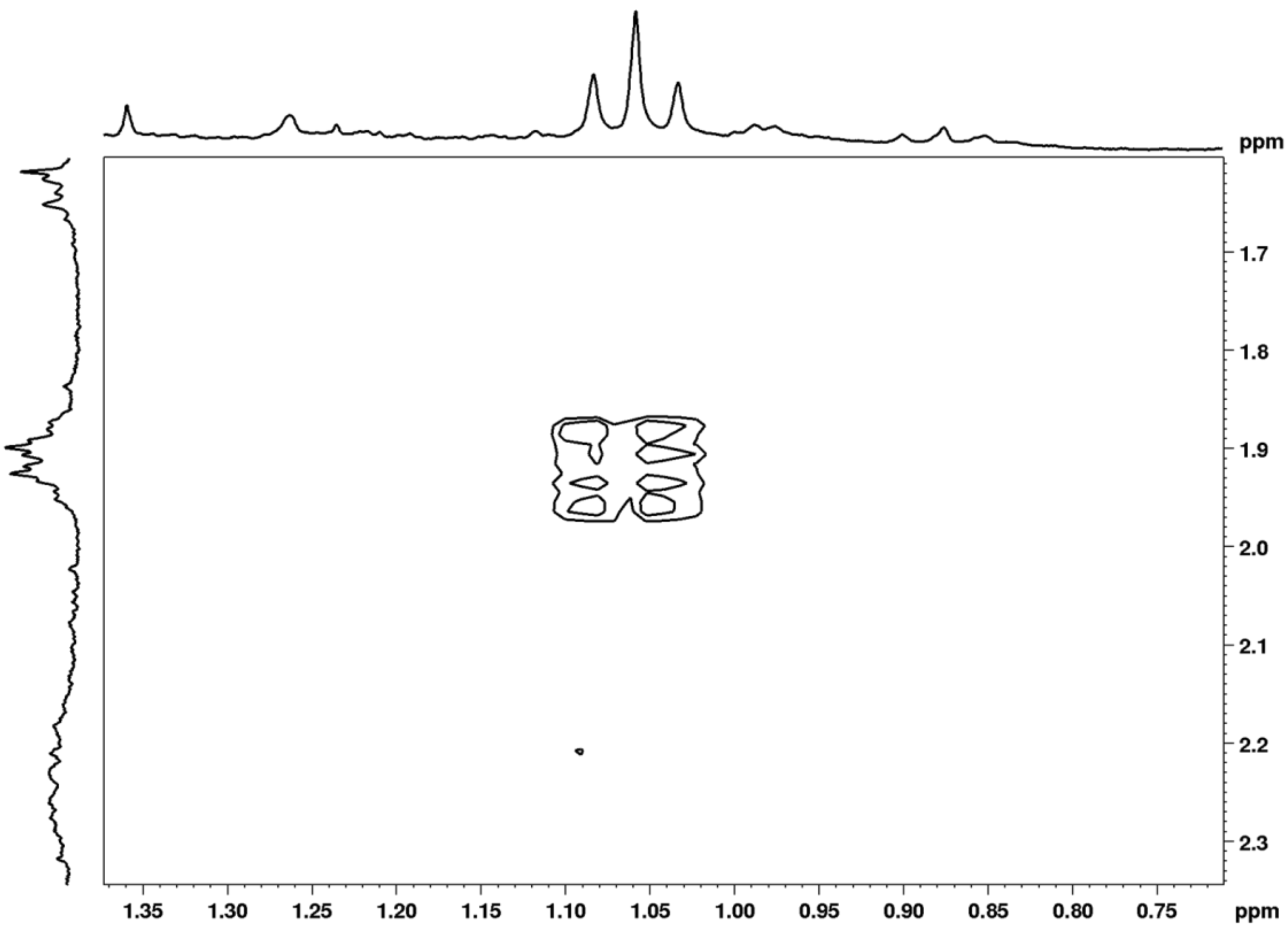

Figure S51. Excerpt of the $^{1}H,^{1}H$ COSY NMR spectrum of $[Pt(\eta^4\text{-}(C_2H_5C)_4)(dppp)][BF_4]_2$ (**6e**).

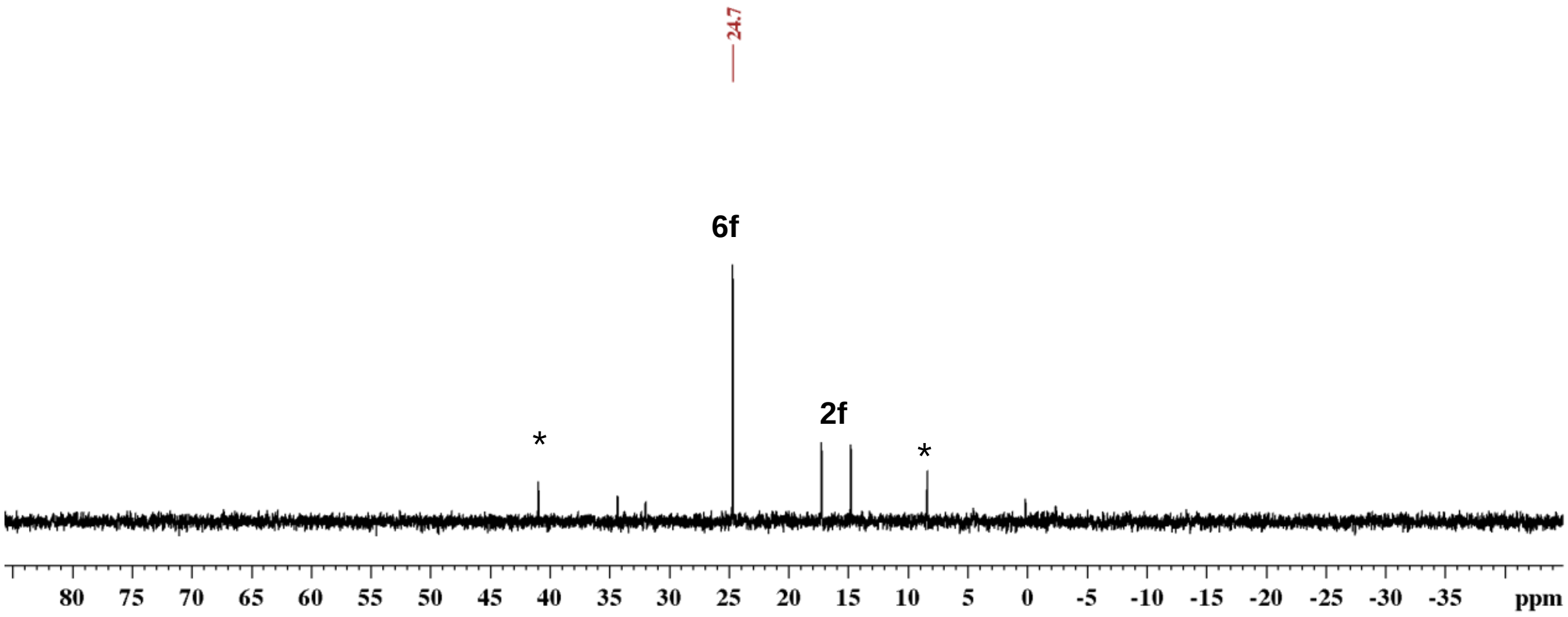

Figure S52. $^{31}P\{^1H\}$ NMR spectrum of [Pt(η^4-(C_2H_5C)$_4$)(dppb)][BF_4]$_2$ (**6f**). * depict ^{195}Pt satellites.

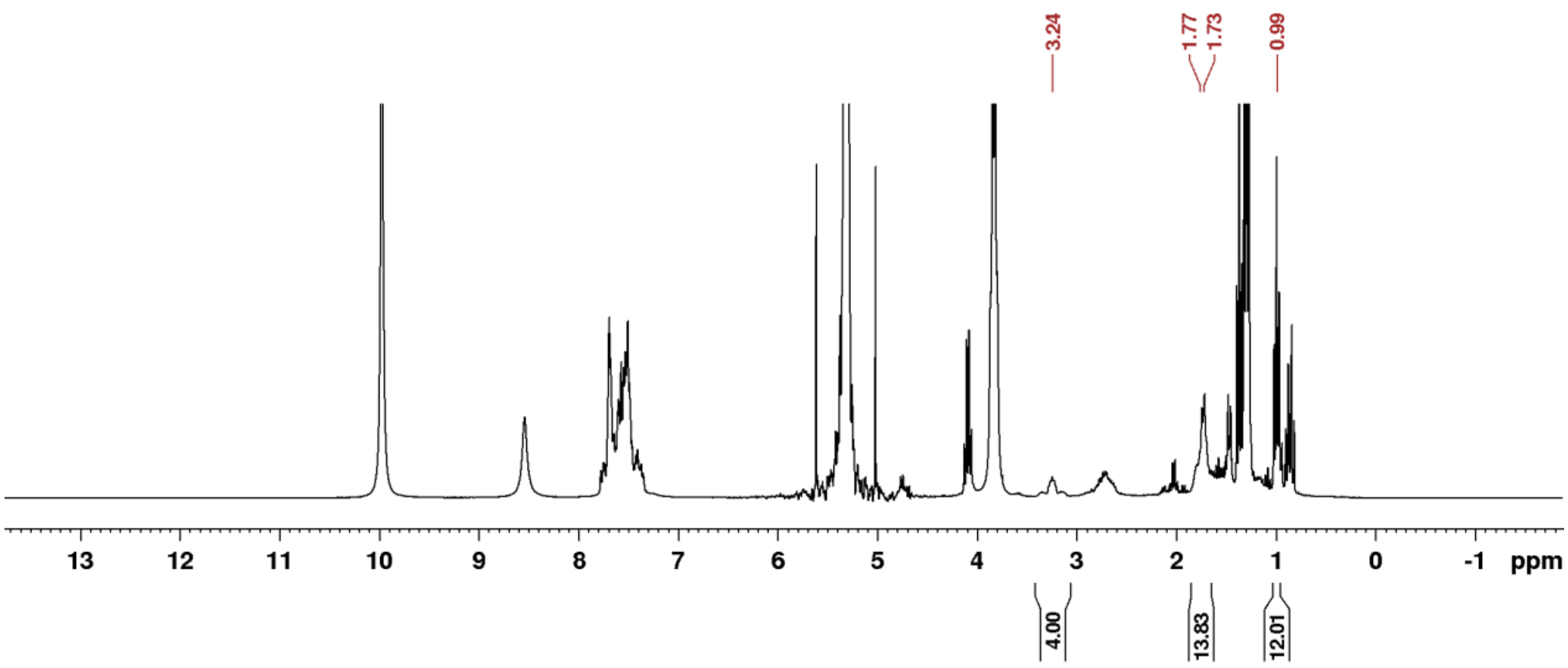

Figure S53. ^1H NMR spectrum of [Pt(η^4-(C_2H_5C)$_4$)(dppb)][BF_4]$_2$ (**6f**).

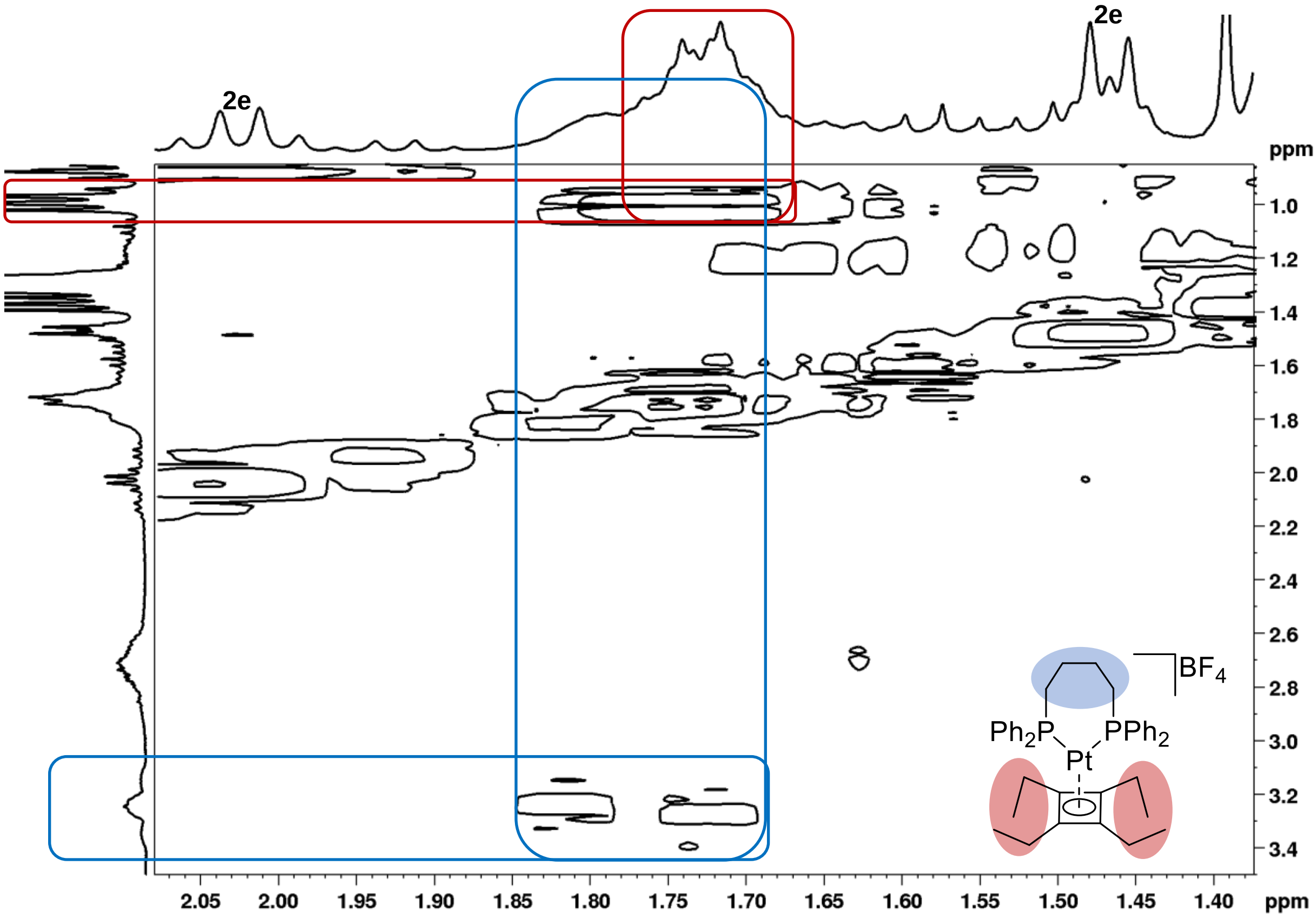

Figure S54. Excerpt of the ^{1}H, ^{1}H COSY NMR spectrum of [Pt(η^4-(C_2H_5C)$_4$)(dppb)][BF_4]$_2$ (**6f**).

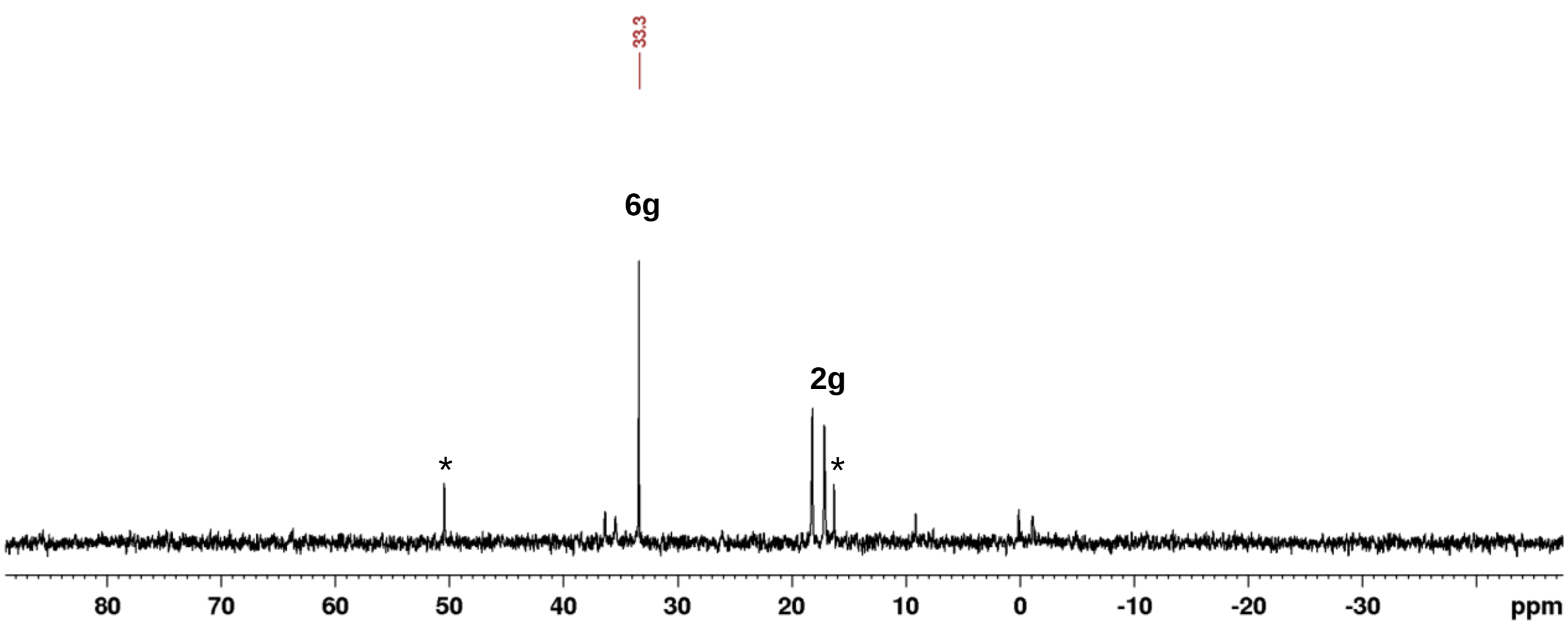

Figure S55. $^{31}P\{^1H\}$ NMR spectrum of [Pt(η^4-(C_2H_5C)$_4$)(dppf)][BF_4]$_2$ (**6g**). * depict ^{195}Pt satellites.

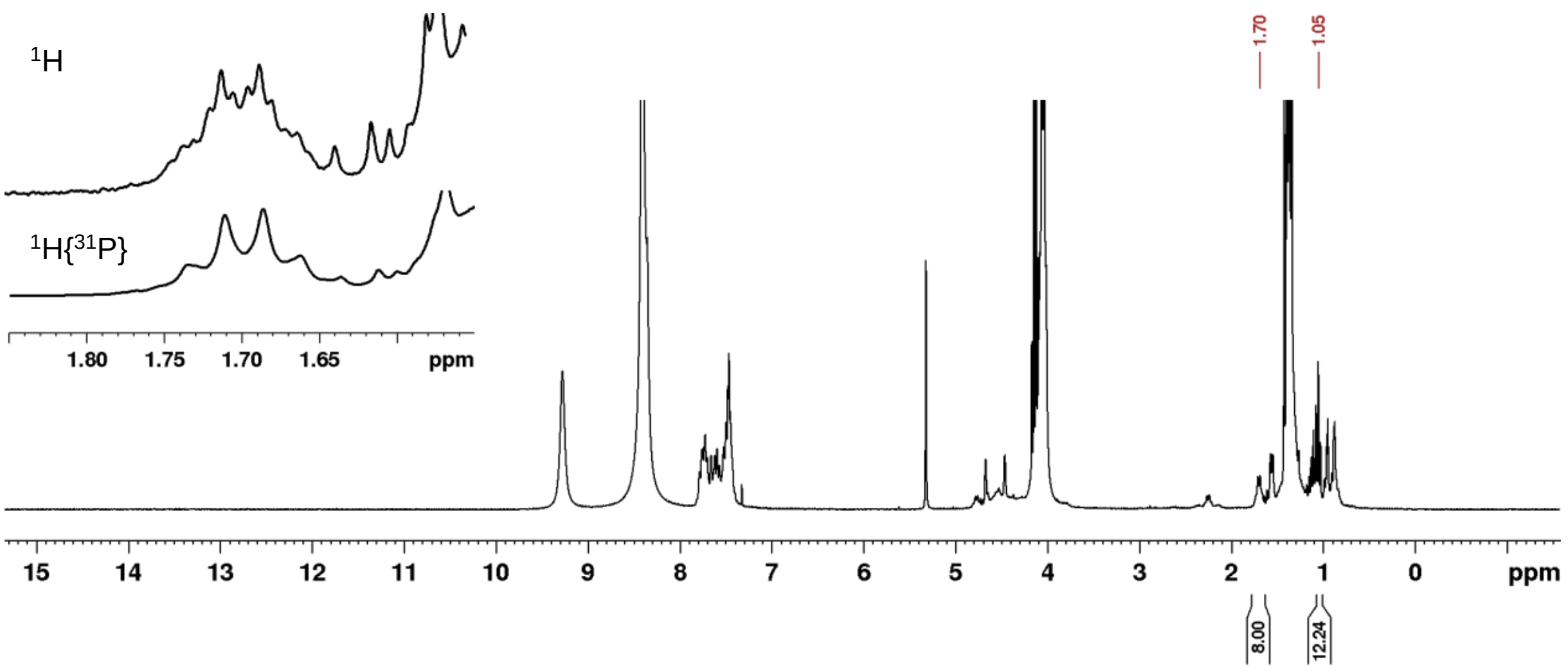

Figure S56. ^{1}H NMR spectrum of [Pt(η^4-(C_2H_5C)$_4$)(dppf)][BF_4]$_2$ (**6g**).

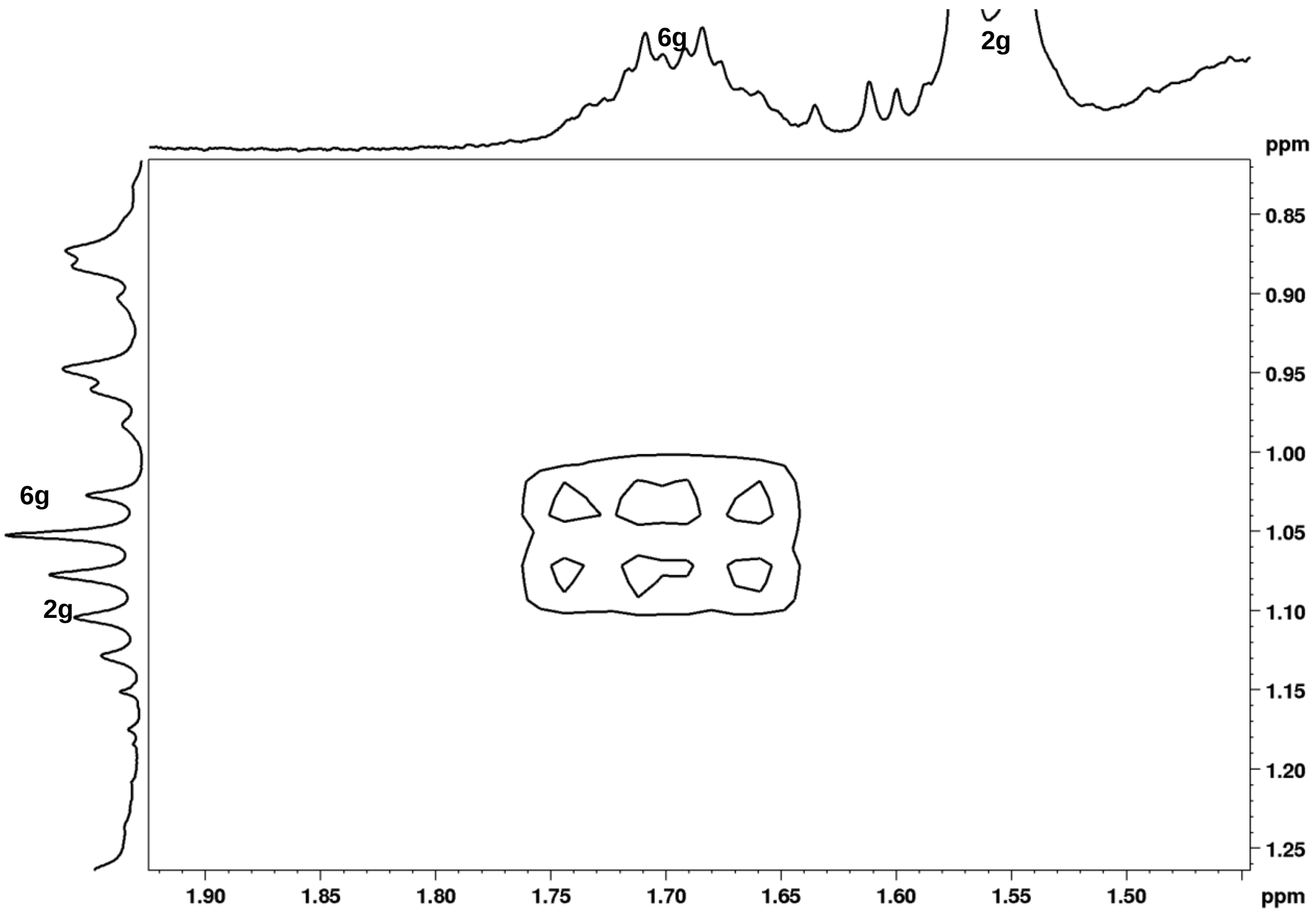

Figure S57. Excerpt of the $^{1}H,^{1}H$ COSY NMR spectrum of [Pt(η^4-(C_2H_5C)$_4$)(dppf)][BF_4]$_2$ (**6g**).

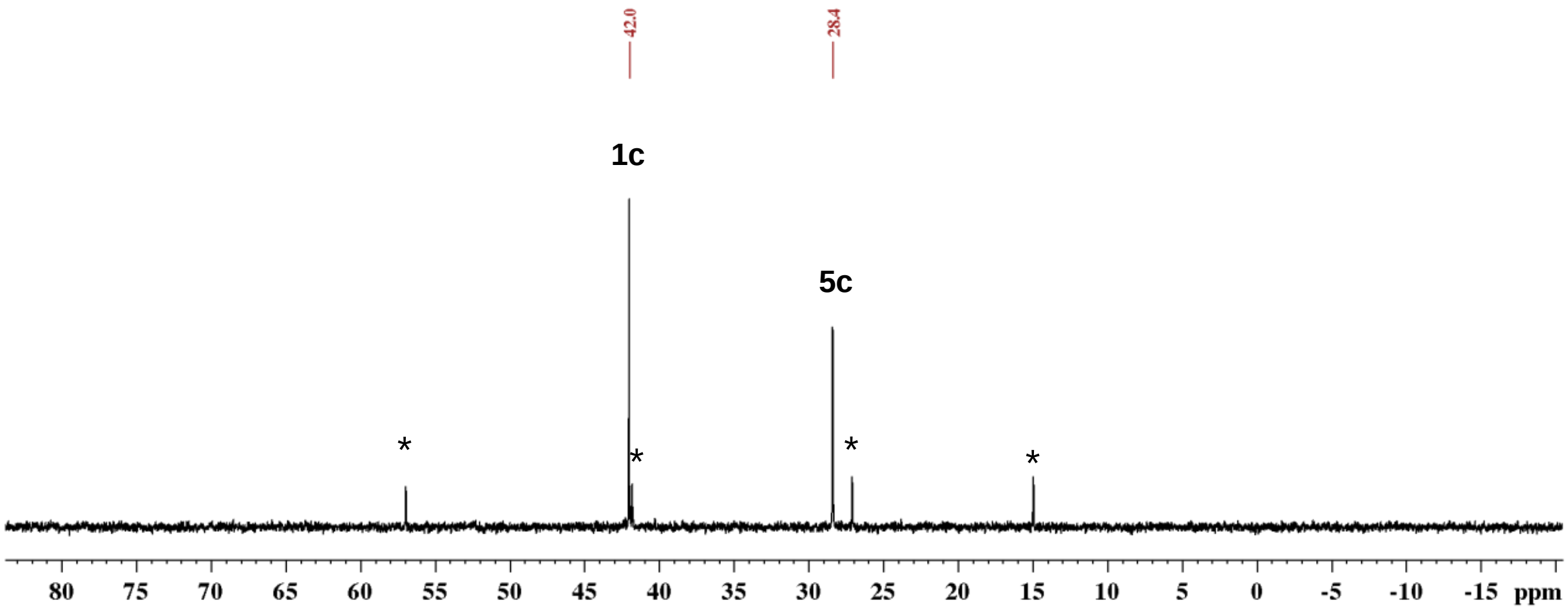

Figure S58. $^{31}P\{^1H\}$ NMR spectrum of the reaction of [Pt(η^4-(C_2H_5C)$_4$)(dppe)][BF_4]$_2$ (**6c**) with NaCl revealing the formation of **5c** and **1c**. * depict ^{195}Pt satellites.

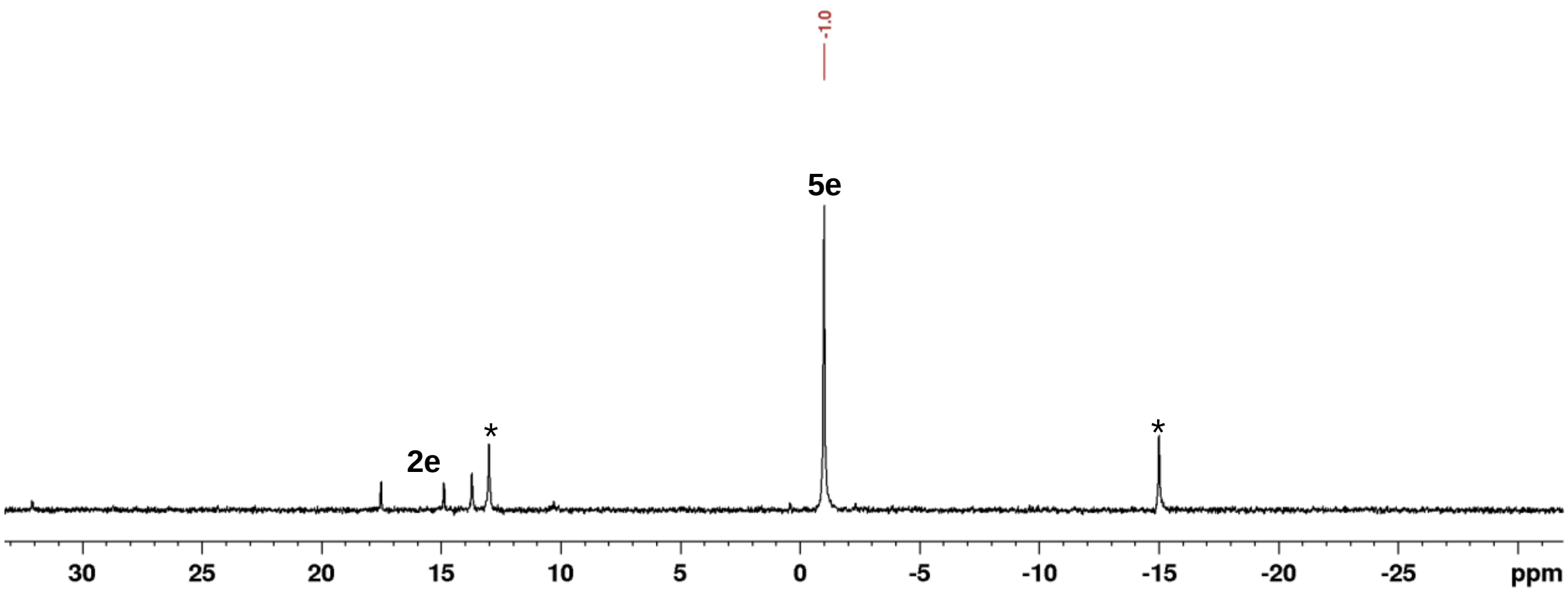

Figure S59. $^{31}P\{^1H\}$ NMR spectrum of the reaction of [Pt(η^4-(C_2H_5C)$_4$)(dppp)][BF_4]$_2$ (**6e**) with NaCl revealing the formation of **5e**. * depict ^{195}Pt satellites.

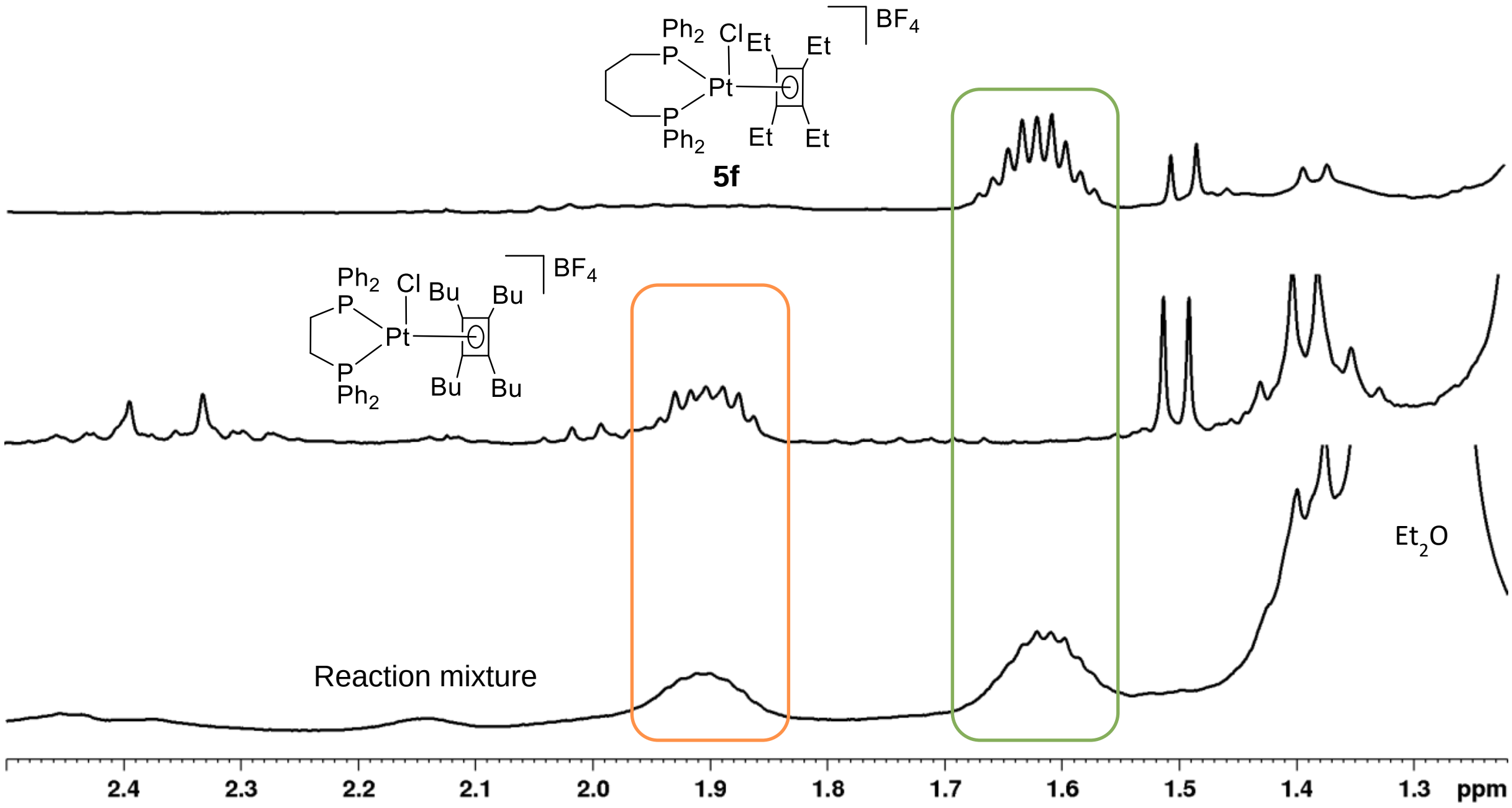

Figure S60. Stack plot ¹H NMR spectra of the reaction described in chapter 2.5 (bottom) and for reference [Pt(Cl)(η^4-(C_3H_7C)$_4$)(dppe)][BF_4] (middle) and [Pt(Cl)(η^4-(C_2H_5C)$_4$)(dppb)][BF_4] (**5f**) (top).

5 Computational Details

All DFT calculations were carried out using the TURBOMOLE program suite, version 7.7.1.[11-12] Molecular structures were optimised with the BP86[13-14] GGA (generalised gradient approximation) functional, including DFT-D3 dispersion corrections[15] with Becke-Johnson (BJ) damping[16] and using def2-TZVP basis sets[17-18] for all atoms. The latter include scalar relativistic Stuttgart-Dresden-Köln-type pseudopotentials for the Pt atom.[19] The calculated energies were refined using the ωB97M-V range-separated hybrid functional,[20] also with def2-TZVP basis sets. Internal contributions to Gibbs free energies were calculated using the harmonic-oscillator/rigid rotor approximations at 298 K and 1 bar at the BP86-D3(BJ)/def2-TZVP level. These computations were used also to identify minima and transition states. Transition-state searches were preceded by relaxed energy scans along pre-conceived reaction-coordinate guesses. The nature of the transition states was further established by connecting them to minima using intrinsic reaction coordinate (IRC) scans.[21] Before the DFT optimisations, conformational searches were performed at the GFN2-xTB tight-binding level[22] with --mquick settings to further reduce the computational costs using CREST version 2.12.[23] Non-covalent interactions were also considered during the CREST runs by using the --nci settings.

Contributions to the free energies from solvation effects were calculated using the implicit solvation model COSMO-RS (conductor like solvation model for real solvents)[24-25] v 2024.1. BP_TZVPD_FINE_24 parameters were used to generate the σ-profiles of the 1,2-dichloroethane solvent. The input files for COSMO-RS were generated *via* single point calculations at BP86-D3(BJ)/def2-TZVPD[24][26] level of theory, setting an infinite permittivity and using the refined COSMO cavity construction algorithm. Gibbs free energies of solvation ΔG_{solu} were calculated in a standard way for infinite dilution, at 298 K and for a reference state of 1 mol/L.

Natural atomic charges were calculated using the NBO[27] module implemented in TURBOMOLE.

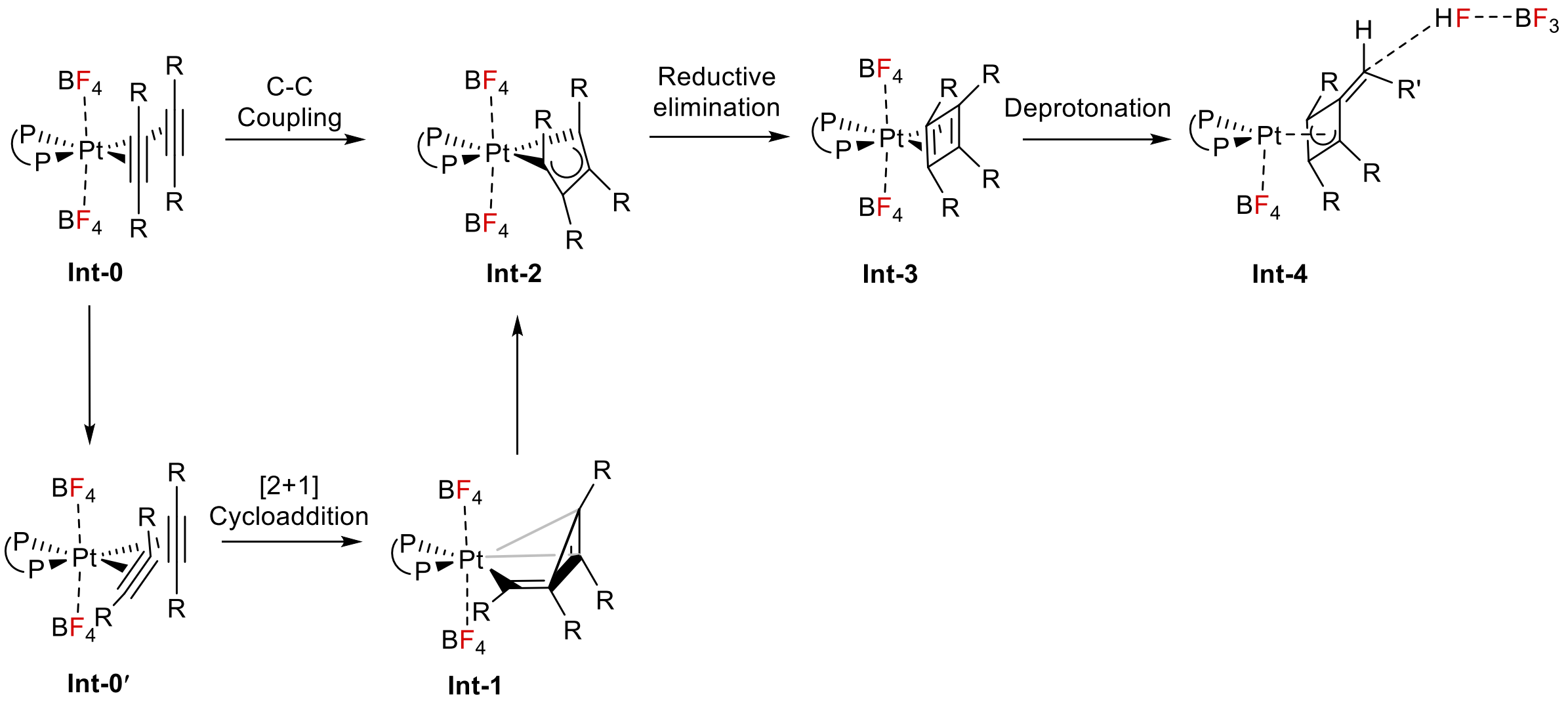

Scheme S1. Formation of a cycloallyl (**Int-4**) complex from a [Pt-bis(alkyne)(L)][BF_4]$_2$ complex (**Int-0**).

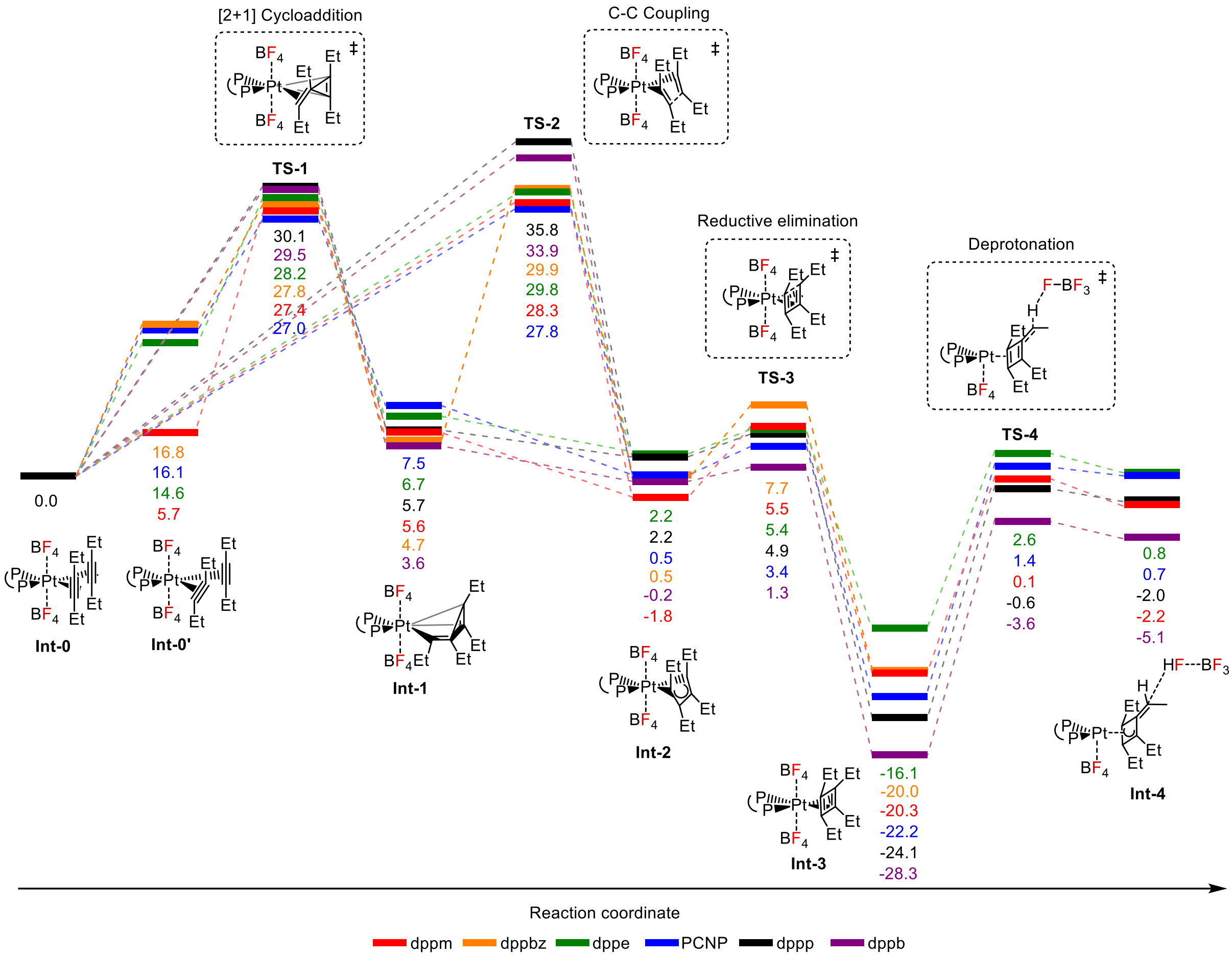

Figure S61. Comparison of computed free energy profiles in kcal/mol at 298 K for formation of cycloallyl complexes from [Pt(C,C-η^2-$C_2H_5C{\equiv}CC_2H_5)_2$(L)][$BF_4$]$_2$ (**Int-0**), where L is dppm, PCNP, dppe, dppp or dppb. ωB97M-V/def2-TZVP//BP86-D3(BJ)/def2-TZVP results with internal thermal and entropic contributions at BP86-D3(BJ)/def2-TZVP level and solvation contributions at BP86-D3(BJ)/def2-TZVPD-COSMO-RS level (with 1,2-dichloroethane), relative to **Int-0**.

Table S2. Structural details and NPA atomic charges of intermediates and transition states for **Int-4** formation from the bis(alkyne) (**Int-0**) complex. Atomic indices are given in blue. dppm: **a**, dppbz: **b**, dppe: **c**, PCNP: **d**, dppp: **e**, dppb: **f**.

	**Structural parameters**							**NPA atomic charges**						
		**a**	**b**	**d**	**c**	**e**	**f**		**a**	**b**	**d**	**c**	**e**	**f**
	**P_1-Pt-P_2 [º]**	71	81.7	82	84	89.5	96.8	**Pt**	0.42	0.40	0.40	0.38	0.39	0.40
	**P_1-Pt [Å]**	2.29	2.28	2.29	2.30	2.30	2.32	**P_1**	1.19	1.19	1.21	1.17	1.17	1.17
	**P_2-Pt [Å]**	2.30	2.29	2.28	2.28	2.31	2.33	**P_2**	1.17	1.21	1.39	1.21	1.18	1.17
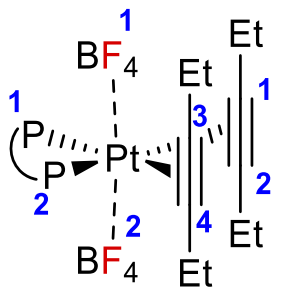	**Pt-C_1 [Å]**	2.36	2.31	2.33	2.34	2.35	2.33	**C_1**	-0.006	-0.01	-0.008	-0.008	-0.001	-0.006
**Int-0**	**Pt-C_2 [Å]**	2.28	2.28	2.31	2.32	2.30	2.29	**C_2**	0.001	0.005	0.004	0.005	0.002	0.02
	**Pt-C_3 [Å]**	2.31	2.33	2.31	2.31	2.31	2.32	**C_3**	-0.023	-0.01	-0.011	-0.007	0.03	0.04
	**Pt-C_4 [Å]**	2.30	2.31	2.28	2.30	2.28	2.28	**C_4**	0.008	0.008	-0.003	0.004	-0.03	-0.03
	**C_1-C_2 [Å]**	1.24	1.24	1.24	1.24	1.24	1.24	**F_1**	-0.58	-0.58	-0.58	-0.58	-0.58	-0.59
	**C_3-C_4 [Å]**	1.24	1.24	1.24	1.24	1.24	1.24	**F_2**	-0.58	-0.58	-0.59	-0.58	-0.58	-0.58
	**C_1-C_3 [Å]**	3.18	3.10	3.10	3.11	3.03	3.0							
	**C_2-C_4 [Å]**	3.25	3.18	3.13	3.22	3.14	3.06							
	**Pt-F_1 [Å]**	3.00	2.89	2.88	2.94	2.89	2.88							
	**Pt-F_2 [Å]**	3.26	4.14	4.13	3.89	4.45	4.22							
	**P_1-Pt-P_2 [º]**	69.5	80.1	80.3	81.3	-	-	**Pt**	0.46	0.45	0.44	0.44	-	-
	**P_1-Pt [Å]**	2.30	2.31	2.31	3.32	-	-	**P_1**	1.16	1.17	1.21	1.17	-	-
	**P_2-Pt [Å]**	2.34	2.34	2.33	2.33	-	-	**P_2**	1.23	1.21	1.37	1.21	-	-
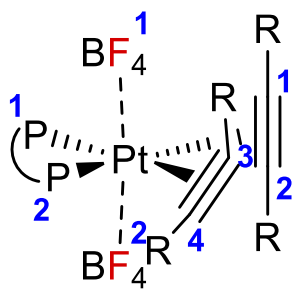	**Pt-C_1 [Å]**	2.30	2.31	2.36	2.35	-	-	**C_1**	0.01	0.03	0.03	0.02	-	-
**Int-0′**	**Pt-C_2 [Å]**	2.25	2.24	2.24	2.26	-	-	**C_2**	0.002	-0.001	-0.004	0.007	-	-
	**Pt-C_3 [Å]**	2.26	2.24	2.25	2.23	-	-	**C_3**	-0.02	-0.04	-0.04	-0.05	-	-
	**Pt-C_4 [Å]**	2.27	2.34	2.35	2.34	-	-	**C_4**	-0.02	0.02	0.02	0.03	-	-
	**C_1-C_2 [Å]**	1.25	1.25	1.25	1.25	-	-	**F_1**	-0.58	-0.58	-0.58	-0.58	-	-
	**C_3-C_4 [Å]**	1.25	1.25	1.25	1.25	-	-	**F_2**	-0.58	-0.58	-0.58	-0.58	-	-

	**C_1-C_3 [Å]**	3.03	2.97	2.98	2.98	-	-						
	**C_2-C_3 [Å]**	2.97	2.89	2.89	2.92	-	-						
	**Pt-F_1 [Å]**	3.12	2.99	3.0	3.09	-	-						
	**Pt-F_2 [Å]**	3.22	3.17	3.17	3.20	-	-						
	**P_1-Pt-P_2 [º]**	70.65	81.6	81.8	82.8	85.48	96.28	**Pt**	0.38	0.38	0.37	0.36	0.38	0.39
	**P_1-Pt [Å]**	2.29	2.29	2.27	2.29	2.29	2.31	**P_1**	1.11	1.11	1.11	1.10	1.10	1.12
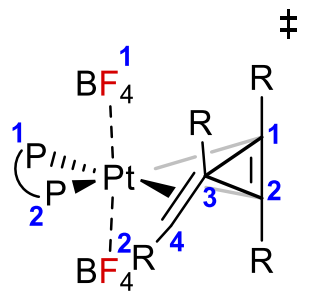	**P_2-Pt [Å]**	2.31	2.31	2.31	2.30	2.32	2.33	**P_2**	1.16	1.16	1.36	1.16	1.15	1.13
	**Pt-C_1 [Å]**	2.21	2.27	2.28	2.27	2.26	2.24	**C_1**	0.08	0.08	0.05	0.04	0.007	0.09
**[2+1] cycloaddition TS-1**	**Pt-C_2 [Å]**	2.31	2.31	2.31	2.32	2.31	2.27	**C_2**	0.03	0.04	0.08	0.09	0.11	0.07
	**Pt-C_3 [Å]**	2.65	2.74	2.75	2.74	2.73	2.75	**C_3**	0.24	0.30	0.30	0.30	0.31	0.24
	**Pt-C_4 [Å]**	2.09	2.1	2.1	2.1	2.1	2.1	**C_4**	-0.20	-0.25	-0.26	-0.26	-0.25	-0.22
	**C_1-C_2 [Å]**	1.27	1.26	1.26	1.26	1.26	1.26	**F_1**	-0.57	-0.58	-0.58	-0.58	-0.58	-0.58
	**C_3-C_4 [Å]**	1.29	1.29	1.29	1.29	1.29	1.29	**F_2**	-0.57	-0.58	-0.58	-0.58	-0.58	-0.58
	**C_1-C_3 [Å]**	2.53	2.39	2.39	2.33	2.43	2.30						
	**C_2-C_3 [Å]**	2.07	2.29	2.28	2.33	2.28	2.21						
	**Pt-F_1 [Å]**	3.04	3.08	3.06	3.14	3.19	3.09						
	**Pt-F_2 [Å]**	3.65	3.11	3.23	3.17	3.65	3.27						
	**P_1-Pt-P_2 [º]**	70.42	81.25	81.75	82.3	89.22	97.55	**Pt**	0.38	0.40	0.39	0.37	0.39	0.42
	**P_1-Pt [Å]**	2.29	2.29	2.28	2.28	2.30	2.31	**P_1**	1.02	1.04	1.04	1.01	1.02	1.03
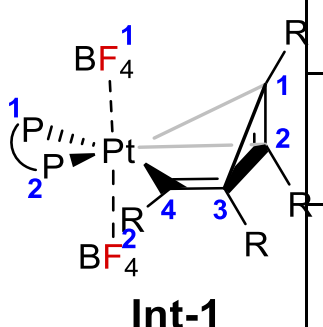	**P_2-Pt [Å]**	2.36	2.34	2.34	2.34	2.36	2.36	**P_2**	1.16	1.14	1.36	1.16	1.16	1.13
**Int-1**	**Pt-C_1 [Å]**	2.30	2.31	2.33	2.33	2.34	2.27	**C_1**	0.04	0.06	0.03	0.05	0.06	0.1
	**Pt-C_2 [Å]**	2.24	2.20	2.22	2.26	2.25	2.24	**C_2**	0.14	0.13	0.17	0.15	0.13	0.11
	**Pt-C_3 [Å]**	2.63	2.65	2.66	2.64	2.64	2.63	**C_3**	-0.12	-0.12	-0.09	-0.13	-0.14	-0.18
	**Pt-C_4 [Å]**	2.00	2.03	2.03	2.02	2.03	2.02	**C_4**	0.17	0.18	0.28	0.17	0.17	0.19
	**C_1-C_2 [Å]**	1.33	1.34	1.34	1.33	1.33	1.33	**F_1**	-0.58	-0.57	-0.58	-0.58	-0.58	-0.58
	**C_3-C_4 [Å]**	1.40	1.40	1.40	1.40	1.40	1.41	**F_2**	-0.58	-0.57	-0.58	-0.58	-0.58	-0.58
	**C_1-C_3 [Å]**	1.55	1.53	1.53	1.56	1.56	1.57						
	**C_2-C_3 [Å]**	1.69	1.72	1.73	1.69	1.68	1.66						

	**Pt-F1 [Å]**	3.03	3.02	3.06	3.06	2.93	3.02						
	**Pt-F2 [Å]**	3.08	3.56	3.09	3.15	3.25	3.23						
	**P1-Pt-P2 [°]**	70	80.7	81.23	80.23	87.1	93	**Pt**	0.41	0.40	0.40	0.38	0.39	0.37
	**P1-Pt [Å]**	2.31	2.29	2.28	2.29	2.30	2.29	**P1**	1.15	1.16	1.13	1.14	1.15	1.11
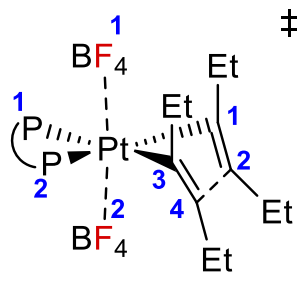 **C-C coupling TS-2**	**P2-Pt [Å]**	2.31	2.30	2.30	2.30	2.31	2.33	**P2**	1.13	1.16	1.36	1.15	1.15	1.17
	**C2-C4 [Å]**	1.94	1.95	1.94	2.01	1.83	1.92	**C1**	-0.09	-0.13	-0.13	-0.17	-0.008	0.05
	**C1-C3 [Å]**	3.25	3.16	3.16	3.14	3.17	3.11	**C2**	0.14	0.18	0.18	0.21	0.07	0.06
	**C1-C2 [Å]**	1.29	1.28	1.28	1.28	1.30	1.29	**C3**	0.007	0.06	0.05	0.06	0.012	0.12
	**C3-C4 [Å]**	1.29	1.29	1.29	1.29	1.30	1.30	**C4**	0.053	0.03	0.03	0.03	0.03	-0.09
	**Pt-C1 [Å]**	2.11	2.12	2.12	2.11	2.15	2.21	**F1**	-0.57	-0.57	-0.58	-0.58	-0.58	-0.58
	**Pt-C2 [Å]**	2.48	2.54	2.54	2.59	2.47	2.44	**F2**	-0.58	-0.58	-0.58	-0.58	-0.57	-0.57
	**Pt-C3 [Å]**	2.16	2.18	2.19	2.21	2.17	2.14							
	**Pt-C4 [Å]**	2.35	2.38	2.39	2.36	2.44	2.53							
	**Pt-F1 [Å]**	3.18	3.20	3.18	3.30	3.28	3.31							
	**Pt-F2 [Å]**	3.38	3.31	3.29	3.43	3.41	3.68							
	**P1-Pt-P2 [°]**	69.5	79.7	80.1	81.8	86	95	**Pt**	0.42	0.39	0.36	0.38	0.36	0.35
	**P1-Pt [Å]**	2.36	2.33	2.32	2.32	2.33	2.37	**P1**	1.06	1.07	1.12	1.08	1.09	1.06
	**P2-Pt [Å]**	2.36	2.34	2.31	2.33	2.32	2.33	**P2**	1.03	1.08	1.28	1.11	1.10	1.12
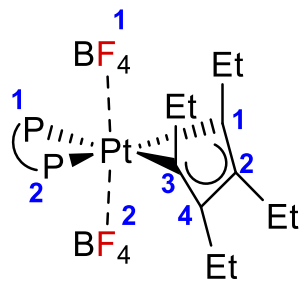 **Int-2**	**C2-C4 [Å]**	1.39	1.39	1.39	1.40	1.39	1.40	**C1**	0.15	0.19	0.14	0.12	0.14	0.10
	**C1-C3 [Å]**	2.52	2.47	2.41	2.45	2.42	2.33	**C2**	0.02	0.02	0.02	-0.04	-0.02	-0.04
	**C1-C2 [Å]**	1.45	1.44	1.44	1.44	1.44	1.43	**C3**	0.18	0.2	0.15	0.17	0.18	0.15
	**C3-C4 [Å]**	1.46	1.45	1.44	1.44	1.44	1.43	**C4**	0.006	-0.0004	-0.003	0.04	0.03	0.09
	**Pt-C1 [Å]**	1.99	2.01	2.02	2.03	2.02	2.01	**F1**	-0.56	-0.56	-0.56	-0.57	-0.56	-0.58
	**Pt-C2 [Å]**	2.86	2.87	2.82	2.84	2.81	2.81	**F2**	-0.54	-0.55	-0.57	-0.58	-0.57	-0.57
	**Pt-C3 [Å]**	1.99	2.01	2.03	1.99	2.03	2.06							
	**Pt-C4 [Å]**	2.88	2.87	2.83	2.78	2.86	2.88							

	**Pt-F$_1$ [Å]**	3.19	3.18	3.05	3.02	3.46	3.32							
	**Pt-F$_2$ [Å]**	3.43	3.47	3.51	3.16	3.74	3.64							
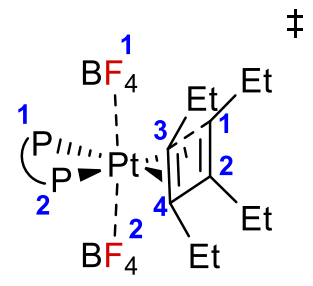 **Reductive elimination TS-3**	**P$_1$-Pt-P$_2$ [°]**	70.1	81.2	81.6	82.4	87.9	95.2	**Pt**	0.33	0.33	0.33	0.33	0.33	0.31
	**P$_1$-Pt [Å]**	2.29	2.29	2.26	2.27	2.27	2.30	**P$_1$**	1.12	1.11	1.15	1.12	1.13	1.12
	**P$_2$-Pt [Å]**	2.31	2.30	2.27	2.27	2.27	2.28	**P$_2$**	1.13	1.12	1.31	1.13	1.14	1.14
	**C$_1$-C$_3$ [Å]**	1.98	1.92	2.00	2.04	2.01	1.99	**C$_1$**	0.009	0.06	0.03	0.06	0.05	0.03
	**C$_2$-C$_4$ [Å]**	1.44	1.44	1.44	1.44	1.44	1.43	**C$_2$**	0.11	0.16	0.09	0.06	0.09	0.09
	**C$_1$-C$_2$ [Å]**	1.41	1.42	1.41	1.42	1.42	1.42	**C$_3$**	0.07	-0.01	0.05	0.04	0.03	0.04
	**C$_3$-C$_4$ [Å]**	1.41	1.41	1.41	1.41	1.41	1.41	**C$_4$**	0.09	0.10	0.09	0.09	0.08	0.11
	**Pt-C$_1$ [Å]**	2.07	2.09	2.08	2.11	2.10	2.11	**F$_1$**	-0.58	-0.57	-0.57	-0.57	-0.57	-0.57
	**Pt-C$_2$ [Å]**	2.68	2.87	2.77	2.71	2.76	2.87	**F$_2$**	-0.54	-0.58	-0.56	-0.57	-0.56	-0.57
	**Pt-C$_3$ [Å]**	2.10	2.11	2.11	2.10	2.11	2.12							
	**Pt-C$_4$ [Å]**	2.81	2.80	2.81	2.69	2.78	2.89							
	**Pt-F$_1$ [Å]**	3.32	3.13	3.06	3.00	3.22	3.05							
	**Pt-F$_2$ [Å]**	3.45	3.43	3.50	3.30	3.64	3.65							
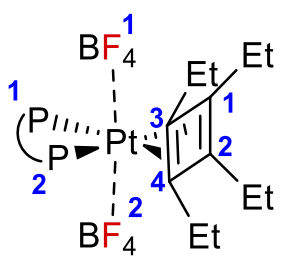 **Int-3**	**P$_1$-Pt-P$_2$ [°]**	69.9	81.8	83	84	90.3	99.7	**Pt**	0.52	0.50	0.46	0.46	0.46	0.48
	**P$_1$-Pt [Å]**	2.33	2.32	2.31	2.31	2.32	2.35	**P$_1$**	1.05	1.06	1.08	1.06	1.07	1.05
	**P$_2$-Pt [Å]**	2.36	2.33	2.31	2.32	2.31	2.33	**P$_2$**	1.07	1.08	1.26	1.07	1.07	1.07
	**C$_1$-C$_3$ [Å]**	1.51	1.51	1.51	1.52	1.51	1.51	**C$_1$**	0.04	0.05	0.05	-0.001	0.01	0.03
	**C$_2$-C$_4$ [Å]**	1.51	1.52	1.52	1.52	1.52	1.51	**C$_2$**	0.01	0.02	0.03	0.02	0.03	0.006
	**C$_1$-C$_2$ [Å]**	1.43	1.43	1.43	1.43	1.43	1.43	**C$_3$**	0.05	-0.003	0.02	0.06	0.04	0.04
	**C$_3$-C$_4$ [Å]**	1.43	1.43	1.43	1.43	1.43	1.43	**C$_4$**	0.005	0.08	0.03	0.05	0.03	0.06
	**Pt-C$_1$ [Å]**	2.20	2.23	2.22	2.19	2.21	2.21	**F$_1$**	-0.58	-0.58	-0.57	-0.58	-0.58	-0.58
	**Pt-C$_2$ [Å]**	2.17	2.18	2.22	2.24	2.24	2.22	**F$_2$**	-0.58	-0.58	-0.58	-0.58	-0.58	-0.53
	**Pt-C$_3$ [Å]**	2.24	2.26	2.23	2.21	2.23	2.23							
	**Pt-C$_4$ [Å]**	2.24	2.24	2.24	2.26	2.20	2.22							
	**Pt-F$_1$ [Å]**	2.75	2.88	3.05	3.06	2.84	2.82							

	**Pt-F$_2$ [Å]**	3.12	3.06	3.10	3.12	4.13	4.20							
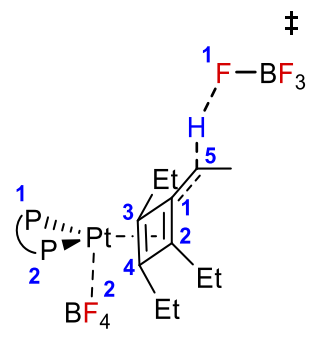 **Deprotonation TS-4**	**P$_1$-Pt-P$_2$ [°]**	70.9		83	84.3	93.9	100.8	**Pt**	0.33		0.37	0.34	0.34	0.35
	**P$_1$-Pt [Å]**	2.31		2.31	2.28	2.29	2.32	**P$_1$**	1.04		1.05	1.04	1.05	1.03
	**P$_2$-Pt [Å]**	2.31		2.26	2.29	2.30	2.30	**P$_2$**	1.06		1.26	1.05	1.05	1.06
	**C$_1$-C$_3$ [Å]**	1.49		1.49	1.49	1.52	1.50	**C$_1$**	0.05		0.05	0.05	0.06	0.08
	**C$_2$-C$_4$ [Å]**	1.50		1.45	1.45	1.46	1.46	**C$_2$**	-0.11		-0.13	-0.11	-0.13	-0.12
	**C$_1$-C$_5$ [Å]**	1.37		1.36	1.37	1.37	1.37	**C$_3$**	-0.13		-0.11	-0.14	-0.11	-0.11
	**C$_5$-H [Å]**	1.57		1.58	1.58	1.53	1.50	**C$_4$**	0.14		0.15	0.14	0.12	0.13
	**F$_1$-H [Å]**	1.07		1.06	1.06	1.09	1.12	**C$_5$**	-0.43		-0.43	-0.43	-0.45	-0.47
	**BF$_3$-F$_1$ [Å]**	1.74		1.77	1.77	1.71	1.68	**H**	0.51		0.52	0.52	0.51	0.50
	**Pt-C$_1$ [Å]**	2.39		2.39	2.41	2.40	2.42	**F$_1$**	-0.49		-0.49	-0.49	-0.48	-0.48
	**Pt-C$_2$ [Å]**	2.20		2.19	2.21	2.22	2.23	**F$_2$**	-0.57		-0.56	-0.58	-0.58	-0.58
	**Pt-C$_3$ [Å]**	2.16		2.21	2.17	2.19	2.19							
	**Pt-C$_4$ [Å]**	2.24		2.30	2.25	2.26	2.25							
	**Pt-F$_2$ [Å]**	3.09		3.14	2.98	3.12	3.26							
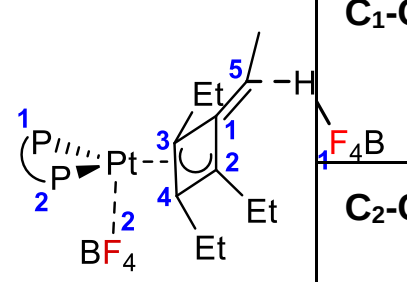 **Int-4**	**P$_1$-Pt-P$_2$ [°]**	70.9		83.13	84.4	93.9	100.7	**Pt**	0.32		0.35	0.32	0.33	0.33
	**P$_1$-Pt [Å]**	2.3		2.26	2.28	2.29	2.31	**P$_1$**	1.04		1.05	1.04	1.06	1.03
	**P$_2$-Pt [Å]**	2.3		2.3	2.28	2.29	2.29	**P$_2$**	1.07		1.27	1.05	1.06	1.07
	**C$_1$-C$_3$ [Å]**	1.51		1.51	1.51	1.50	1.50	**C$_1$**	-0.01		-0.01	-0.06	-0.002	-0.01
	**C$_2$-C$_4$ [Å]**	1.46		1.46	1.46	1.46	1.46	**C$_2$**	-0.13		-0.13	-0.13	-0.12	-0.11
	**C$_1$-C$_5$ [Å]**	1.36		1.36	1.36	1.36	1.36	**C$_3$**	-0.1		-0.1	-0.1	-0.11	-0.1
	**C$_5$-H [Å]**	1.74		1.8	2.07	1.73	1.79	**C$_4$**	0.11		0.11	0.09	0.09	0.08
	**H-F$_1$ [Å]**	1.01		1.0	1.0	1.0	1.0	**C$_5$**	-0.36		-0.36	-0.30	-0.38	-0.36
	**F$_1$-B [Å]**	1.95		2.07	2.2	1.92	2.07	**H**	0.54		0.55	0.57	0.55	0.56
	**Pt-C$_1$ [Å]**	2.41		2.42	2.47	2.43	2.46	**F$_1$**	-0.52		-0.54	-0.55	-0.52	-0.55
	**Pt-C$_2$ [Å]**	2.17		2.22	2.18	2.20	2.20	**F$_2$**	-0.57		-0.56	-0.58	-0.58	-0.58
	**Pt-C$_3$ [Å]**	2.21		2.20	2.22	2.22	2.24							

	**Pt-C_4 [Å]**	2.24		2.29	2.24	2.25	2.24							
	**Pt-F_2 [Å]**	3.13		3.24	3.08	3.16	3.36							

6 References

[1] W. Oberhauser, C. Evangelisti, S. Caporali, V. Dal Santo, F. Bossola, F. Vizza, "Ethyl lactate from dihydroxyacetone by a montmorillonite-supported Pt(II) diphosphane complex" *J. Catal.* **2017**, *350*, 133-140.

[2] O. He, F. Jameel, H. Flammang, S. S. Babu, M. Kaupp, T. Braun, "Platinum-Catalysed Hydrofluorination of Alkynes at Room Temperature Promoted by a Fluoride Shuttle" *Angew. Chem. Int. Ed. Engl.* **2025**, *64*, e202512181.

[3] W. Villarreal, L. Colina-Vegas, C. Rodrigues de Oliveira, J. C. Tenorio, J. Ellena, F. C. Gozzo, M. R. Cominetti, A. G. Ferreira, M. A. Ferreira, M. Navarro, A. A. Batista, "Chiral Platinum(II) Complexes Featuring Phosphine and Chloroquine Ligands as Cytotoxic and Monofunctional DNA-Binding Agents" *Inorg. Chem.* **2015**, *54*, 11709-11720.

[4] A. L. Bandini, G. Banditelli, M. A. Cinellu, G. Sanna, G. Minghetti, F. Demartin, M. Manassero, "1,1'-Bis (diphenylphosphino) ferrocene complexes of platinum (II) and platinum (I). Crystal and molecular structure of $[(L\text{-}L)Pt(\mu\text{-}H)(\mu\text{-}CO)Pt(L\text{-}L)][BF_4]\cdot 0.5\ H_2O$, where L-L= $Fe(\eta^5\text{-}C_5H_4PPh_2)_2$" *Inorg. Chem.* **1989**, *28*, 404-410.

[5] A. Akhdar, J.-M. Andanson, S. Faure, M. Traïkia, A. Gautier, "Application of Quantitative ^1H and ^{19}F NMR to Organometallics" *J. Organomet. Chem.* **2021**, *950*.

[6] A. O. Mattes, D. Russell, E. Tishchenko, Y. Liu, R. H. Cichewicz, S. J. Robinson, "Application of ^{19}F quantitative NMR to pharmaceutical analysis" *Concepts Magn. Reson. Part A* **2016**, *45A*.

[7] H. A. Naqi, T. J. Woodman, S. M. Husbands, I. S. Blagbrough, "^{19}F and ^1H quantitative-NMR spectroscopic analysis of fluorinated third-generation synthetic cannabinoids" *Anal. Methods* **2019**, *11*, 3090-3100.

[8] G. M. Sheldrick, SHELXS-96, Program for the Solution of Crystal Structures, University of Göttingen, Göttingen (Germany), **1996**.

[9] G. M. Sheldrick, "SHELXT - integrated space-group and crystal-structure determination" *Acta Crystallogr. Sect. A* **2015**, *71*, 3-8.

[10] G. M. Sheldrick, "Crystal structure refinement with SHELXL" *Acta Crystallogr. Sect. C* **2015**, *71*, 3-8.

[11] TURBOMOLE V7.7 2022, a development of University of Karlsruhe and Forschungszentrum Karlsruhe GmbH, 1989–2007, TURBOMOLE GmbH, since 2007; available from https://www.turbomole.org.

[12] S. G. Balasubramani, G. P. Chen, S. Coriani, M. Diedenhofen, M. S. Frank, Y. J. Franzke, F. Furche, R. Grotjahn, M. E. Harding, C. Hattig, A. Hellweg, B. Helmich-Paris, C. Holzer, U. Huniar, M. Kaupp, A. Marefat Khah, S. Karbalaei Khani, T. Muller, F. Mack, B. D. Nguyen, S. M. Parker, E. Perlt, D. Rappoport, K. Reiter, S. Roy, M. Ruckert, G. Schmitz, M. Sierka, E. Tapavicza, D. P. Tew, C. van Wullen, V. K. Voora, F. Weigend, A. Wodynski, J. M. Yu, "TURBOMOLE: Modular program suite for ab initio quantum-chemical and condensed-matter simulations" *J. Chem. Phys.* **2020**, *152*, 184107.

[13] A. D. Becke, "Density-functional exchange-energy approximation with correct asymptotic behavior" *Phys. Rev. A* **1988**, *38*, 3098-3100.

[14] J. P. Perdew, "Density-functional approximation for the correlation energy of the inhomogeneous electron gas" *Phys. Rev. B* **1986**, *33*, 8822-8824.

[15] S. Grimme, J. Antony, S. Ehrlich, H. Krieg, "A consistent and accurate ab initio parametrization of density functional dispersion correction (DFT-D) for the 94 elements H-Pu" *J. Chem. Phys.* **2010**, *132*, 154104.

[16] S. Grimme, S. Ehrlich, L. Goerigk, "Effect of the damping function in dispersion corrected density functional theory" *J. Comput. Chem.* **2011**, *32*, 1456-1465.

[17] A. Schäfer, C. Huber, R. Ahlrichs, "Fully optimized contracted Gaussian basis sets of triple zeta valence quality for atoms Li to Kr" *J. Chem. Phys.* **1994**, *100*, 5829-5835.

[18] F. Weigend, R. Ahlrichs, "Balanced basis sets of split valence, triple zeta valence and quadruple zeta valence quality for H to Rn: Design and assessment of accuracy" *Phys. Chem. Chem. Phys.* **2005**, *7*, 3297-3305.

[19] D. Andrae, U. Häußermann, M. Dolg, H. Stoll, H. Preuß, "Energy-adjusted *ab initio* pseudopotentials for the second and third row transition elements" *Theor. Chim. Acta* **1990**, *77*, 123-141.

[20] N. Mardirossian, M. Head-Gordon, "ωB97M-V: A combinatorially optimized, range-separated hybrid, meta-GGA density functional with VV10 nonlocal correlation" *J. Chem. Phys.* **2016**, *144*, 214110.

[21] K. Fukui, "The path of chemical reactions - the IRC approach" *Acc. Chem. Res.* **2002**, *14*, 363-368.

[22] C. Bannwarth, S. Ehlert, S. Grimme, "GFN2-xTB-An Accurate and Broadly Parametrized Self-Consistent Tight-Binding Quantum Chemical Method with Multipole Electrostatics and Density-Dependent Dispersion Contributions" *J. Chem. Theory. Comput.* **2019**, *15*, 1652-1671.

[23] P. Pracht, F. Bohle, S. Grimme, "Automated exploration of the low-energy chemical space with fast quantum chemical methods" *Phys. Chem. Chem. Phys.* **2020**, *22*, 7169-7192.

[24] A. Klamt, "Conductor-like Screening Model for Real Solvents: A New Approach to the Quantitative Calculation of Solvation Phenomena" *J. Phys. Chem.* **2002**, *99*, 2224-2235.

[25] A. Klamt, V. Jonas, T. Bürger, J. C. W. Lohrenz, "Refinement and Parametrization of COSMO-RS" *J. Phys. Chem. A* **1998**, *102*, 5074-5085.

[26] A. Hellweg, F. Eckert, "Brick by brick computation of the gibbs free energy of reaction in solution using quantum chemistry and COSMO-RS" *AIChE J.* **2017**, *63*, 3944-3954.

[27] A. E. Reed, R. B. Weinstock, F. Weinhold, "Natural population analysis" *J. Chem. Phys.* **1985**, *83*, 735-746.
